# Improved Salinity Tolerance-Associated Variables Observed in EMS Mutagenized Wheat Lines

**DOI:** 10.3390/ijms231911386

**Published:** 2022-09-27

**Authors:** Johanna Lethin, Caitlin Byrt, Bettina Berger, Chris Brien, Nathaniel Jewell, Stuart Roy, Hesam Mousavi, Selvakumar Sukumaran, Olof Olsson, Henrik Aronsson

**Affiliations:** 1 Department of Biological and Environmental Sciences, University of Gothenburg, SE-405 30 Gothenburg, Sweden; 2 Division of Plant Sciences, Research School of Biology, College of Science, Australian National University, Acton, ACT 2601, Australia; 3 Australian Plant Phenomics Facility, University of Adelaide, Waite Campus, Urrbrae, SA 5064, Australia; 4 School of Agriculture, Food and Wine, University of Adelaide, Waite Campus, Urrbrae, SA 5064, Australia; 5 Department of Agricultural Sciences, Inland Norway University of Applied Sciences, Postboks 400, 2418 Elverum, Norway; 6 Department of Pure and Applied Biochemistry, University of Lund, SE-221 00 Lund, Sweden

**Keywords:** EMS, mutagenized, salinity, salt stress, Smarthouse, sustainable agriculture, wheat

## Abstract

Salinity tolerance-associated phenotypes of 35 EMS mutagenized wheat lines originating from BARI Gom-25 were compared. Vegetative growth was measured using non-destructive image-based phenotyping. Five different NaCl concentrations (0 to 160 mM) were applied to plants 19 days after planting (DAP 19), and plants were imaged daily until DAP 38. Plant growth, water use, leaf Na^+^, K^+^ and Cl^−^ content, and thousand kernel weight (TKW) were measured, and six lines were selected for further analysis. In saline conditions, leaf Na^+^, K^+,^ and Cl^−^ content variation on a dry weight basis within these six lines were ~9.3, 1.4, and 2.4-fold, respectively. Relative to BARI Gom-25, two (OA6, OA62) lines had greater K^+^ accumulation, three (OA6, OA10, OA62) had 50–75% lower Na^+^:K^+^ ratios, and OA62 had ~30% greater water-use index (WUI). OA23 had ~2.2-fold greater leaf Na^+^ and maintained TKW relative to BARI Gom-25. Two lines (OA25, OA52) had greater TKW than BARI Gom-25 when grown in 120 mM NaCl but similar Na^+^:K^+^, WUI, and biomass accumulation. OA6 had relatively high TKW, high leaf K^+,^ and WUI, and low leaf Na^+^ and Cl^−^. Phenotypic variation revealed differing associations between the parameters measured in the lines. Future identification of the genetic basis of these differences, and crossing of lines with phenotypes of interest, is expected to enable the assessment of which combinations of parameters deliver the greatest improvement in salinity tolerance.

## 1. Introduction

Soil salinization is an escalating problem worldwide. The Food and Agriculture Organization of the United Nations reported that salt-affected soils are considered as being soils at or above 2 dS/m electrical conductivity and that worldwide salt-affected topsoil (0–30 cm) is 4.4% (424 Mha) and subsoil (30–100 cm) is 8.7% (833 Mha) of the total global area land [1]. Salinity reduces crop yields, which limits economic gains and increases hunger and poverty [2,3,4]. Therefore, changes to farming practices and landscape planning in combination with focused plant breeding programs to achieve adaptive plants resistant to salt-affected soils are needed [3,5,6]. One important step in achieving productivity on saline soils involves developing salt tolerant crop varieties [3,6]. In this case, developing salt tolerant wheat is the focus, and the approach here involves seeking novel allelic variation in a mutant population.

Mutagenesis using ethyl methane sulphonate (EMS) was recently used to produce a mutagenized *Triticum aestivum* (wheat) population to identify lines with increased salt tolerance. The EMS mutagenesis was applied to a Bangladeshi wheat variety designated BARI Gom-25 [7]. This variety is tolerant to leaf rust, heat, and moderate salt levels [8,9,10]. BARI Gom-25 is moderately salt tolerant, so if it can be improved to withstand higher salt levels in soils, it would be a valuable resource to bring back productivity in saline coastal areas in Bangladesh. Around 30% of the cultivable land in Bangladesh is in the coastal area, and approximately 1/3 of 2.9 Mha of coastal and offshore lands are affected by salinity [11]. Large areas of the lands, therefore, remain fallow in the dry winter season (November to April) due to the salinity.

When setting up the original screening assay to test for salt tolerance, the aim was to identify lines from the mutagenized population that could germinate on filter paper on Petri dishes with high salinity (200 mM NaCl). After screening 1676 lines from the population, 70 lines (OA-lines) were identified that germinated and grew on the saline media. In contrast, the reference variety BARI Gom-25 did not germinate under these conditions. The initial screening work was followed up using these selected lines in field trials in the South of Bangladesh, where the soil salinity started at ca. 7.2 dS/m and reached ca. 9.8 dS/m. This test showed that all 70 OA-lines performed better than BARI Gom-25 concerning germination and subsequent growth [7]. Thus, some of these wheat lines could fill a gap in crop production during winter fallow in Bangladesh when the salt content increases as the affected soils dry up. However, whether the most promising OA-lines also perform well enough in soils where the salt level is initially low and then increases rapidly during the growth period remains to be evaluated. This raised the question of whether the lines would differ in any defined salt tolerance parameters if they experienced elevated saline conditions later in their development. A recent study compared 23 of these wheat lines using soil salinity induced by controlled drip irrigation in moderate European outdoor climatic conditions [12]. It showed that nine lines had a 2-fold better total grain weight than the control. However, it did not consider growth rate, ion uptake or distribution, or water use which are all critical variables for salt tolerance.

High throughput phenotypic screening has become a powerful tool for identifying biotic or abiotic stress responses during plant growth. Image-based phenotyping offers a non-destructive approach to screening a large population of plants over time. This captures differences in phenotypes not easily detectable by the human eye, such as plant biomass, leaf area, water use efficiency, growth rate, or transpiration [13,14,15,16,17]. In order to withstand salt stress, salt tolerant plants need to adapt one or several traits like increased osmotic pressure, increased exclusion of ions that are detrimental to productivity when in excess, such as Na^+^ and Cl^−^, and compartmentalization of ions such as Na^+^ and Cl^−^ into cell organelles [18,19,20,21].

During salt stress, the regulation of several salt stress-responsive genes changes, leading to stress acclimation in stress-tolerant crops. Some differentially regulated genes play direct roles in protecting the plant from stress; however, others induce various signaling pathways associated with differences in reactive oxygen species, lipid phosphatase, and cyclic nucleotides [18,19,20]. The effect of salt stress is divided into two phases; the first phase is the osmotic effect that is not dependent on ion accumulations in the shoot. Salt-affected soil has a relatively low water potential that limits the efficient water uptake from roots [22]. Lack of water sends signals from the root, where the initial signal perception occurs, to the shoot to reduce transpiration by closing the stomata, followed by reduced cell expansion. As transpiration is the driving force for CO_2_ uptake, closing stomata automatically decreases photosynthetic activity, reducing both growth and final yield [18,19,20]. The second phase relates to the accumulation of Na^+^ and Cl^−^ in the plant following uptake from the salt-affected soil. Plants must either exclude excess salt ions from reaching the shoot or compartmentalize them inside the cell, most commonly in the vacuole, to limit their toxic effect. Some salt tolerant plants exclude Na^+^ and Cl^−^ from leaves by limiting root uptake or root-to-shoot transport, or plants accumulate compatible solutes in their cytoplasm, e.g., proline, glycine betaine, and polyamines, to osmotically balance the toxic ions accumulated in the vacuole [19,20,23]. Thus, measuring Na^+^ and Cl^−^ in the shoot to check for differences in ion exclusion between BARI Gom-25 and the mutagenized lines is of interest. As Na^+^ has similar physiochemical properties as K^+^, at toxic concentrations, Na^+^ will outcompete K^+^ for enzyme binding sites. K^+^ is important for several vital cellular processes, such as the activation of around 60 different enzymes [24]. Hence, an increased cellular Na^+^:K^+^ ratio is a negative parameter and can disrupt the ion balance needed to fulfill essential metabolic processes both in root and shoot tissue [18,19,20]. Moreover, the accumulation of Cl^−^ and Na^+^ ions in the cell can disrupt charge balance and osmoregulation. To tolerate salt stress, a low Na^+^:K^+^ ratio is preferable to maintain reactions in the cell that require K^+^ [18,19,20]. Therefore, testing the Na^+^:K^+^ ratio in the different mutant lines and BARI Gom-25 is of interest to assess whether this ratio may be a factor in the differences in salt tolerance.

Water uptake and transpiration are needed for plant growth and photosynthesis. Increased levels of NaCl in the soil negatively affect water uptake. Na^+^ and Cl^−,^ when accumulated in the root and shoot, will lead to water stress reactions that will harm plant development and final yield [20,25,26]. Thus, measurements of water use (WU) per day in the mutant lines are of interest, and by combing this data with predicted biomass data, it is possible to obtain a water use index (WUI). The WUI (or transpiration index) is based on the biomass produced per unit of water used by the crop. It has been shown in *Oryza sativa* (rice) that during vegetative growth under saline conditions, it is crucial to maintain a good WUI to achieve salinity tolerance [14]. Thus, higher WUI corresponds to higher water efficiency overall. For salt tolerance, a high WUI value indicates whether the plants can reach sufficient water uptake levels needed for various cellular reactions and survive despite non-optimal soil conditions.

The current study aims to investigate different sub-traits behind the previously identified salt tolerance of EMS mutagenized wheat lines. Using non-destructive imaging, we determined shoot growth under different salinity concentrations over time. We hypothesized that different EMS lines differed in their combination of salinity tolerance sub-traits contributing to varying levels of overall tolerance. This type of datum is expected to enable the characterization of the possible combinations of traits that may be useful for future breeding programs focused on breeding wheat to withstand salt-affected soils or transfer traits to other species suitable to remediate saline soils. In addition, further molecular studies to resolve point mutation(s) within genes related to the sub-traits will provide valuable molecular SNP markers.

## 2. Results

### 2.1. Selection of Mutagenized Lines for In-Depth Study

A series of phenotypic parameters of interest for 35 mutant wheat lines in a BARI Gom-25 background and BARI Gom-25 as control were measured (Supplementary Figures S1–S41). Supplementary Table S1 shows that there were differences between the lines and between the salinity treatments for all parameters, except the first. However, only for some parameters was the interaction significant (*p* ≤ 0.05) so that, for these parameters, the differences between the salinity treatments varied between lines. Based on the first preliminary results from phenotypic observations and thousand kernel weight (TKW) measurements of the lines compared to BARI Gom-25, we selected six mutagenized lines, OA6, OA10, OA23, OA25, OA52, and OA62 (Supplementary Figures S1–S41), for additional phenotypic analyses. They represented potential different phenotypic patterns in one or several of the parameters of interest; predicted biomass, leaf Na^+^, K^+^ and Cl^−^ content, water usage, and TKW.

For most phenotypic analyses, there were multiple intervals and time points where measures were taken to assess trends over time. During the osmotic phase, relative maintenance of shoot growth is of interest. This stage occurs early after NaCl application; hence we used DAP 19–23. Leaf ion concentration was measured on leaves harvested over DAP 39–40. We decided to use DAP 38 and the interval DAP 32–38 as the base for the other measures. DAP 38 is the end of the three-week imaging period by which the plants have experienced salinity stress. Tests to check for statistical differences in data values for different lines were applied to data from a range of DAP intervals and DAP 38 (Supplementary Figures S42 and S43).

### 2.2. Mutagenized Wheat Lines Differed in Shoot Size in Response to Salinity

#### Smoothed Estimated Plant Biomass (sEPB) Absolute Growth Rate (AGR)

Smoothed estimated plant biomass (sEPB) absolute growth rate (AGR) was measured to study the increase in mass over time. The general pattern was that sEPB AGR increased steadily over time at 0 mM NaCl for all lines except OA10, which reached a plateau at the end interval DAP32–38 (Supplementary Figure S42). The kpixels/day increase was also observed at 40 mM NaCl but less pronounced. OA23 and OA25 reached a plateau in interval DAP 32–38, whereas OA10 declined (Supplementary Figure S42). OA6 and OA62 biomass increased over time at 80 mM NaCl except in interval DAP 32–38, where OA62 declined. Other lines and the control stayed at the same sEPB AGR values over time at 80 mM NaCl. At elevated NaCl (120 mM and 160 mM), the kpixels/day increase was more or less absent over time, except for a slight increase at 160 mM NaCl for OA62. Otherwise, the kpixels/day values remained stable except for OA10, which had an apparent decline in kpixels/day from the interval DAP 23–28 onwards (Supplementary Figure S42).

**Figure 1:**
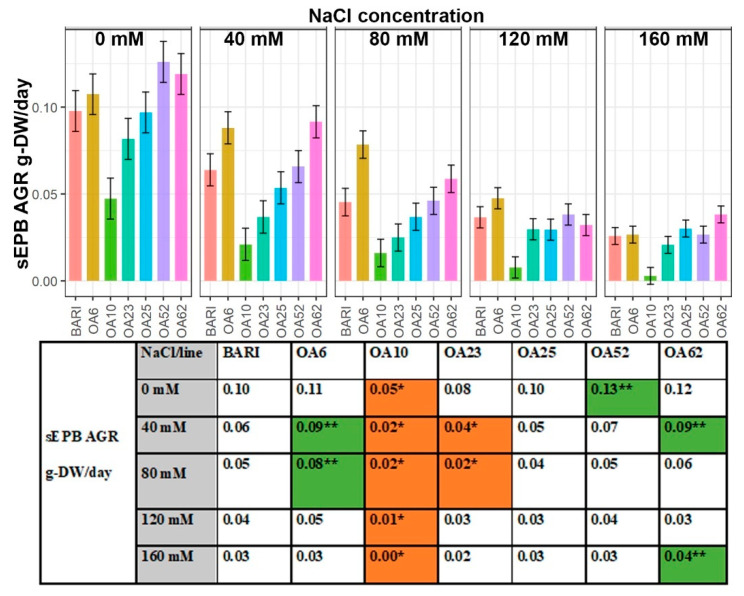
**Absolute growth rate of estimated plant biomass (sEPB AGR) in interval DAP 32–38.** Data are shown for the control BARI Gom-25 (BARI) and mutagenized wheat lines OA6, OA10, OA23, OA25, OA52, and OA62 in interval DAP 32–38. NaCl concentrations applied were in the range from 0 mM to 160 mM. Upper panel: sEPB AGR values with half-LSD error bars. Lower panel: table high-lighting the sEPB AGR mean values with color codes for significantly higher (dark green **) or lower (orange *) values compared to BARI Gom-25, *p* ≤ 0.05.

In the interval, DAP32–38 BARI Gom-25 gained 0.10 g-DW/day at 0 mM NaCl, but the gain was only 0.03 g-DW/day at 160 mM NaCl (Figure 1). OA6 and OA62 showed either non-significant or significantly higher sEPB AGR g-DW/day values than the control at all NaCl concentrations except for 160 mM NaCl, where OA6 was similar, and at 120 mM NaCl, where OA62 was lower than the control. At 40 mM NaCl, OA6, and OA62 had 50% higher AGR than BARI Gom-25; 0.09 g-DW/day versus 0.06 g-DW/day (Figure 1). OA23 never reached similar values as BARI Gom-25, and OA10 was significantly lower at all NaCl concentrations.

### 2.3. Large Variation Observed in Leaf Na^+^, K^+,^ and Cl^−^ Content on a Dry Weight Basis within the Selected Mutagenized Lines

The K^+^, Na^+^, and Cl^−^ content of flag leaves was determined at DAP 39–40 for all mutagenized lines and the control.

#### 2.3.1. K^+^ Content Based on Dry Weight

**Figure 2:**
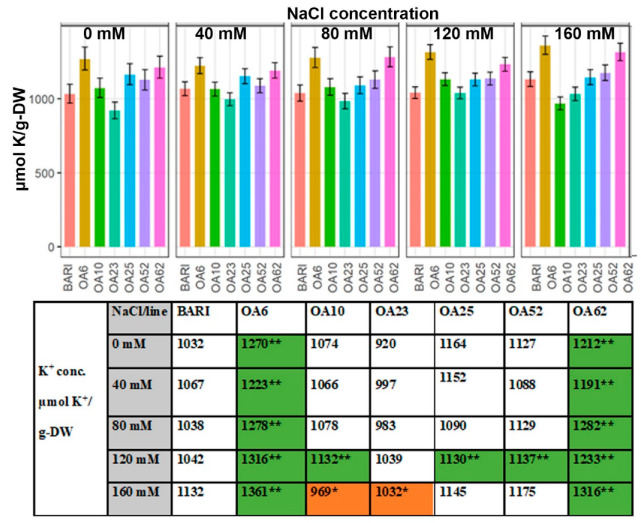
**Dry weight (DW) concentration of K^+^ at DAP 39–40.** Data are shown for the control BARI Gom-25 (BARI) and mutagenized wheat lines OA6, OA10, OA23, OA25, OA52, and OA62 at DAP 39–40. NaCl concentrations applied were in the range from 0 mM to 160 mM. Upper panel: K^+^ values with half-LSD error. Lower panel: table high-lighting the K^+^ mean values with color codes for significantly higher (dark green **) or lower (orange *) values compared to BARI Gom-25, *p* ≤ 0.05.

OA6 and OA62 maintained significantly greater leaf K^+^ content than BARI Gom-25 at all NaCl concentrations (Figure 2). OA6 and OA62 could accumulate more K^+^ than BARI Gom-25 when grown in 0 mM NaCl, which might be why they also accumulated more K^+^ when NaCl was applied. At 0 mM, BARI Gom-25 had 1032 µmol K^+^/g-DW, whereas OA6 and OA62 had 1270 µmol K^+^/g-DW and 1212 µmol K^+^/g-DW, respectively; and when grown in 160 mM NaCl, BARI Gom-25 had 1132 µmol K^+^/g-DW whereas OA6 and OA62 had 1361 µmol K^+^/g-DW and 1316 µmol K^+^/g-DW, respectively. OA25 and OA52 accumulated more K^+^ at the higher NaCl levels than BARI Gom-25; for example, at 120 mM NaCl OA25 and OA52 had 1130 µmol K^+^/g-DW, and 1137 µmol K^+^/g-DW, respectively, relative to 1042 µmol K^+^/g-DW for BARI Gom-25. OA10 had a similar pattern as OA25 and OA52 but had less K^+^ accumulation when grown in 160 mM NaCl (969 µmol/g-DW), and OA23 had 1032 µmol K^+^/g-DW when grown in 160 mM NaCl. OA23 was the only line that accumulated fewer K^+^ than BARI Gom-25 in all NaCl treatments (Figure 2).

#### 2.3.2. Na^+^ Content Based on Dry Weight

**Figure 3:**
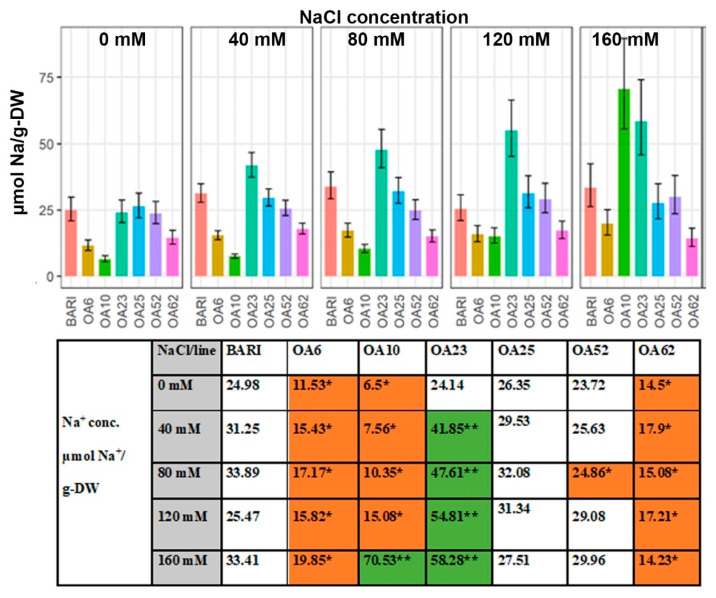
**Dry weight (DW) concentration of Na^+^ at DAP 39–40.** Data are shown for the control BARI Gom-25 (BARI) and mutagenized wheat lines OA6, OA10, OA23, OA25, OA52, and OA62 at DAP 39–40. NaCl concentrations applied were in the range from 0 mM to 160 mM. Upper panel: Na^+^ values with half-LSD error. Lower panel: table high-lighting the Na^+^ mean values with color codes for significantly higher (dark green **) or lower (orange *) values compared to BARI Gom-25, *p* ≤ 0.05.

BARI Gom-25 had a leaf Na^+^ content of 24.98 µmol Na^+^/g-DW at 0 mM NaCl, and OA25 was the only line with a higher value, 26.35 µmol Na^+^/g-DW, whereas OA10 had the lowest value, almost 4-fold lower, 6.5 µmol Na^+^/g-DW (Figure 3). When NaCl was applied, the initial baseline values observed at 0 mM NaCl increased for all lines. OA6, OA10, and OA62 all had significantly lower values than BARI Gom-25 for all NaCl levels except in one case at 160 mM NaCl, where OA10 had a significantly higher Na^+^ value, 70.53 µmol Na^+^/g-DW vs. 33.41 µmol Na^+^/g-DW for the BARI Gom-25. Overall, these lines showed less (ca. 35–75%) Na^+^ accumulation compared to BARI Gom-25 in the treatment range of 40 to 120 mM NaCl. At 80 mM NaCl, OA52 had a significantly lower value, 24.80 µmol Na^+^/g-DW, than BARI Gom-35, 33.89 µmol Na^+^/g-DW. OA23 had higher Na^+^/g-DW values than BARI Gom-25 (ca. 50–110% higher) when NaCl was applied (Figure 3).

#### 2.3.3. Cl^−^ Content Based on the Dry Weight

**Figure 4:**
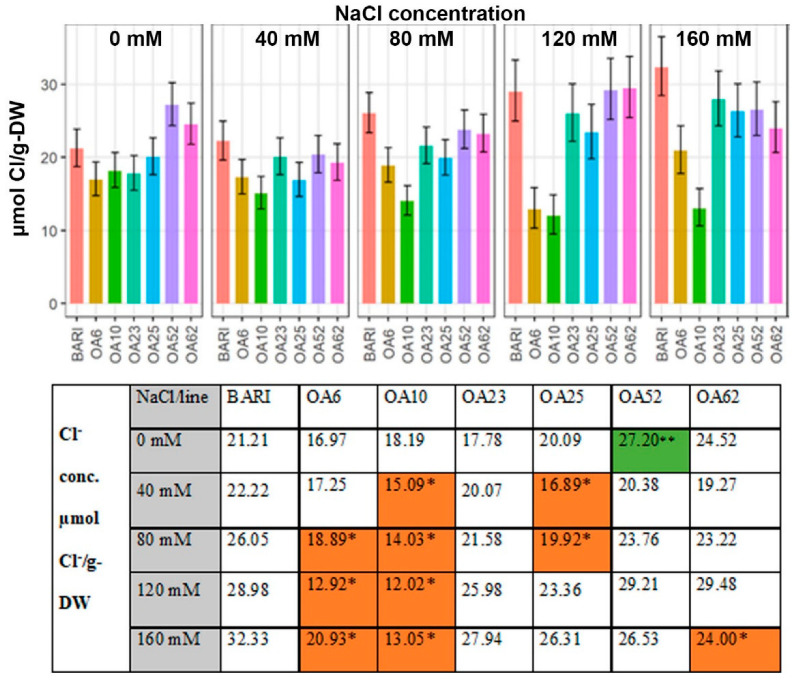
**Dry weight (DW) concentration of Cl^−^ at DAP 39–40.** Data are shown for the control BARI Gom-25 (BARI) and mutagenized wheat lines OA6, OA10, OA23, OA25, OA52, and OA62 at DAP 39–40. NaCl concentrations applied were in the range from 0 mM to 160 mM. Upper panel: Cl^−^ values with half-LSD error bars. Lower panel: table high-lighting the Cl^−^ mean values with color codes for significantly higher (dark green **) or lower (orange *) values compared to BARI Gom-25, *p* ≤ 0.05.

At 0 mM NaCl DAP 39 and 40, BARI Gom-25 leaves had 21.21 µmol Cl^−^/g-DW (Figure 4). Four lines had lower values, and two had higher values, with OA52 being significantly higher with 27.20 µmol/g-DW. The lowest value at 0 mM NaCl was observed for OA6 being 16.97 µmol Cl^−^/g-DW. For the remaining NaCl levels, all lines had lower or significantly lower values than BARI Gom-25 except OA52 and OA62 at 120 mM NaCl, with values similar to BARI Gom-25 (Figure 4). OA10 had lower values following treatments of 40 mM to 160 mM NaCl; OA6 had lower values following 80 mM to 160 mM NaCl treatments, and OA62 had lower values following the 160 mM NaCl treatment. The overall pattern was an increase in Cl^−^ accumulation with each higher NaCl applied, e.g., BARI Gom-25 leaves ranged from 21.21 µmol Cl^−^/g-DW (0 mM NaCl) to 32.33 µmol Cl^−^/g-DW (160 mM NaCl). OA10 ranged from 18.19 µmol Cl^−^/g-DW 8 (0 mM NaCl) to 13.05 µmol Cl^−^/g-DW (160 mM NaCl) and had half or less Cl^−^ accumulated in the leaves at 80–160 mM NaCl than BARI Gom-25 (Figure 4).

#### 2.3.4. Leaf Na^+^:K^+^ Ratio

**Figure 5:**
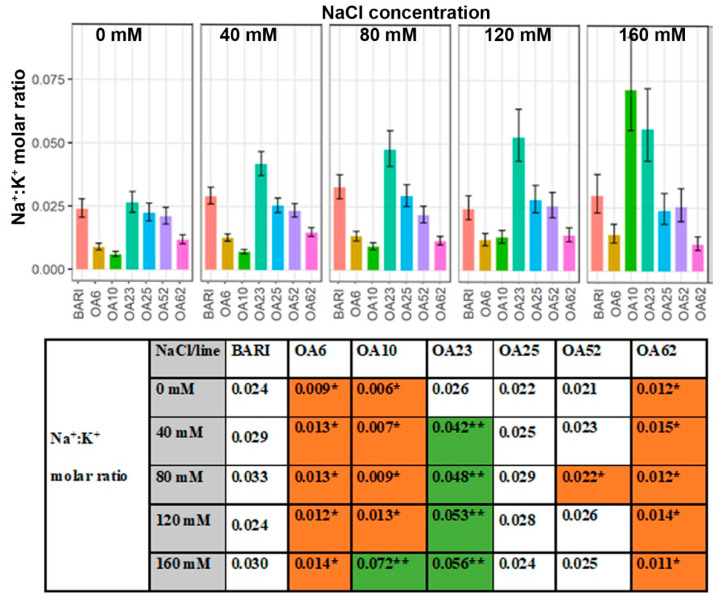
**Na^+^:K^+^ molar ratio at DAP 39–40.** Data are shown for the control BARI Gom-25 (BARI) and mutagenized wheat lines OA6, OA10, OA23, OA25, OA52, and OA62 at DAP 39–40. NaCl concentrations applied were in the range from 0 mM to 160 mM. Upper panel: Na^+^:K^+^ ratio values with half-LSD error bars. Lower panel: table high-lighting the Na^+^:K^+^ ratio mean values with color codes for significantly higher (dark green **) or lower (orange *) values compared to BARI Gom-25, *p* ≤ 0.05.

The lowest Na^+^:K^+^ molar ratio obtained for BARI Gom-25 was 0.024 at 0 mM and 120 mM NaCl, and the highest ratio observed for BARI Gom-25 was 0.033 at 80 mM NaCl (Figure 5). OA6 and OA62 had relatively low Na^+^:K^+^ ratios throughout all NaCl levels applied, with ratios within the range of 0.011 to 0.015 at 40 mM to 150 mM NaCl. OA10 had lower ratios than OA6 and OA62 between 0 mM to 80 mM NaCl, which were in the range of 0.006 to 0.009 (Figure 5). However, OA10 had a higher ratio than BARI Gom-25 at 160 mM NaCl, 0.072 vs. 0.030. OA52 had a significantly lower ratio at 80 mM NaCl, 0.022, compared to BARI Gom-25, 0.033. The Na^+^:K^+^ ratios for OA23 were all higher than those of BARI Gom-25 in 40 mM to 160 mM NaCl. The highest value of OA23 was observed at 160 mM, 0.056, and the lowest at 0 mM NaCl, 0.026 (Figure 5).

### 2.4. The Water Use Index (sWUI) Was Greater in Two Lines, whereas One Line Was Less Efficient

**Figure 6:**
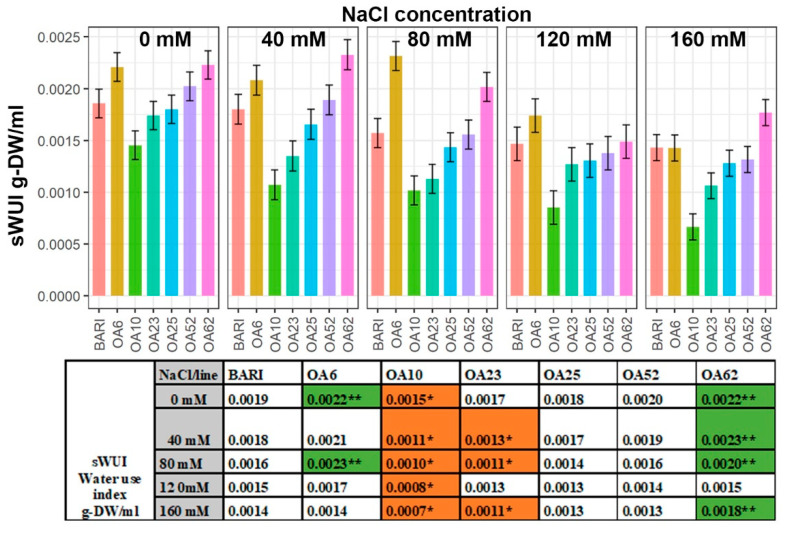
**Water use index (sWUI) throughout the imaging period DAP 19–38.** Data are shown for the control BARI Gom-25 (BARI) and mutagenized wheat lines OA6, OA10, OA23, OA25, OA52, and OA62 over DAP 19–39. NaCl concentrations applied were in the range from 0 mM to 160 mM. Upper panel: sWUI values with half-LSD error bars. Lower panel: table high-lighting the sWUI mean values with color codes for significantly higher (dark green **) or lower (orange *) values compared to BARI Gom-25, *p* ≤ 0.05.

Calculating the water use index (WUI, or transpiration index) takes into account the biomass produced, which helps estimate the efficiency of the water used for carbon assimilation. Here, it was defined as the ratio of the daily change in sEPB to the daily WU, i.e., sWUI. BARI Gom-25 had its highest value at 0 mM NaCl, 0.0019 g-DW/mL, and lowest value at 160 mM NaCl, 0.0014 g-DW/mL (Figure 6). OA62 was significantly higher in sWUI than BARI Gom-25 following all salt treatments except for the 120 mM NaCl treatment. OA6 and OA62 reached their highest sWUI values at 80 mM NaCl (0.0023 g-DW/mL) and at 40 mM NaCl (0.0023 g-DW/mL), respectively. OA23 never had higher values than BARI Gom-25, and at 80 mM NaCl, it had approximately 30% lower sWUI than BARI Gom-25. All sWUI values were significantly lower for OA10 than BARI Gom-25, with OA10’s lowest sWUI value following the 160 mM NaCl treatment, 0.007 g-DW/mL, which was 50% lower than the BARI Gom-25 sWUI (Figure 6).

### 2.5. Several Lines Had Higher TKW Than the Control at Higher Salt Levels

The TKW data were collected from plants harvested at DAP 99. BARI Gom-25 had a TKW of 51.65 g at 0 mM NaCl, and following NaCl treatments, the weight decreased to 42.36 g at 160 mM NaCl. OA25 had constantly higher TKW than BARI Gom-25 following the NaCl treatments, with a major difference following the 120 mM NaCl treatment, 58.11 g versus 40.81 g. However, for the other NaCl treatments, the OA25 only reached values 10% greater than BARI Gom-25 (160 mM, Table 1). OA52 had higher TKW at 0 mM than BARI Gom-25 and followed a similar pattern to that of OA25, with the greatest difference observed following the 120 mM NaCl treatment (58.26 g versus 40.81 g) and overall higher values except for following the 40 mM NaCl treatment. OA6 had higher TKW values, except for 40 mM NaCl, following the salt treatments than BARI Gom-25. These were in the ca. 1.0–10.4% range, except at 120 mM NaCl, where the difference was 17% higher. OA23 had higher TKW than BARI Gom-25 at 0 mM NaCl of 46.8 g, then remained somewhat similar following 40 mM and 160 mM NaCl throughout increased salt treatments in the range of 22.7–30.8%. OA62 had low initial seed weight values at 0 mM NaCl, 46.48 g, and remained ca. 5–10% lower at all NaCl levels except for 120 mM NaCl, with a slightly higher TKW, 41.57 g, relative to BARI Gom-25. OA10 had lower TKWs than all the other lines and BARI Gom-25. From its initial TKW of 29.50 g at 0 mM NaCl, values for OA10 decreased to 25.86 g at 160 mM NaCl. When applying the ANOVA test and grouping the data using pairwise comparison of the overall dataset for all salt concentrations, OA23 and OA25 stood out, followed by OA52 and OA6. After that, BARI Gom-25 and OA62, with OA10 being on its own with the lowest values (Table 1).

## 3. Discussion

To explore different salt stress phases and investigate the mechanisms the mutagenized lines used to achieve salt tolerance, we selected a non-destructive image-based phenotyping approach. Non-destructive image-based phenotyping of large sets of different varieties has previously been a helpful approach to scrutinizing differences in salt-stress responses between different genotypes during vegetative growth stages [16,17,27,28,29,30]. Therefore, data for predicted biomass from the image-based phenotyping combined with measuring tissue ion concentrations and water use values were analyzed to compare the relative performance of different mutant lines of interest and BARI Gom-25 as control.

### 3.1. OA6 and OA62 Had Improved Growth during Osmotic and Ion Stress

The magnitudes of growth were investigated over a longer period of salinity stress, DAP 19–38, by measuring the difference in size, performance, and rate of new dry mass accumulation for the plants per unit of already existing dry mass. These growth values indicate relative plant health and development. Not surprisingly, we observed reduced biomass in salt stress conditions compared to without NaCl added, in line with previous studies (Figure 1, Supplementary Figure S42) [31,32,33]. When looking at the sEPB AGR it is evident that growth values for OA6 and OA62 in interval DAP 32–38 indicate that the EMS-induced genetic change led to improved growth parameters compared to the original variety, BARI Gom-25 (Figure 1). In contrast, OA10 and, to some extent, OA23 have gathered mutations that impair growth compared to BARI Gom-25. OA25 and OA52 performed similarly to BARI Gom-25 (Figure 1).

The observed increase and decrease in biomass of mutagenized lines can have several causes, as numerous factors related to both osmotic and ion stress are involved in biomass maintenance under salinity stress [6]. For example, the lower Na^+^ and higher K^+^ accumulation observed in OA6 and OA62 (Figure 3 and Figure 4) indicate the importance of these factors for the observed biomass values, which also lead to a lower Na^+^:K^+^ molar ratio for these lines (Figure 5); all these parameters are beneficial for salinity resistance [6,19,20]. The photosynthetic capacity and the ROS system are both important factors to maintain a good biomass production ([34,35]. However, if they were prone to contribute to the salinity stress in any of the lines are to be evaluated in further studies. Thus, both transcriptional and functional genes related to salt stress, e.g., Na^+^ exclusion and K^+^ accumulation [36,37,38], as well as cell physiological parameters, will be a suitable future approach to better understand the mechanism details for the observed phenotypes.

### 3.2. Increased Biomass of OA6 and OA62 Correlated Well with Efficient Water Use

Analysis of the WUI of the lines of interest revealed that both OA6 and OA62 tended to maintain a higher WUI than BARI Gom-25 (Figure 6). This indicates that these lines managed more efficiently to use their limited water reservoir to maintain growth compared to BARI Gom-25 in the conditions tested. For the other lines, such as OA10, the trend was that WUI remained lower than BARI Gom-25 (Figure 6). Higher biomass (e.g., leaf size) creates a potential for greater transpiration and possibly water use, although there is not necessarily a linear relationship with WUI [39]. However, in our study, increased biomass and WUI were correlated for two of our lines, OA6 and OA62, to a greater extent than the correlation observed for BARI Gom-25 (Figure 1 and Figure 6). Another line (OA10) had low biomass production and low WUI (Figure 1 and Figure 6), indicating that this line lacked tolerance to the osmotic stress. Osmotic stress often causes a deterioration of plant monovalent ion balance [40], but for OA10, no obvious ion imbalance was detected (Figure 2, Figure 3, Figure 4 and Figure 5). Studies suggest that modern hexaploid wheat genotypes have shifted their biomass from below ground to above ground to optimize water use [41], however, this trait was not investigated as part of this study. Moreover, as ABA can prevent transpiration and thereby also decrease Na^+^ movement from the root to the stem [42], it would be a feature to look for in future studies to understand better the patterns that appeared.

### 3.3. Ion Concentration/Homeostasis

#### 3.3.1. OA23 Retained High Na^+^ Levels and Maintained Performance

Regarding Na^+^ content per dry matter, OA23 accumulated more leaf Na^+^ than BARI Gom-25, determined as µmol Na^+^/g-DW (Figure 3). Other OA lines had similar or improved capacity to exclude Na^+^ from the leaf compared to BARI Gom-25. OA6, OA10, and OA62, for example, were better excluders based on their leaf Na^+^ per DW (Figure 3). However, OA23 had more than 3-fold more Na^+^ accumulated following the 120 mM NaCl treatment relative to BARI Gom-25 and still maintained similar sEPB AGR and higher TKW values (Figure 1 and Figure 3, Table 1). This indicates that OA23 could retain Na^+^ in the leaves and still perform relatively well, which is a trait of interest due to the potential to use this feature as part of strategies to remediate salt-affected soils. Thus, the mutagenized lines differed in their Na^+^ accumulation trends relative to BARI Gom-25, with some lower and some higher values.

Although low Na^+^ accumulation in leaves is generally seen as a sign of salt tolerance, several studies, including wheat, clearly show that this is not always the case [37,43]. Bread wheat is recognized to have an excellent Na^+^ exclusion mechanism, and further investment in this traits will likely not give any significantly improved salinity tolerance [37]. Thus, it remains of interest to further study what mechanisms and genes are linked to the observed Na^+^ exclusion of OA6, OA10, and OA62, as they all show improved salinity tolerance. The accumulation of Na^+^ in OA23 suggests that genes associated with osmotic adjustment or tissue tolerance might play a role [37], rather than genes linked to Na^+^ exclusion.

#### 3.3.2. Cl^−^ Levels Were Low in All Mutant Lines Relative to the Control

Excess Cl^−^ ion accumulation can cause toxic effects, as has been shown for *Vicia faba* (faba bean) during saline conditions [44], and plant toxicity thresholds for Cl^−^ have been reported to be similar to thresholds for Na^+^ [20,45]. In the present study, the mutant lines generally had lower leaf Cl^−^ content values than BARI Gom-25 (Figure 4). The difference between the Cl^−^ values of BARI Gom-25 and the mutagenized lines was not as great as the differences observed for Na^+^ values (Figure 4). BARI Gom-25 had higher DW concentrations of Na^+^ than Cl^−^. This pattern was also observed for OA23 and OA25, whereas for OA6, OA10 and OA62 the Cl^−^ values tended to be greater than the values for Na^+^ content, and OA52 did not fit the patterns for these other lines (Figure 4). Thus, in contrast to the trend for Na^+^ values, it seems that the mutations within the lines have contributed to the deviation from BARI Gom-25 only in one direction, lowering the Cl^−^ accumulation in shoot leaves. Further studies could investigate whether this lower level is due to sequestration of Cl^−^ in root tissue, or reduced uptake from the soil [23].

#### 3.3.3. K^+^ Levels Were Generally Higher Than the Control except for OA23

In general, all lines had greater leaf K^+^ content than BARI Gom-25, except OA23, which had a pattern of slightly lower leaf K^+^ content (Figure 2). OA6 and OA62 generally had the highest leaf K^+^ content values. The K^+^ concentration values between salinity levels for each line stayed in a narrow range of circa 100 µmol K^+^/g-DW (Figure 2). Increased leaf K^+^ concentration could be hypothesized due to a increase in the activity of K^+^ transporters, such as *TaHAK1* or *TaHAK11* [46,47]. However, there are newly emerging genes in wheat that could increased K^+^ transport as well [38]. Further in-depth studies at the molecular level, to distinguish between the individual mechanism that most likely occur in the mutagenized lines, is required.

#### 3.3.4. Ion Ratios—Increased K^+^ Levels Manage Na^+^ Accumulation in Three Lines

For plants to grow successfully in salt-affected soils, they must maintain a reasonably low Na^+^:K^+^ ratio in their cytoplasm relative to what is present in the surrounding environment [48]. A low Na^+^:K^+^ ratio is favorable, providing a better chance for K^+^ to outcompete Na^+^ and maintain its vital cellular functions. The NaCl treatments caused the BARI Gom-25 Na^+^:K^+^ ratio to increase. However, OA6 and OA62 retained relatively lower Na^+^:K^+^ ratios following the NaCl treatments (40–160 mM) than BARI Gom-25 (Figure 5). OA10 tended to have reasonably low Na^+^:K^+^ ratios for the milder salt treatments but a higher Na^+^:K^+^ ratio than BARI Gom-25 at 160 mM NaCl (Figure 5). Thus, there was a reasonable capacity for these three lines to manage the Na^+^ accumulation by increasing the K^+^ accumulation compared to the control. In contrast, OA23 was poor at countering Na^+^ accumulation with increased K^+^ accumulation compared to BARI Gom-25 (Figure 5). Despite this, the TKW remained high for OA23, although the growth parameters were not as favorable as those of BARI Gom-25. This observation hypothesizes that the compartmentalization of Na+ might manage high Na+ levels detected in OA23’s shoot into the vacuole.

Nevertheless, further tests are required to investigate whether this line differs in the subcellular partitioning of Na^+^. Na^+^ is believed to substitute for K^+^ in some cellular enzymatic activities, but not all [49]. However, a low Na^+^ and a high K^+^ content are preferred and have previously been reported for salt tolerant genotypes [32], and high K^+^ accumulation reportedly enhances plant growth under salt stress [50]. In addition, to understand genes directly linked to the transport and accumulation of Na^+^ and K^+^ during salinity stress in different compartments and tissues, the control and regulation of salinity tolerance mechanisms by transcription factor families, such as WRKY and DREB, need to be better understood [51,52]. That will help elucidate salinity tolerance mechanisms as they have critical roles in regulating genes linked to ion transport, antioxidant defense, and osmotic pressure [38].

### 3.4. Mutagenized Lines Overall Had Relatively High TKW Relative to the Controls in Saline Conditions

One of the most problematic consequences of salt stress is reduced yield. Yield reduction through increased NaCl is expected. For example, a recent study showed a significant seed weight reduction of ca. 20–40% for five wheat varieties grown at 150 mM NaCl, with one variety showing only 15% seed weight reduction [53]. However, TKW is a different parameter to yield, and in our study, these measures are regarded as two different factors. Some varieties might have high seed weight but fewer seeds and consequently lower yields. Therefore, one could expect that our lines encountered difficulty taking up water and resources at increased salinity and could have had difficulty filling the grains resulting in lower TKW values. Interestingly, this was not observed in our experiment. A possible factor that could have influenced this is the daily watering of pots to field capacity. With a single plant grown per pot and no competition for water or other resources, it is plausible that the roots were capable of supporting the plants with the required resources despite increased salinity.

BARI Gom-25 generally has a TKW of 54–58 g [9]. We obtained a TKW of 51.5 g at 0 mM NaCl in the greenhouse-grown plants for BARI Gom-25 (Table 1). BARI had a TKW reduction of ca. 21% at 120 mM NaCl due to the salinity, whereas OA6 maintained biomass and WUI and only faced a TKW reduction of ca. 12%, indicating that OA6 is likely to be more tolerant to salt stress than BARI Gom-25. Following treatments of 80 mM to 160 mM NaCl, all the mutant lines had a higher TKW than BARI Gom-25, except for OA10, which had a lower seed weight, and OA62 varied with some lower and some higher values in this range (Table 1). OA25 and OA52 had greater TKW values at 120 mM NaCl than any other lines, and the values of ca. 30–40% were greater than BARI Gom-25. However, following the 160 mM NaCl treatment, the differences were less remarkable between mutagenized lines and BARI Gom-25; most lines remained in the range of a 10–15% reduction (Table 1). Thus, our mutagenized lines, except for OA10, tended to perform equally or better than BARI Gom-25 when considering the TKW values.

When plants are under stress in field conditions, several complex interacting factors are involved in the ability of plants to withstand stress. The data reported here are from plants grown under controlled conditions with less complexity in the factors challenging plants’ stress tolerance ability. Although there was no significant difference in TKW at increased salinity in this experiment, this trend might differ under an actual field study or in a study where several plants are grown in a single pot. Hence, to find out if the observed TKW results are correlated to, e.g., yield reduction, another experimental assay with a larger number of plants and a setup simulating plant-to-plant interactions in the field is required.

There is not always a correlation between TKW and biomass; here, we observed that OA23 and OA52 had relatively high TKW values but without performance differences relative to BARI Gom-25 concerning their growth parameters (Figure 1, Table 1). OA10 had a relatively low TKW and low WUI and biomass accumulation compared to BARI Gom-25 (Figure 1 and Figure 6, Table 1). OA62 had relatively high TKW values, high predicted growth values, and high WUI compared to BARI Gom-25 (Figure 1 and Figure 6, Table 1). OA62 did not have as high TKW values as OA6 but remained similar relative to BARI Gom-25 at the higher NaCl treatment levels (Figure 1 and Figure 6, Table 1).

### 3.5. Variation in the Improved Salinity Tolerance-Associated Variables Observed in the Characterized Lines

OA6 performed better than BARI Gom-25 concerning all measured parameters. These included improved biomass accumulation, water use, ion content trends, and predicted yield, and OA6 was considered to be likely to have an improved salt exclusion mechanism and greater tissue tolerance than BARI Gom-25 (Table 2). OA62 was similar to OA6 for some parameters but had slightly poorer performance, where the TKW did not outperform BARI Gom-25. However, this line may have an improved salt exclusion mechanism and tissue tolerance (Table 2). OA62 was the only line selected in this study with a 2-fold higher total grain weight than the control in a previous study (OA62 then designated the name OA120) performed at the Salt Farm Texel [12]. The difference from the observed TKW can be due to fewer plants used in our study (three versus 96), different environments (indoor versus outdoor), and that salt was applied two weeks earlier in our study [12]. OA10 had favorable ion accumulation trends but low biomass, low water use efficiency, and low yield. Thus, it may have limited osmotic tolerance, which cannot be compensated with improved ion accumulation values (Table 2). However, it might be worth further investigating OA10’s performance at 160 mM NaCl or higher levels. OA10 accumulated a very high level of Na^+^ in the 160 mM NaCl treatment with a seemingly limited penalty in other parameters; this indicates OA10 could be of interest for discovering functional mechanisms for remediating saline soils. OA23 Na^+^ accumulation was also relatively high, and in contrast to OA10, the OA23 line had a relatively good predicted yield despite not having improved WUI (Table 2). OA25 and OA52 had the most improved predicted yield capacity and were similar in other measured parameters to BARI Gom-25 (Table 2). These lines had only subtle differences in biomass and ion distribution trends relative to the more substantial differences between the other mutant lines and BARI Gom-25. Overall, the mutant lines are promising tools for further investigating parameters that may be useful for improving wheat performance in salt-affected soils and wheat productivity in general. Moreover, by sequencing candidate genes responsible for the observed phenotypes we expect to identify SNPs in each line that can be used for gene editing to improve salt tolerance in other wheat cultivars or crops with homologues genes.

## 4. Materials and Methods

### 4.1. Plant Material

The wheat variety, named BARI Gom-25, used as a control, was developed by the Bangladesh Agricultural Research Institute (BARI). The samples comprised 35 mutagenized wheat lines derived from BARI Gom-25 using ethylene methanesulfonate (EMS) mutagenesis and designated OA1–35 [7]. Seeds (M6 or M7 generation) sent from Sweden for the experiments were propagated for one generation for release from biosecurity at the Australian Plant Phenomics Facility, University of Adelaide, Australia. The following M7 and M8 generation seeds were used for the phenotypic studies.

### 4.2. Experiment Description

The experiment comprised 36 wheat lines (wild-type BARI Gom-25 and 35 mutagenized lines), five salinity treatments (0 mM, 40 mM, 80 mM, 120 mM, and 160 mM NaCl in the soil solution), and six replicates, making a total of 1080 plants. The plants were grown in two adjacent greenhouses fitted with conveyors (Smart houses). Each replicates occupied a block of eight consecutive conveyor lanes in a single Smarthouse, three blocks each in the South West (SW) Smarthouse and the South East (SE) Smarthouse. A split-unit design was used to randomize the salinity treatments to clusters of 36 carts and the lines to the carts within a cluster. The randomizations were performed using dae [54], a package for the R statistical computing environment [55]; then, the randomization of lines to carts was optimized using the R package od [56]. Planting occurred on the 4 July 2019, denoted Day After Planting 0 (DAP 0).

Seeds were potted in 150 mm diameter × 195 mm height pots (Berry Plastics Corporation, Evansville, IN, USA) with drainage holes. Each pot contained 2.6 kg soil mixture; (50% (*v*/*v*) University of California mix, 35% (*v*/*v*) peat mix, 15% (*v*/*v*) clay loam soil from Angle Vale, South Australia, Australia. Pots were placed on benches at the back of the Smarthouse for 14 days before being transferred to the conveyor system of the Smarthouse for imaging. While on the conveyor system, pots were placed into deep saucers within individual carriers and watered to weight daily to maintain field capacity at a gravimetric water content of 17% (g/g). The soil surface was covered with blue rubber mats that allowed water to permeate while reducing evaporation from the soil surface. On DAP 18, 200 mL saline solution was applied to the saucer, temporarily increasing the water content above the field capacity of the pot. This was to avoid a sudden shock from the salinity treatment. Over the following days, pots were allowed to dry down to field capacity, by which time the respective NaCl concentrations in the soil solution were reached, as described previously [57]. Plants in the SW Smarthouse were imaged for 25 days, and plants in the SW SE Smarthouse for 26 days. At the end of the imaging period, the three replicates in the SW Smarthouse were discarded, while the three replicates in the SE Smarthouse were grown to maturity for seed harvest. Greenhouse conditions were set to a day temperature of 23 °C and a night temperature of 18 °C, and the average light intensity level at midday was 400 µmol m^−2^ s^−1^.

Imaging was carried out daily from DAP 13 to DAP 38 inclusive, except DAP 18, when the salt treatments were applied. During the post-imaging period (DAP 39–99), the plants in the SE Smarthouse remained in place until harvest and continued to be watered daily. During the imaging period, Red Green Blue (RGB) images were taken from multiple angles to determine the plants’ projected shoot area (PSA) of the plant. The PSA (kpixels) was defined as the sum of the areas measured from three camera views, comprising two side views at an angular separation of 80 degrees and a view from above.

### 4.3. Preparation of Image Data

The PSA was converted to estimated plant biomass (EPB g) by dividing it by a conversion factor of 175. The conversion factor is the slope from a linear regression fitted to data for the smoothed PSA values (kpixels) for DAP 30 and post-imaging dry weight (g) from another wheat experiment run in the Plant Accelerator that had 149 lines and two salinities (0 mM, 150 mM NaCl). The EPB was processed using the Smoothing and Extraction of Traits (SET) method [58]. The absolute growth rate of the EPB (EPB AGR) and the relative growth rate (EPB RGR) were calculated by differencing consecutive EPB and logarithmic (ln) EPB (lnEPB) values for each plant, respectively, and dividing by the time differences.

Several approaches to smoothing the data were compared, aided by the exploratory routine probeSmoothing from the R package growthPheno [59]. It was decided to smooth the dataset on the logarithmic EPB scale with six degrees of freedom (df = 6, mild smoothing) to produce smoothed EPB values (sEPB). The sEPB AGR was calculated from the sEPB as described for the EPB (Figures S44–S46).

After examining plots of the smoothed data, five plants from the three lowest salinity levels that experienced negative growth in the last six days or more of imaging were excluded from further analysis. The resulting analysis dataset had 1075 individual plants.

To derive traits for the analysis, the imaging period was divided into intervals defined by the time-points DAP 19, 23, 28, 32, and 38. This choice of endpoints was guided principally by qualitative patterns in the sEPB AGR. In particular, it was assessed that, while some plants continued to increase throughout the time of imaging, the typical behavior of the remaining plants was as follows: DAP 19–23 was a period of increasing growth immediately post-treatment; DAP 23–28 corresponded to near-constant growth; DAP 28–32 corresponded to slowing growth, and DAP 32–38 was characterized by considerable variability between plants. Thus, a total of 13 image traits were selected for measurements; single-day responses; sEPB for DAP 19, 23, 28, 32, and 38; and interval responses: sEPB AGR for each of the DAP intervals 19–23, 23–28, 28–32 and 32–38. Each of these traits was computed using the R package growthPheno [59], yielding a single value for each plant for each trait. After imaging, leaf samples were taken from all plants for biochemical analysis.

### 4.4. Water Use

For the imaging period of 25 days, the volume of water in each pot was programmed to maintain 392 g of water, equal to a 17% (g/g) water content. Daily water use was computed (WU mL) and smoothed (sWU mL) for each plant during the imaging period (DAP 13–38), based on the net change in pot weight between successive days. No attempt was made to differentiate between biological consumption and evaporative loss. The water use index (sWUI g mL^−1^) was then defined as the ratio of the daily change in sEPB to the daily WU (higher sWUI values correspond to higher water efficiency). In addition, total water use was computed over the entire imaging period (DAP 13–38) for all plants. Water use traits were measured as interval responses for sWUI for each DAP interval 19–23, 23–28, 28–32, and 32–38.

### 4.5. Harvest (Leaf) Data

The leaf analysis dataset comprised *n* = 1071 plants after removing five EPB outliers (negative growth in the last six days) and four otherwise healthy plants whose leaf samples were found to have senesced. A total of nine harvest leaf traits were measured; sodium dry-matter concentration (Na-DW, μmol/g-DW), potassium dry-matter concentration (K-DW, μmol/g-DW), chloride dry-matter concentration (Cl-DW, μmol/g-DW), Na^+^:K^+^ molar ratio, Na^+^:Cl^−^ molar ratio, and K^+^:Cl^−^ molar ratio.

### 4.6. Measurement of Leaf Ion Concentration

The fifth leaf on the main tiller was harvested at DAP 39 (SW Smarthouse) and DAP 40 (SE Smarthouse), corresponding to 21 and 22 days after salt treatment. The fresh weight of the leaf tissue was measured immediately after harvest, and leaf samples were dried in an oven until a constant weight was reached. The dry weight was recorded, and the dried leaf tissue was placed in a 50 mL conical tube and incubated with 10 mL of 1% (*v*/*v*) HNO_3_ at 80°C in a HotBlock (Environmental Express, Mount Pleasant, SC, USA) until the tissue turned translucent. The tube was shaken regularly to ensure complete digestion of the leaf tissue. A flame photometer (model 420; Sherwood Scientific Ltd., Cambridge, UK) was used for measuring the K^+^ and Na^+^ concentration, and a chloride analyzer (Model 926 Sherwood) was used for Cl^−^ measurements.

### 4.7. Thousand Kernel Weight (TKW)

At the end of the imaging period DAP 38, three replicates were kept for growth until maturity. All spikes from these plants were harvested at DAP 99. Spikes were threshed, and seeds were counted using a SLY-C Automatic Seed Counter. The seed count of three replicates determined the TKW of each line and control. The differences in the TKW means were analyzed using Minitab 20 statistical software (©2021 Minitab, LLC (State College, PA, USA)). A one-way ANOVA using Welch’s test was employed to evaluate the differences between lines and salinity levels. Games–Howell pairwise comparison was used to group the differences and plot data at a 95% confidence interval. Individual standard deviations were used to estimate the confidence intervals.

### 4.8. Statistical Analyses

A mixed-model analysis was performed for each trait using the R package ASReml-R [60] and asremlPlus [61], packages for the R statistical computing environment. The model included terms for (a) curved trends in the east–west direction that differ between Smarthouses, (b) main effects for Lines and Salinity, (c) the interaction of Lines and Salinity, (d) variation between Blocks of eight consecutive Lanes each, and (e) variation between Clusters within Blocks. The residual effects were assumed to be normally distributed with different variances for each Salinity.

For each trait, a residual likelihood ratio test was performed to determine whether the differential variance was statistically significant. This test returned a positive result for most traits, including all sEPB and sEPB AGR traits. Residual-versus-fitted values plots and normal probability plots of the residuals were inspected to check that the modelling assumptions were met. All plots were found to be satisfactory, except for departures from normality evident for harvest traits. Application of a square-root transformation to the two chloride traits (Cl-DW, Cl-aq), and a logarithmic transformation to the other seven traits (Na, K, and molar ratios) resulted in satisfactory plots. There remained several outliers, which were investigated and retained in the analysis because there was no evidence of the values being erroneous.

For each trait, a Wald F-test with α=0.05 was conducted for the interaction of Lines with Salinity. If this was not statistically significant, similar tests were then performed for the main effects of Salinity and Line. Using the fitted maximal model for a trait, predictions were obtained for each of the lines under both the control and salted treatments.

### 4.9. Rationale behind Selection of Lines for in Depth Studies

We compared the different lines with each other regarding their individual performance versus the control BARI Gom-25 for each parameter. Each line was given a mark for their overall performance versus BARI Gom-25, i.e., either being better or not for each parameter, including all salt concentrations used (Supplementary Figures S1–S41). The preliminary overview was used to select lines for in depth studies of their individual results; good overall parameter values (OA6, OA62), good ability to accumulate Na and still perform well (OA23), good water use (OA10, OA23), and good 1000 seed weight (OA25, OA52). In total, six different lines.

## 5. Conclusions

The mutant lines analyzed in this study had previously been shown to have improved germination in saline conditions relative to BARI Gom-25. We studied whether the lines also differ in salt tolerance parameters later in development and observed that they do differ. However, they are not identical concerning the specific parameters in which they differ. We observed that these lines with improved germination in saline conditions all had different physiological responses to salt later in development. The lines tended to exhibit favorable phenotypes associated with salt-stress tolerance at the ionic phase of salt-stress, such as improved biomass accumulation and exclusion of Na^+^. Identifying the genes linked to the phenotypes observed in this study remains a challenge to resolve in the future. Molecular, biochemical, and transcriptional analyses to explore candidate genes will be the next step toward testing for up- and down-regulated genes and gene families along these lines. The physiological information reported here for the mutagenized lines can be used to devise strategies to verify the relative usefulness of this germplasm as a source of novel salt tolerance traits for improving wheat productivity in saline soils.

## Figures and Tables

**Table 1:** Thousand kernel weight in grams after growth on different salt concentrations. The end of the table gives grouping information of the lines using the Games-Howell method and 95% confidence, *n* = 15. The means that are not sharing the same letter are significantly different.

NaCl /Line	BARI	OA6	OA10	OA23	OA25	OA52	OA62
0 mM	51.65 ± 0.85	53.82 ± 4.40	29.50 ± 1.29	51.84 ± 3.45	54.54 ± 1.89	52.79 ± 0.90	46.48 ± 3.62
40 mM	47.25 ± 2.41	45.82 ± 8.66	26.27 ± 2.43	57.98 ± 7.94	51.90 ± 5.06	46.56 ± 4.51	44.75 ± 0.90
80 mM	48.60 ± 5.28	49.73 ± 12.6	28.76 ± 2.40	62.43 ± 5.58	50.21 ± 5.55	50.43 ± 5.91	44.05 ± 2.42
120 mM	40.81 ± 3.41	47.74 ± 2.93	26.15 ± 1.51	51.13 ± 4.68	58.11 ± 0.19	58.26 ± 21.3	41.57 ± 2.10
160 mM	42.36 ± 4.11	46.76 ± 2.82	25.86 ± 2.46	55.42 ± 8.03	47.20 ± 5.06	42.87 ± 3.97	40.89 ± 1.24
**Mean**	46.13	48.77	27.31	55.76	52.39	50.18	43.55
**Group**	B	AB	C	A	A	AB	B

**Table 2:** The overall pattern of parameters measured. Green, overall higher values than BARI Gom-25; orange, overall lower values than BARI Gom-25.

Parameter/Line	OA6	OA10	OA23	OA25	OA52	OA62
**sEPB AGR**						
**K^+^**						
**Na^+^**						
**Cl^−^**						
**Na^+^:K^+^**						
**sWUI**						
**TKW**						

## Data Availability

The data that support the findings of this study are available from the corresponding author upon reasonable request.

## Supplementary Materials

The supporting information can be downloaded at: https://www.mdpi.com/article/10.3390/ijms231911386/s1.
