# Supplementary material for: Improved Salinity Tolerance-Associated Variables Observed in EMS Mutagenized Wheat Lines
Source: Int J Mol Sci. 2022 Sep 27;23(19):11386. doi: 10.3390/ijms231911386 (PMC9570438; doi:10.3390/ijms231911386)
Supplement: Supplementary file 1 [file ijms-23-11386-s001.zip › ijms-1858100-supplementary.pdf]

# **Supplementary information**, Table S1 and Figures S1 - S46

**Supplementary Table S1.** The *p*-values of the Wald *F*-statistics for the treatment terms. The asterisk (*) indicates that a term is significant at the 0.05 level of significance ($p \leq 0.05$); na = not applicable.

| | Term | | |
|---|---|---|---|
| Trait | Salinity:Line | Salinity | Line |
| sEPB 19 | 0.615 | 0.393 | * <0.001 |
| sEPB 23 | 0.603 | * 0.001 | * <0.001 |
| sEPB 28 | 0.686 | * <0.001 | * <0.001 |
| sEPB 32 | 0.476 | * <0.001 | * <0.001 |
| sEPB 38 | * 0.015 | na | na |
| sEPB AGR 19-23 | 0.264 | * <0.001 | * <0.001 |
| sEPB AGR 23-28 | 0.583 | * <0.001 | * <0.001 |
| sEPB AGR 28-32 | * 0.048 | na | na |
| sEPB AGR 32-38 | * <0.001 | na | na |
| sEPB RGR 19-23 | 0.111 | * <0.001 | * <0.001 |
| sEPB RGR 23-28 | 0.576 | * <0.001 | * <0.001 |
| sEPB RGR 28-32 | 0.247 | * <0.001 | * <0.001 |
| sEPB RGR 32-38 | 0.050 | * <0.001 | * <0.001 |
| Na (DW) | * <0.001 | na | na |
| Na (aqueous) | * <0.001 | na | na |
| K (DW) | 0.051 | * <0.001 | * <0.001 |
| K (aqueous) | 0.150 | * <0.001 | * <0.001 |
| Cl (DW) | * <0.001 | na | na |
| Cl (aqueous) | * <0.001 | na | na |
| Na:K | * <0.001 | na | na |
| Na:Cl | * <0.001 | na | na |
| K:Cl | * <0.001 | na | na |
| sWU 19-23 | 0.095 | * <0.001 | * <0.001 |
| sWU 23-28 | * 0.005 | na | na |
| sWU 28-32 | * 0.002 | na | na |
| sWU 32-38 | * <0.001 | na | na |
| sWUI 19-23 | 0.410 | * <0.001 | * <0.001 |
| sWUI 23-28 | 0.816 | * <0.001 | * <0.001 |
| sWUI 28-32 | 0.227 | * <0.001 | * <0.001 |
| sWUI 32-38 | 0.067 | * <0.001 | * <0.001 |
| WU 13-38 | * <0.001 | na | na |
| WU 13-99 | * <0.001 | na | na |

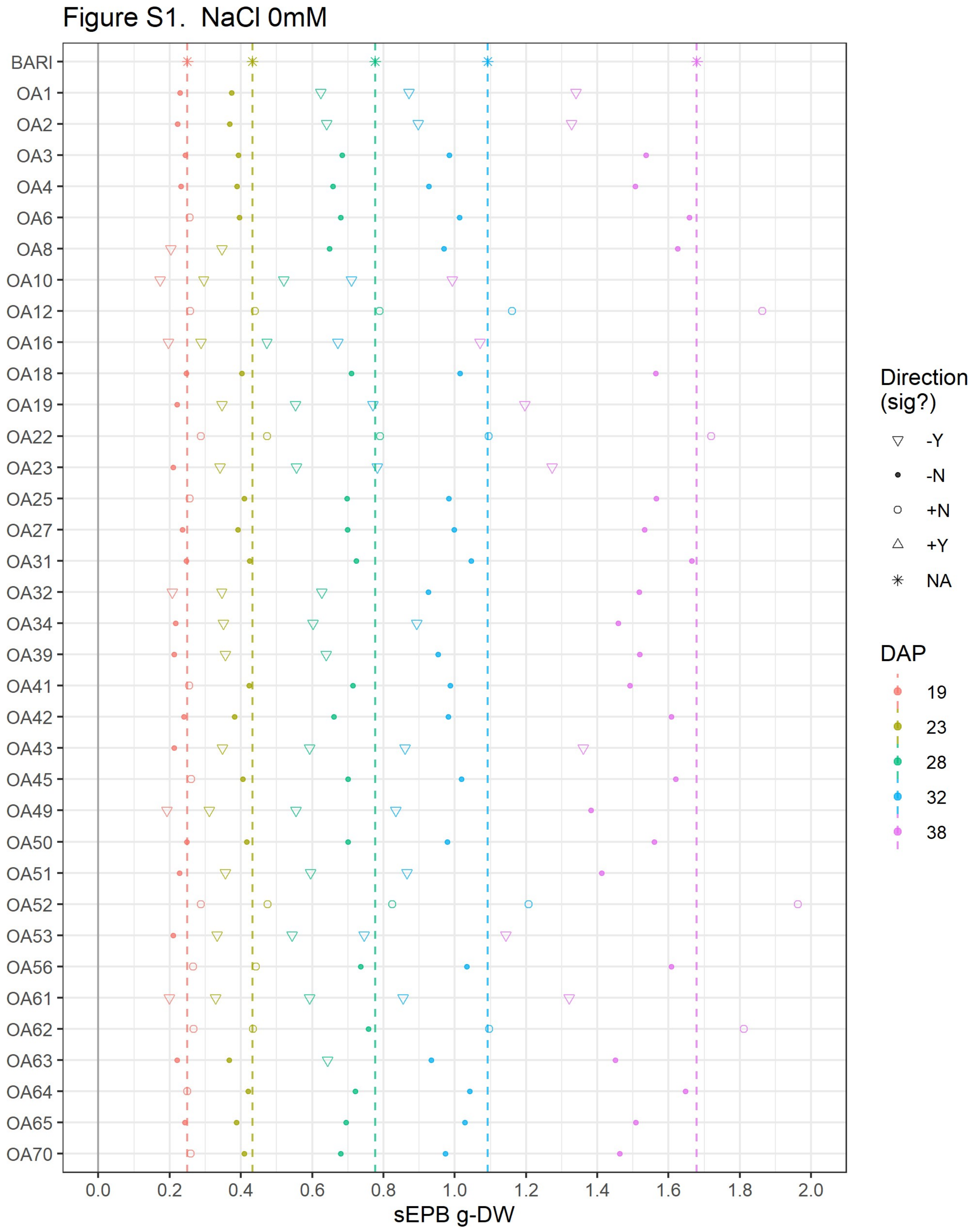
Figure S1. NaCl 0mM
BARI
OA1
OA2
OA3
OA4
OA6
OA8
OA10
OA12
OA16
OA18
OA19
OA22
OA23
OA25
OA27
OA31
OA32
OA34
OA39
OA41
OA42
OA43
OA45
OA49
OA50
OA51
OA52
OA53
OA56
OA61
OA62
OA63
OA64
OA65
OA70
0.0
0.2
0.4
0.6
0.8
1.0
1.2
1.4
1.6
1.8
2.0
sEPB g-DW
Direction (sig?)
-Y
-N
+N
+Y
NA
DAP
19
23
28
32
38

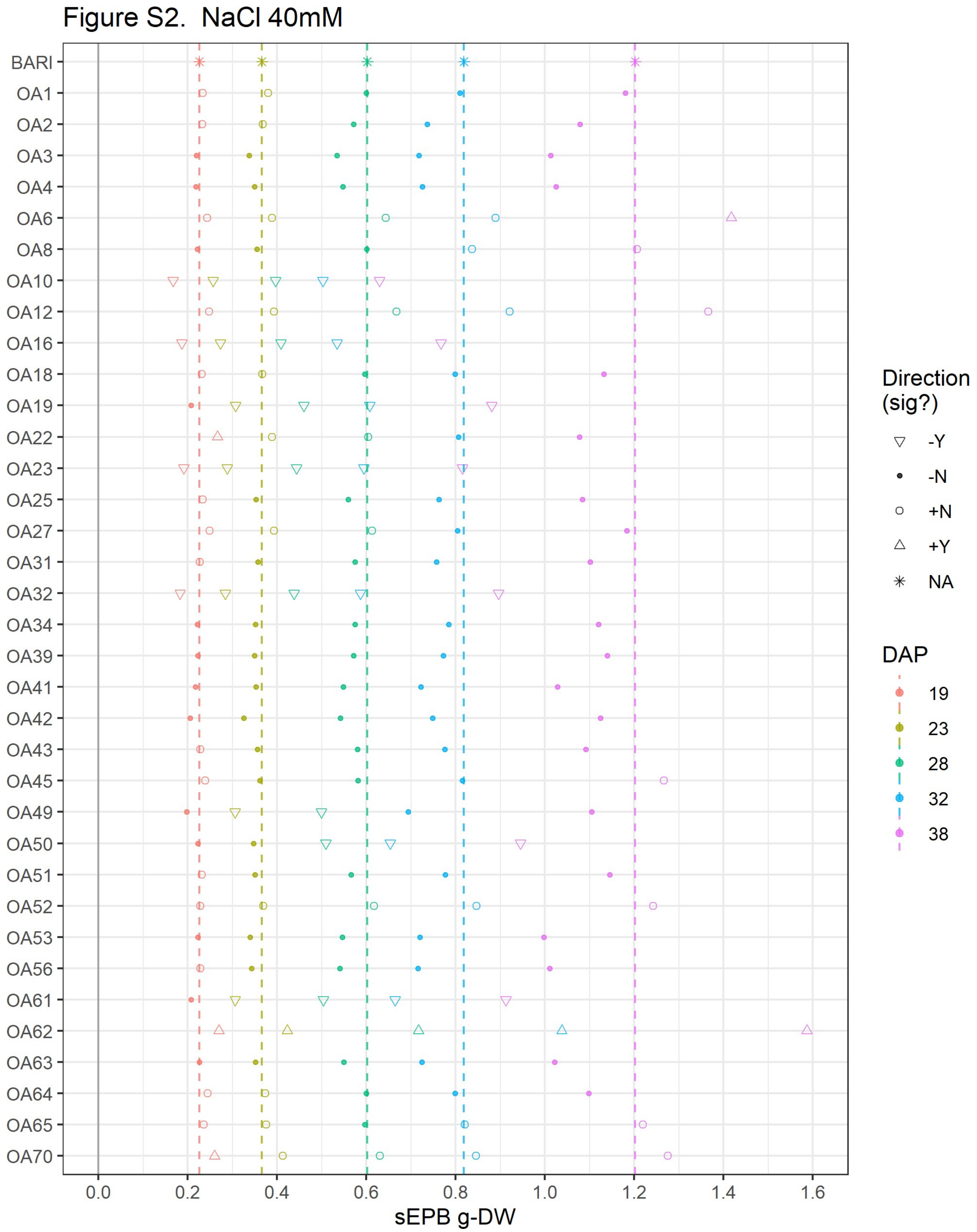

Figure S2. NaCl 40mM
BARI
OA1
OA2
OA3
OA4
OA6
OA8
OA10
OA12
OA16
OA18
OA19
OA22
OA23
OA25
OA27
OA31
OA32
OA34
OA39
OA41
OA42
OA43
OA45
OA49
OA50
OA51
OA52
OA53
OA56
OA61
OA62
OA63
OA64
OA65
OA70
0.0
0.2
0.4
0.6
0.8
1.0
1.2
1.4
1.6
sEPB g-DW
Direction (sig?)
-Y
-N
+N
+Y
NA
DAP
19
23
28
32
38

Figure S3. NaCl 80mM

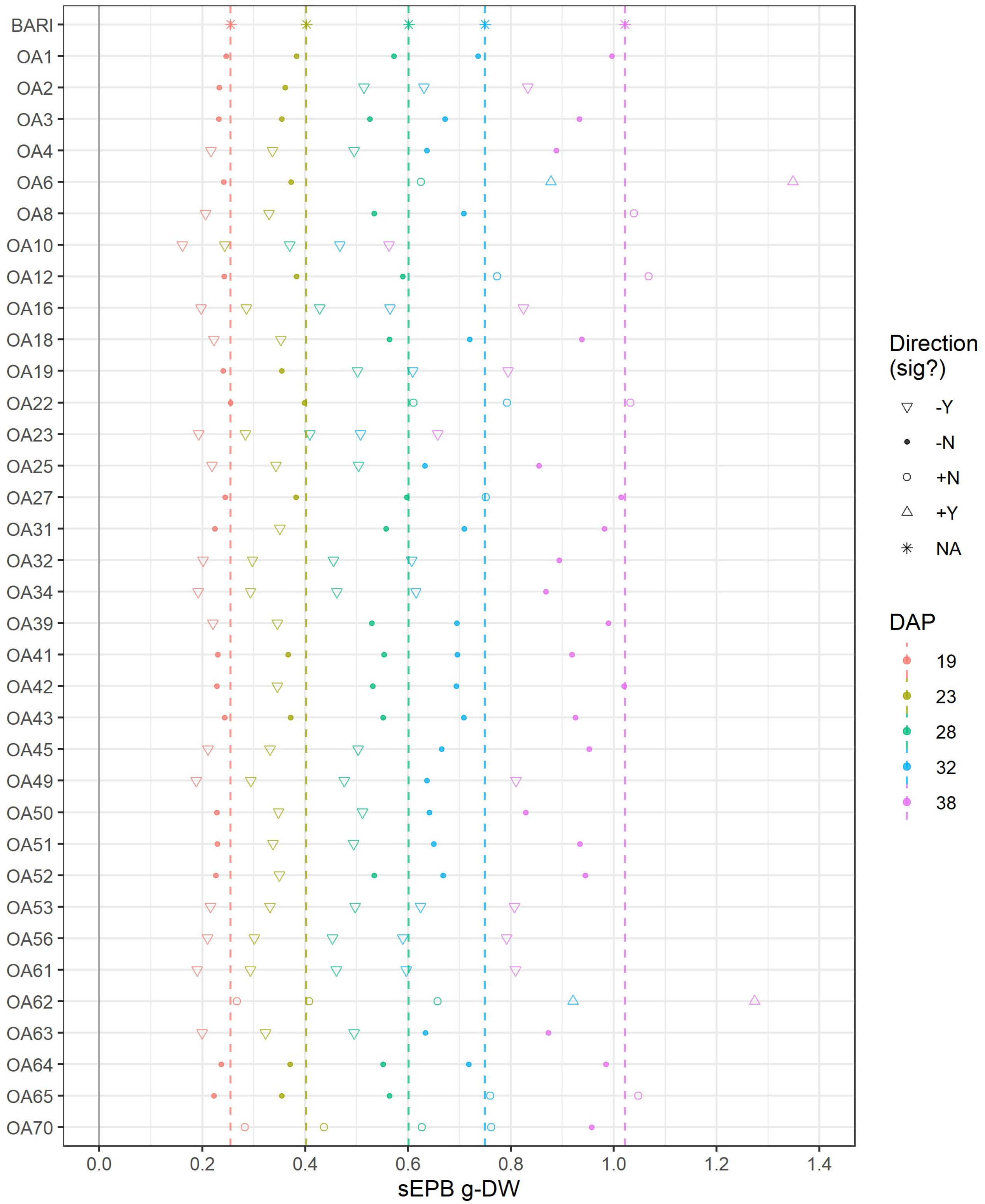

Figure S4.  NaCl 120mM

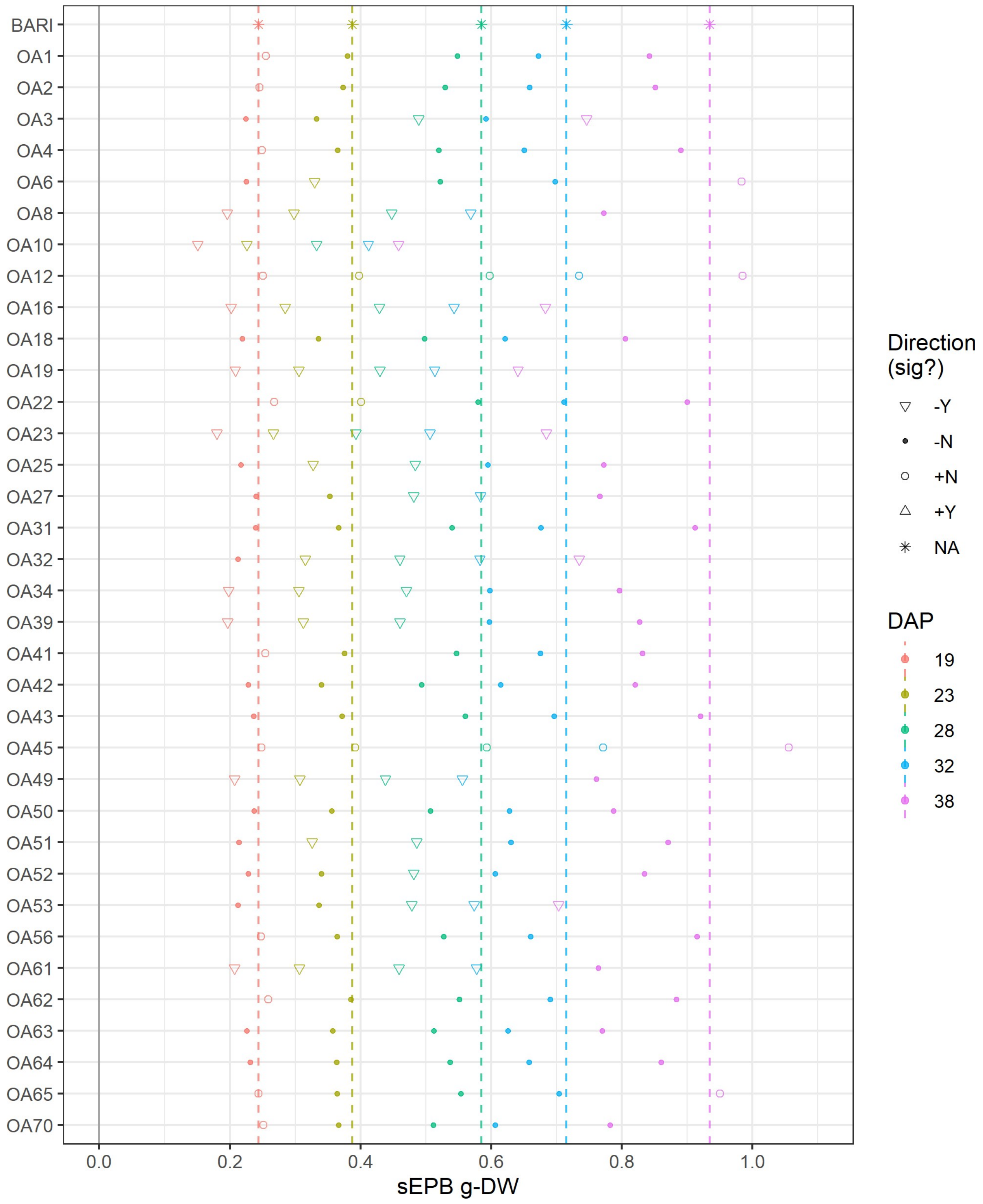

Figure S5.  NaCl 160mM

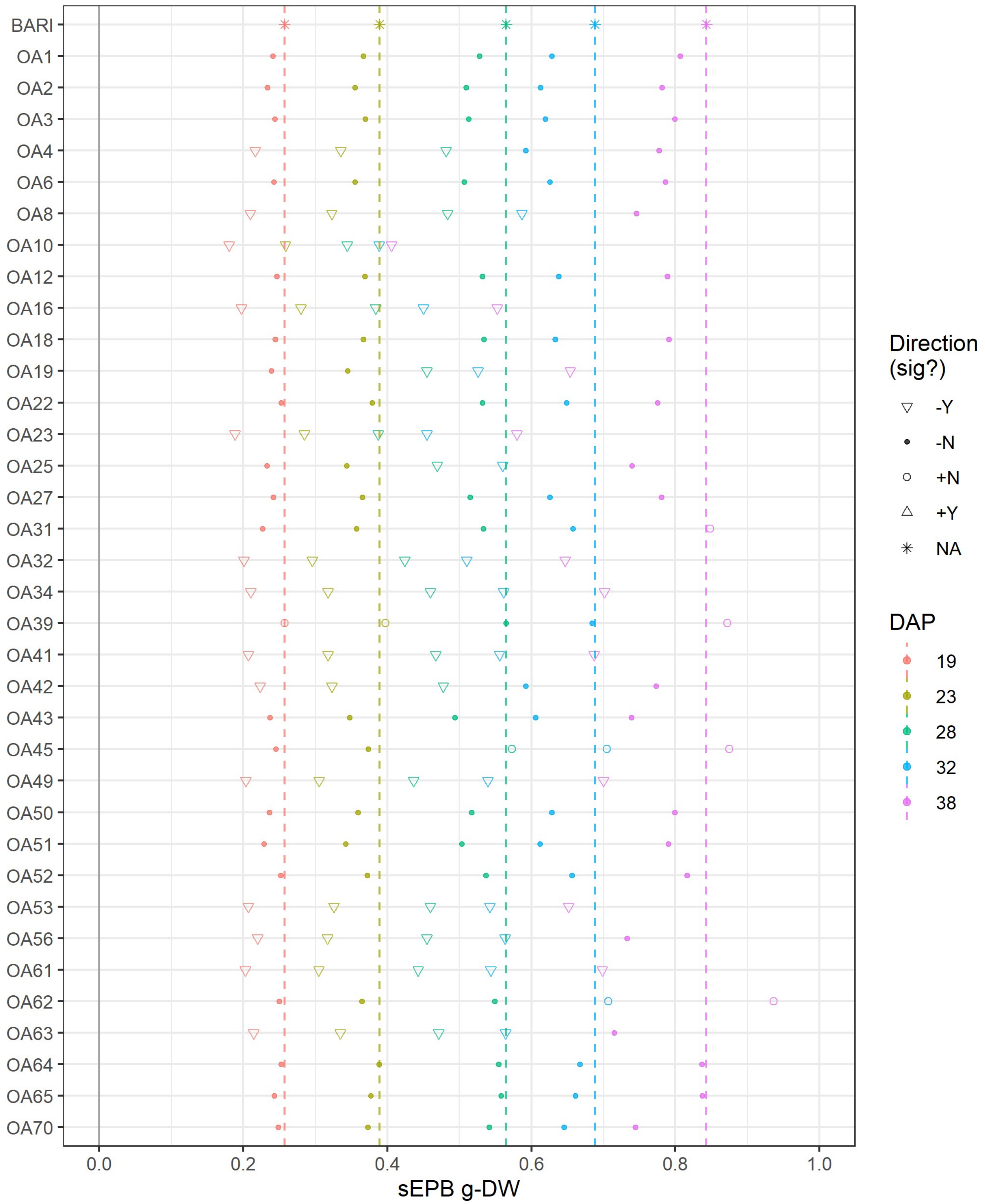

Figure S6.

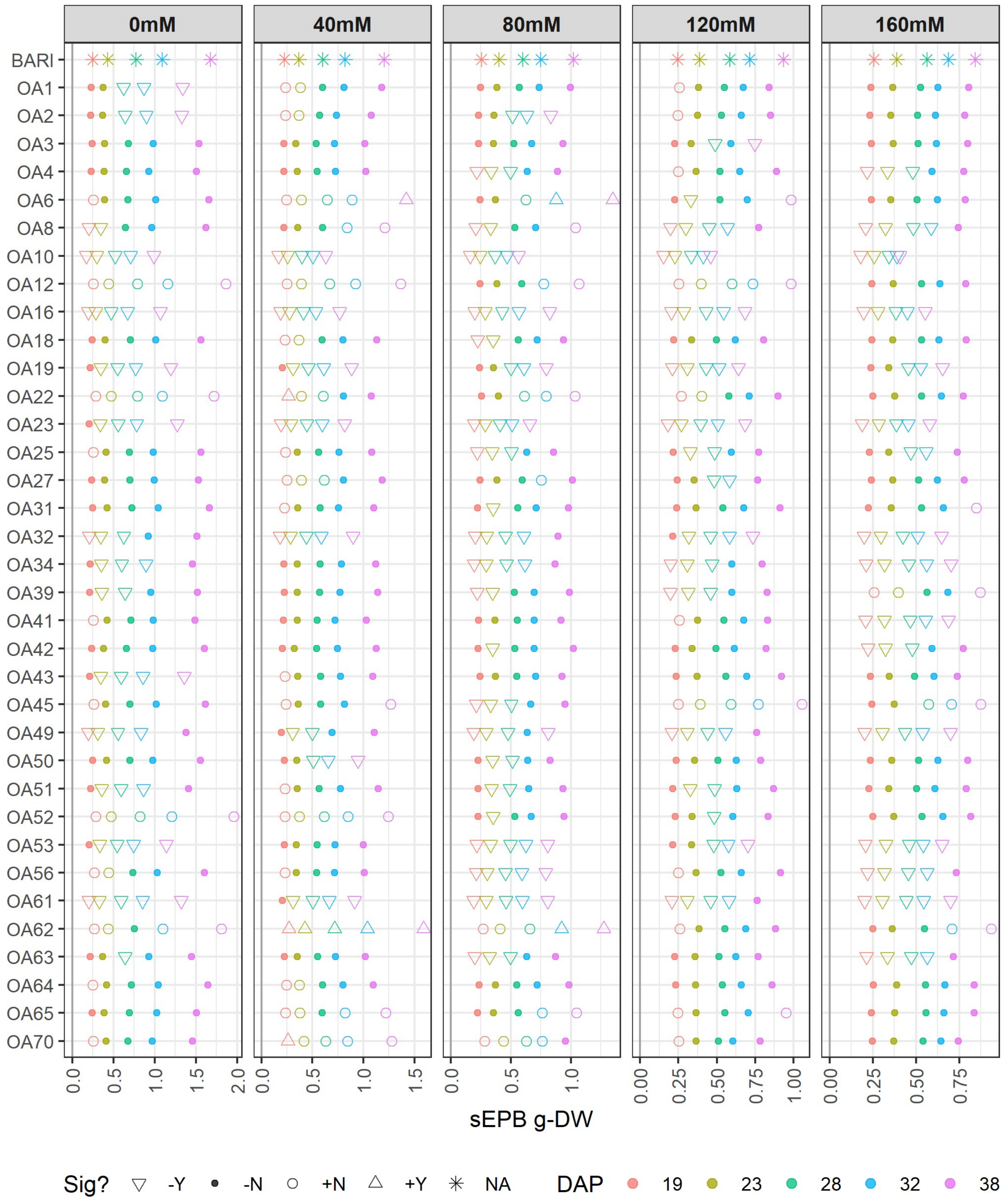

0mM
40mM
80mM
120mM
160mM
BARI
OA1
OA2
OA3
OA4
OA6
OA8
OA10
OA12
OA16
OA18
OA19
OA22
OA23
OA25
OA27
OA31
OA32
OA34
OA39
OA41
OA42
OA43
OA45
OA49
OA50
OA51
OA52
OA53
OA56
OA61
OA62
OA63
OA64
OA65
OA70
0.0 0.5 1.0 1.5 2.0
0.0 0.5 1.0 1.5
0.0 0.5 1.0
0.00 0.25 0.50 0.75 1.00
0.00 0.25 0.50 0.75
sEPB g-DW
Sig? -Y -N +N +Y NA
DAP 19 23 28 32 38

Figure S7. NaCl 0mM

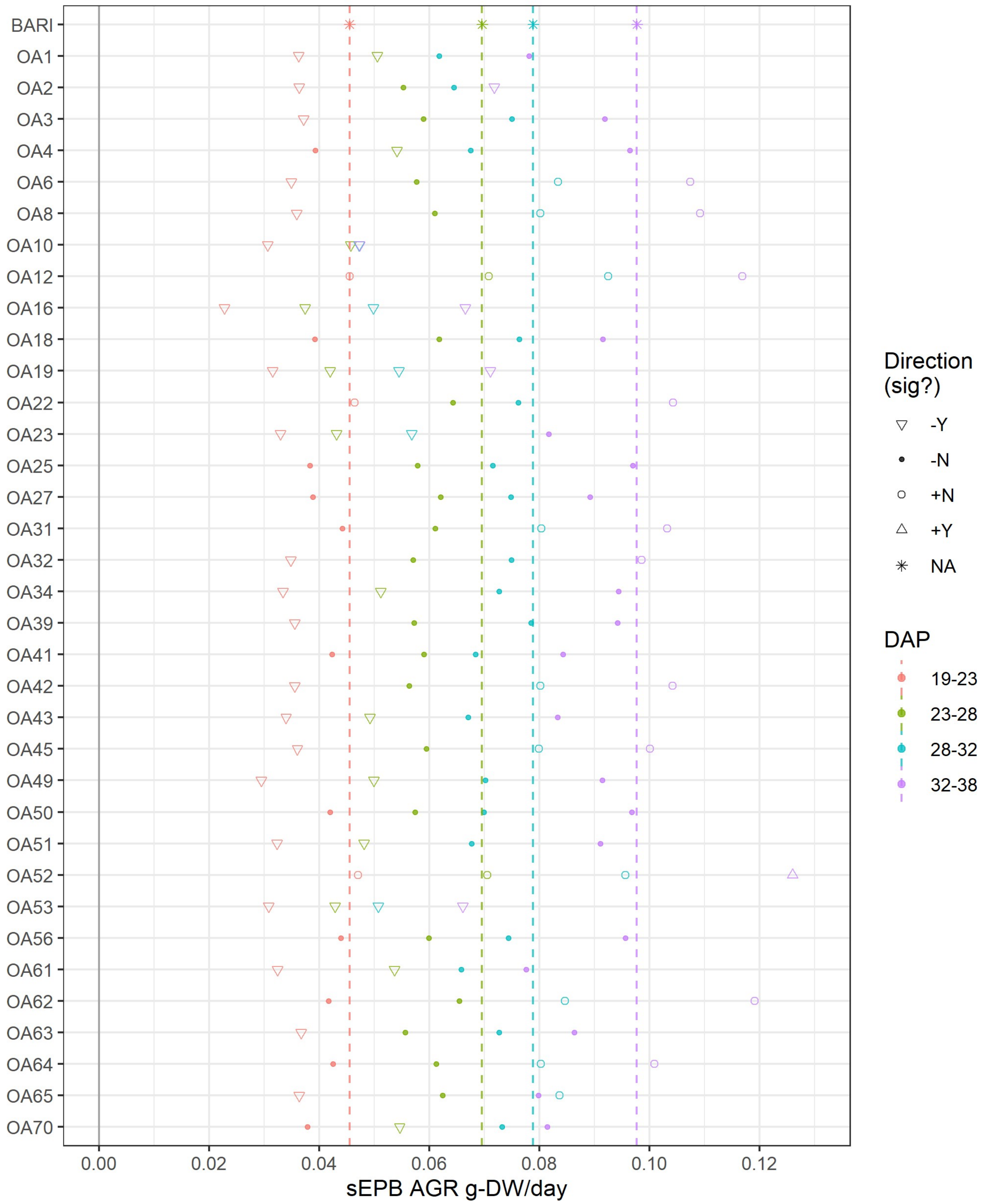

Figure S8.  NaCl 40mM

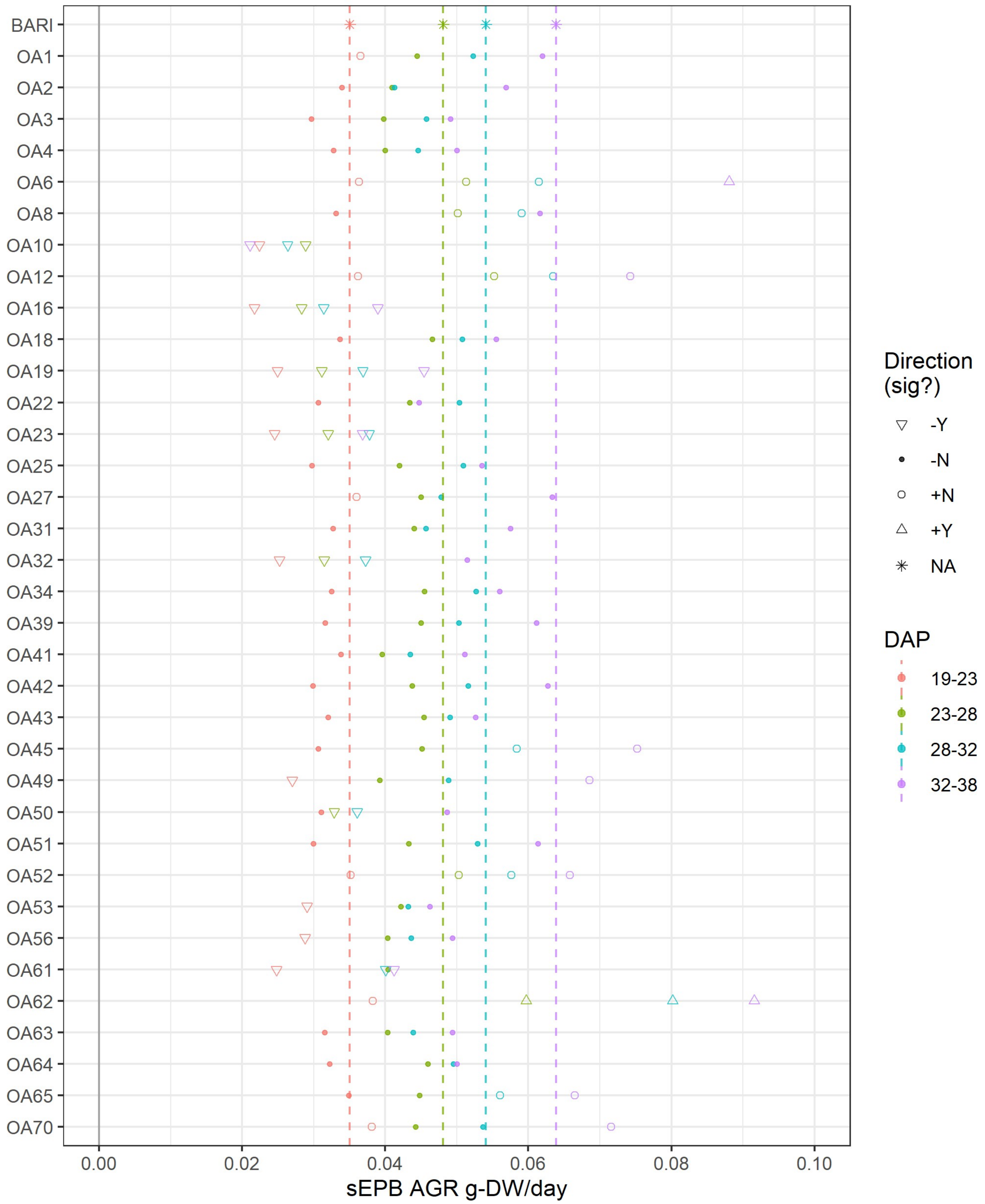

Figure S9. NaCl 80mM

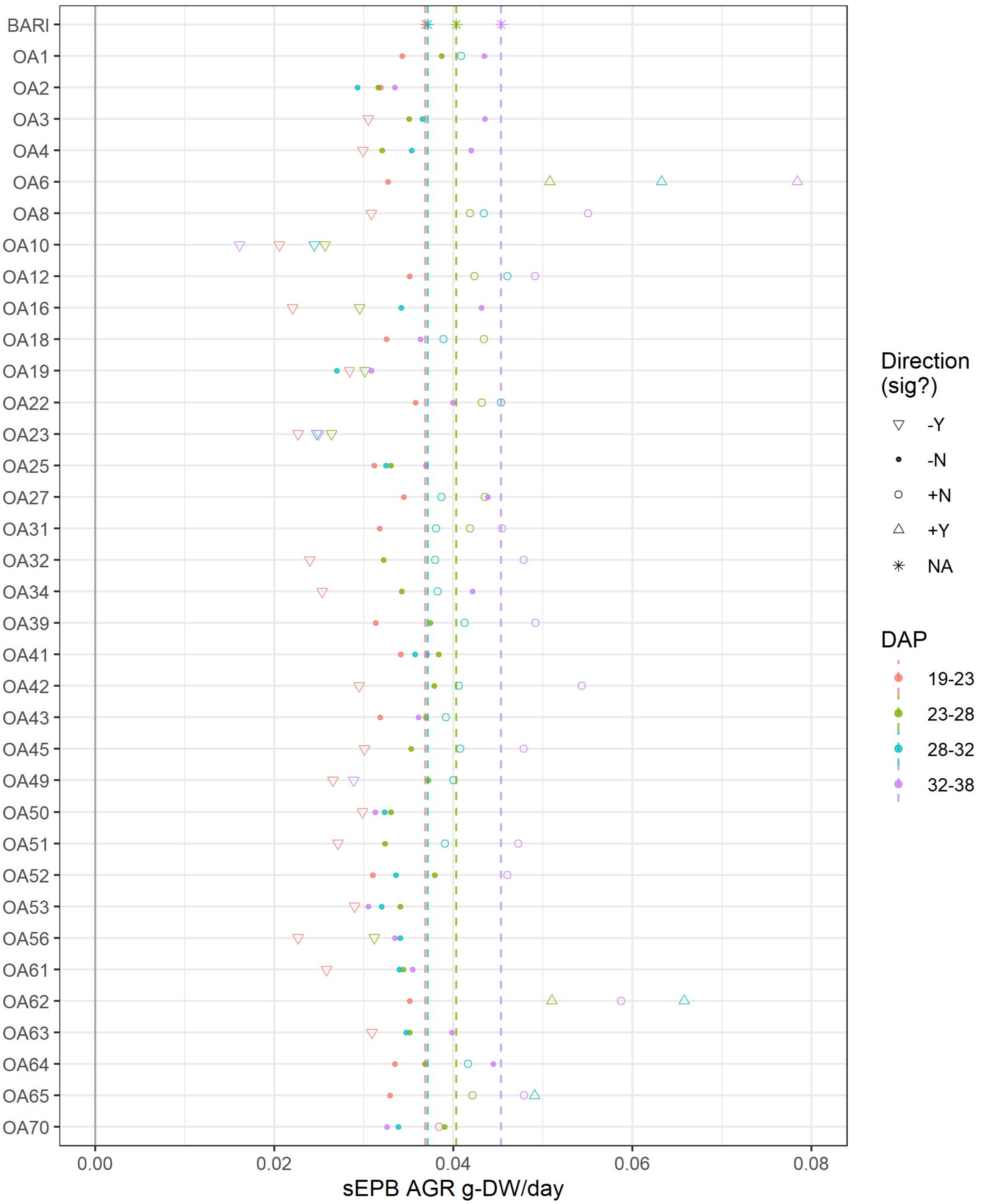

Figure S10.  NaCl 120mM

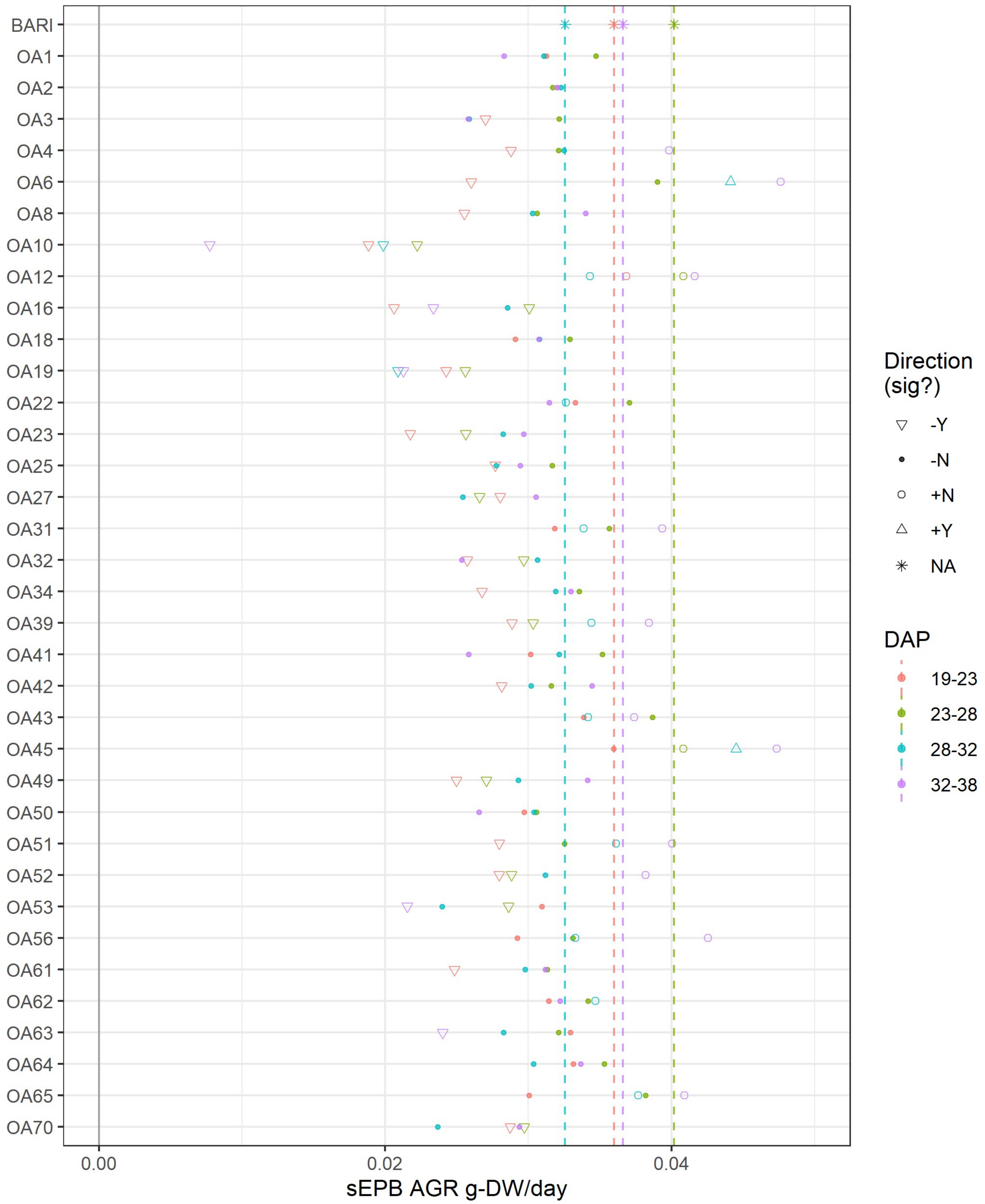

Figure S11.  NaCl 160mM

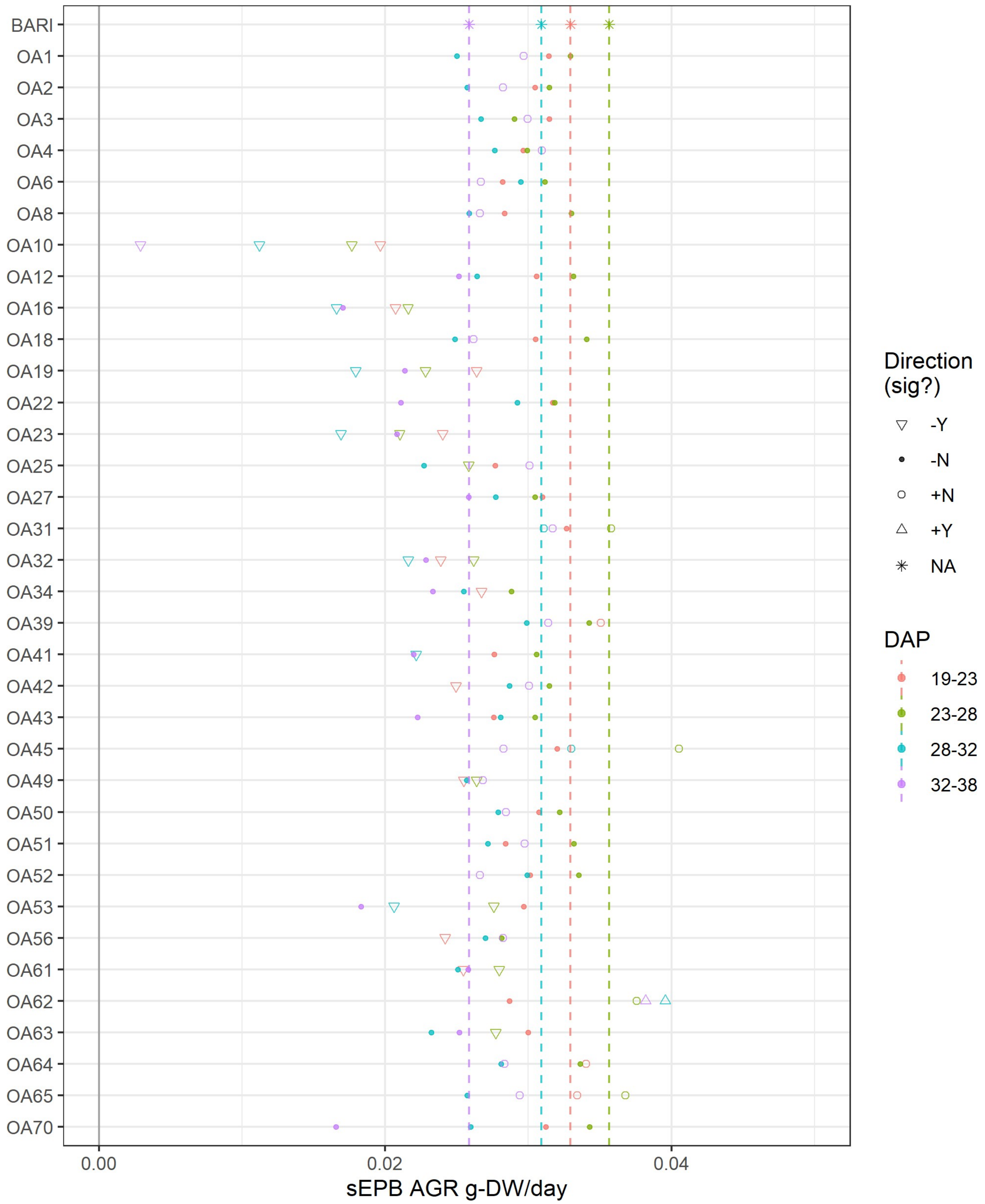

Figure S12.

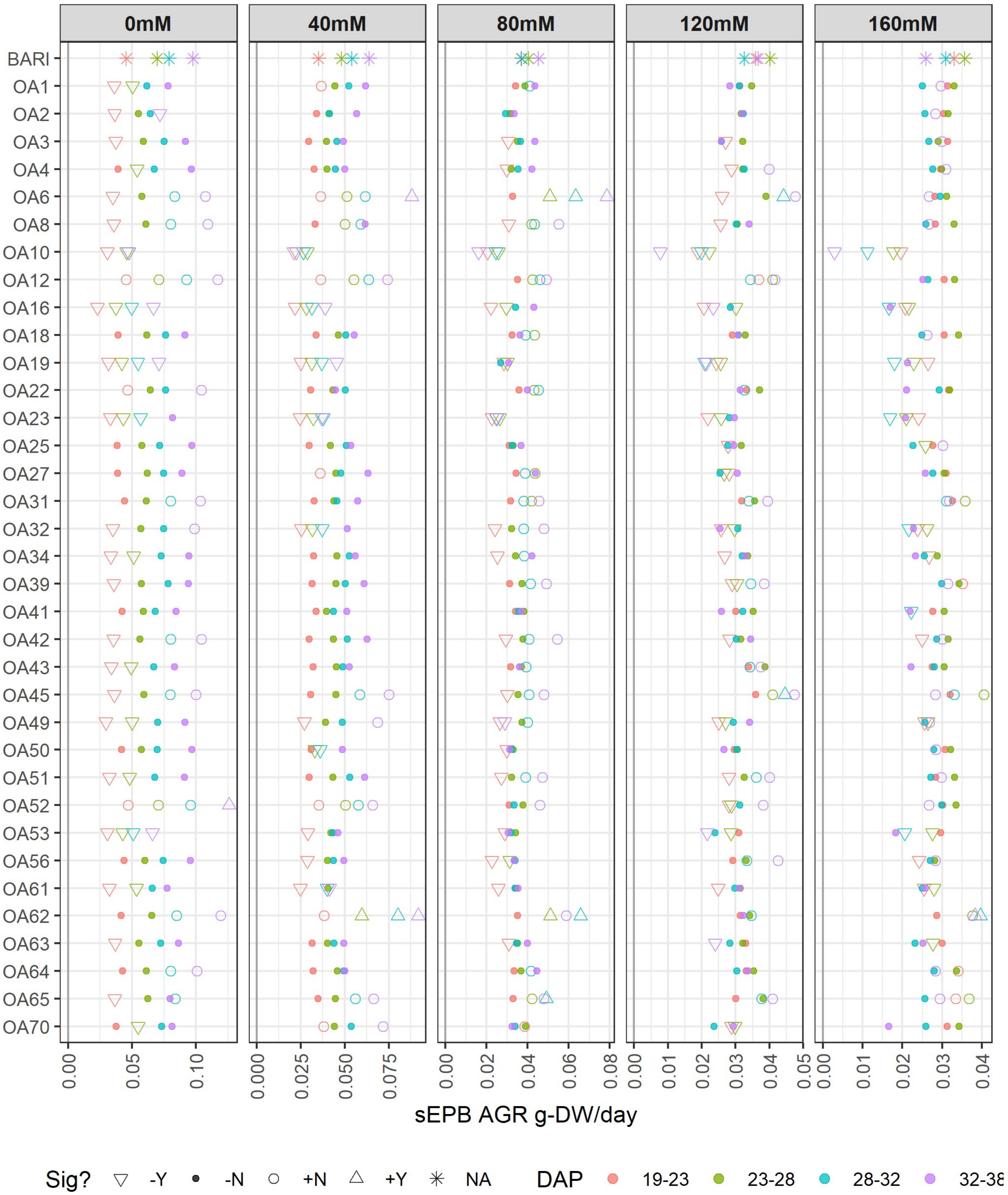
0mM
40mM
80mM
120mM
160mM
BARI
OA1
OA2
OA3
OA4
OA6
OA8
OA10
OA12
OA16
OA18
OA19
OA22
OA23
OA25
OA27
OA31
OA32
OA34
OA39
OA41
OA42
OA43
OA45
OA49
OA50
OA51
OA52
OA53
OA56
OA61
OA62
OA63
OA64
OA65
OA70
0.00 0.05 0.10
0.000 0.025 0.050 0.075
0.00 0.02 0.04 0.06 0.08
0.00 0.01 0.02 0.03 0.04 0.05
0.00 0.01 0.02 0.03 0.04
sEPB AGR g-DW/day
Sig? -Y -N +N +Y NA
DAP 19-23 23-28 28-32 32-38

Figure S13. NaCl 0mM

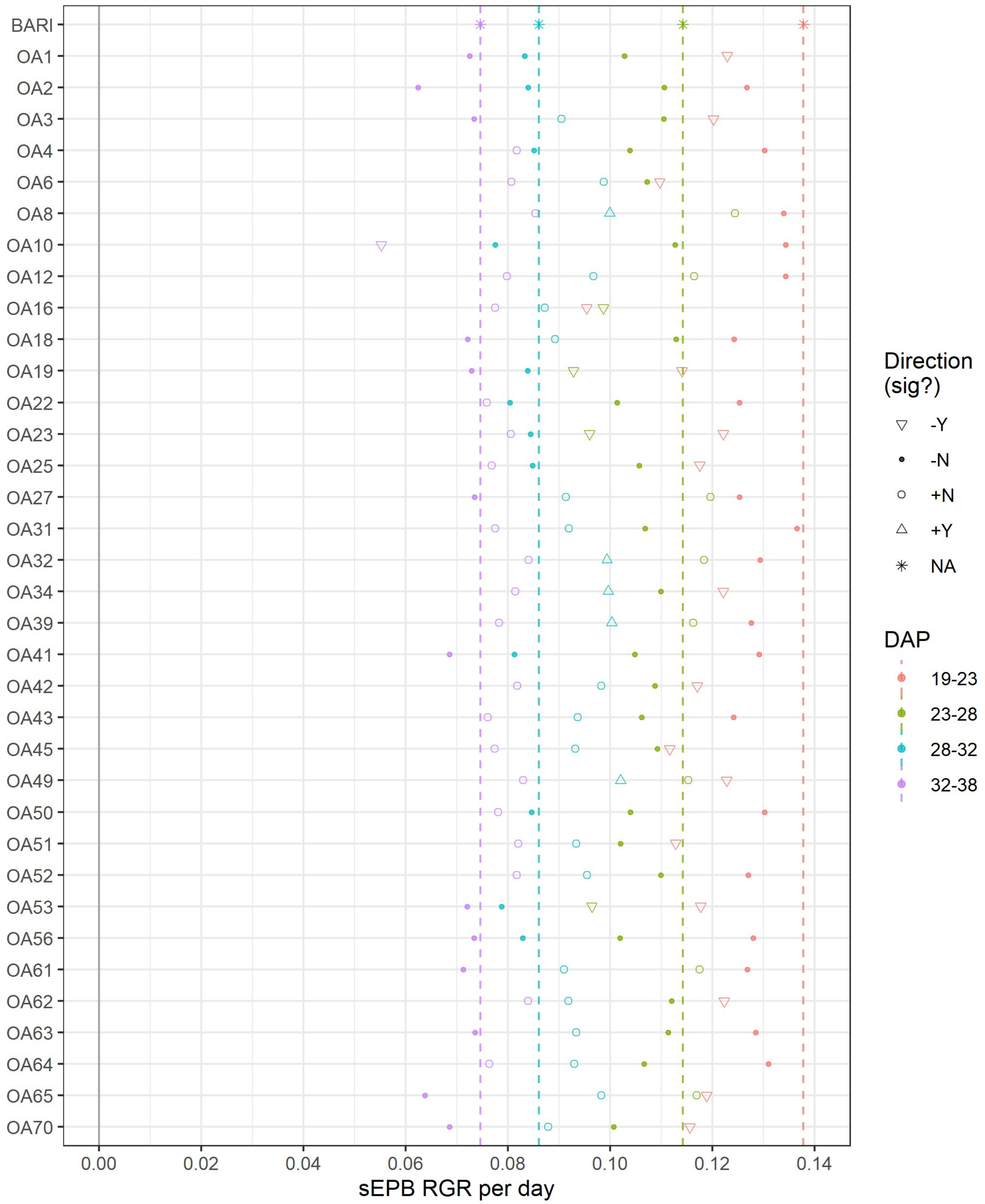

Figure S14. NaCl 40mM

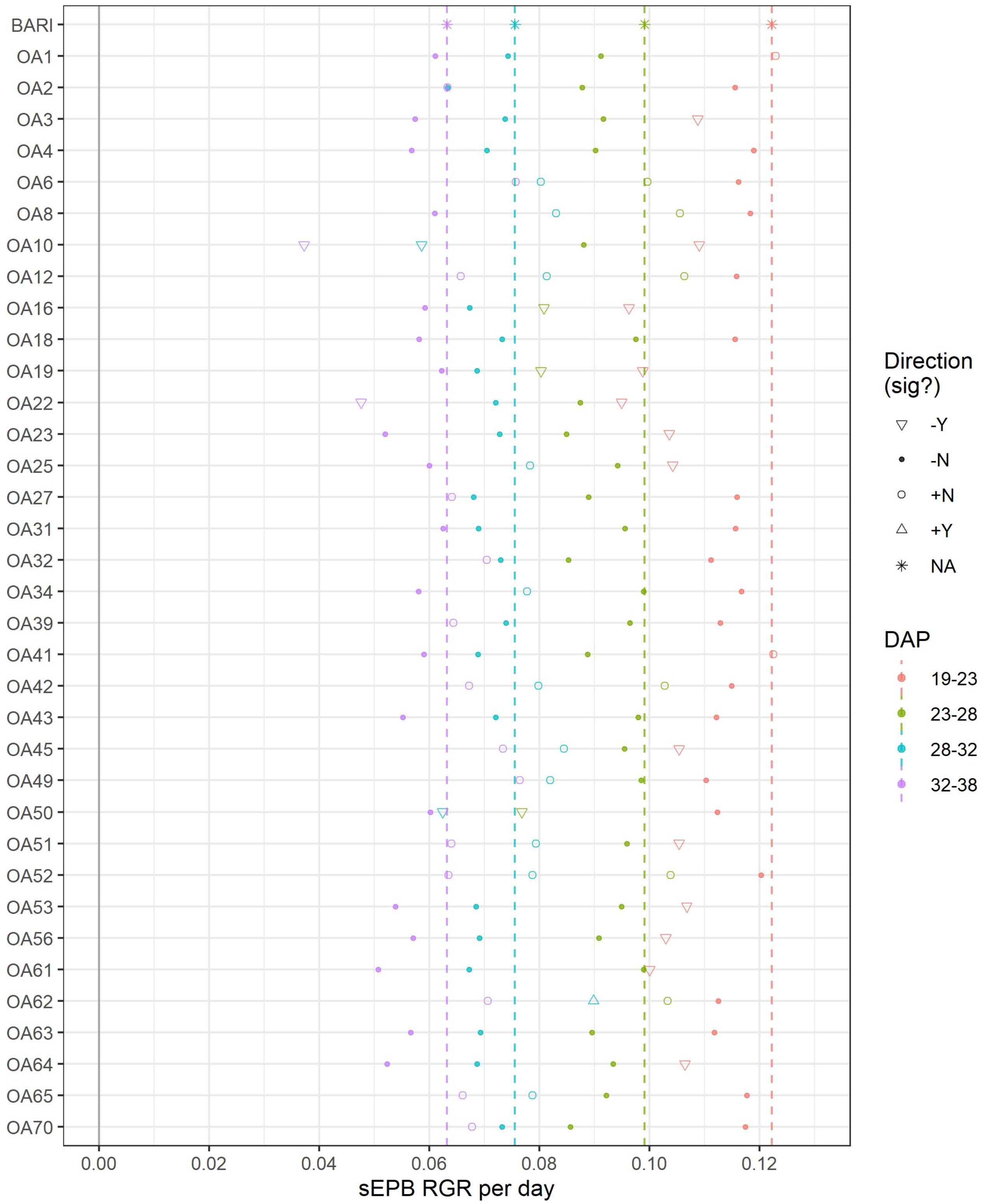

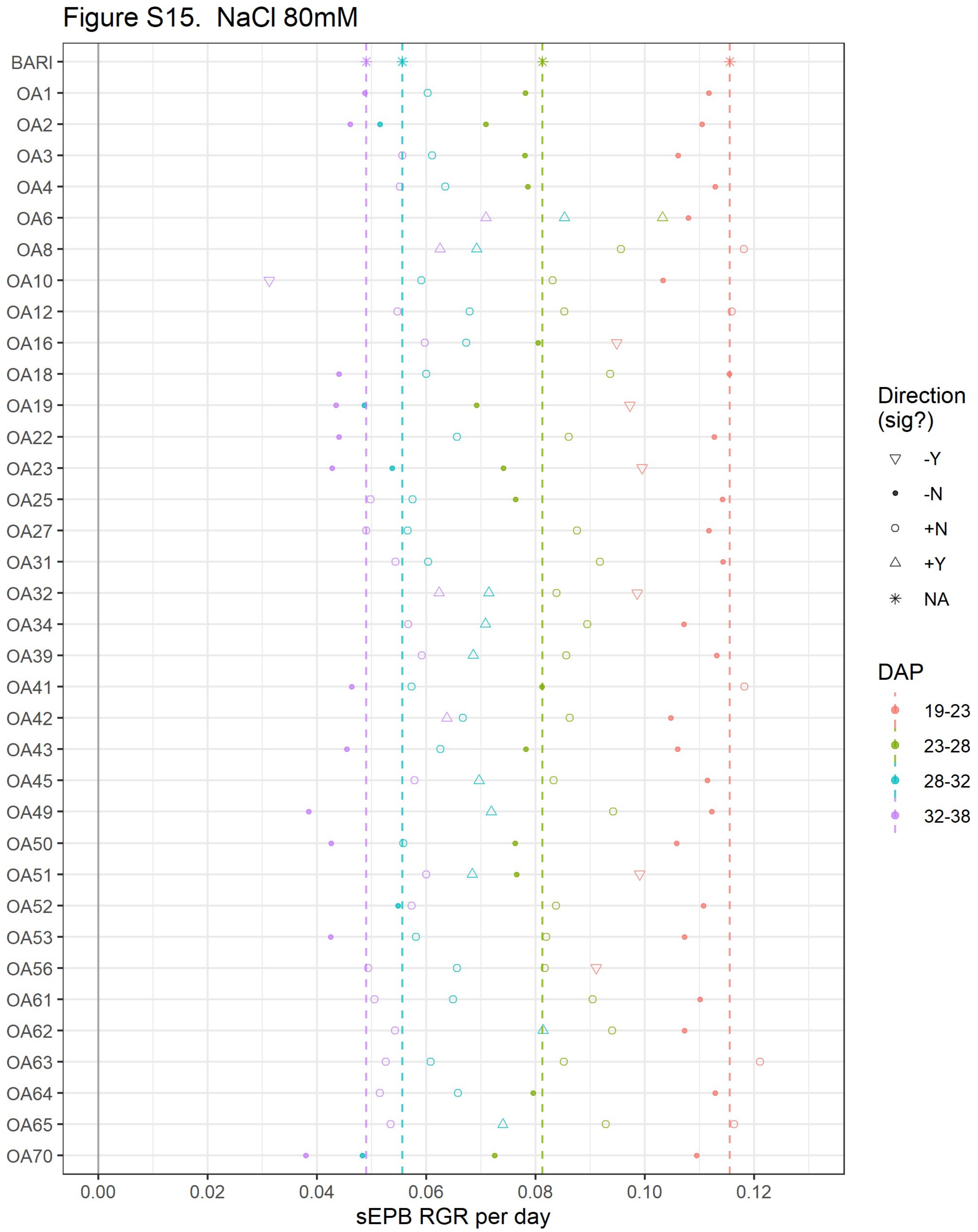

Figure S15. NaCl 80mM
BARI
OA1
OA2
OA3
OA4
OA6
OA8
OA10
OA12
OA16
OA18
OA19
OA22
OA23
OA25
OA27
OA31
OA32
OA34
OA39
OA41
OA42
OA43
OA45
OA49
OA50
OA51
OA52
OA53
OA56
OA61
OA62
OA63
OA64
OA65
OA70
0.00
0.02
0.04
0.06
0.08
0.10
0.12
sEPB RGR per day
Direction (sig?)
-Y
-N
+N
+Y
NA
DAP
19-23
23-28
28-32
32-38

Figure S16.  NaCl 120mM

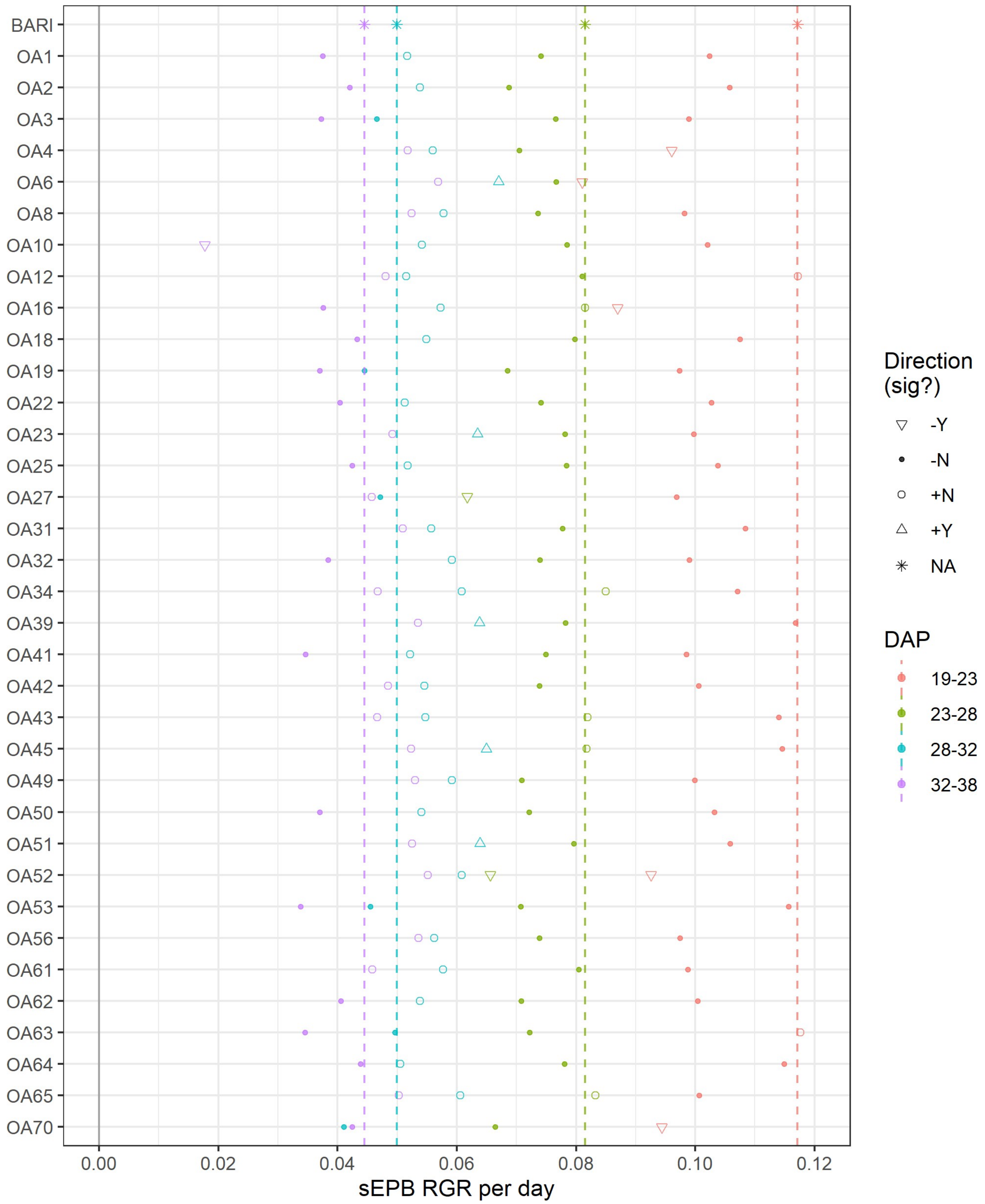

Figure S17. NaCl 160mM

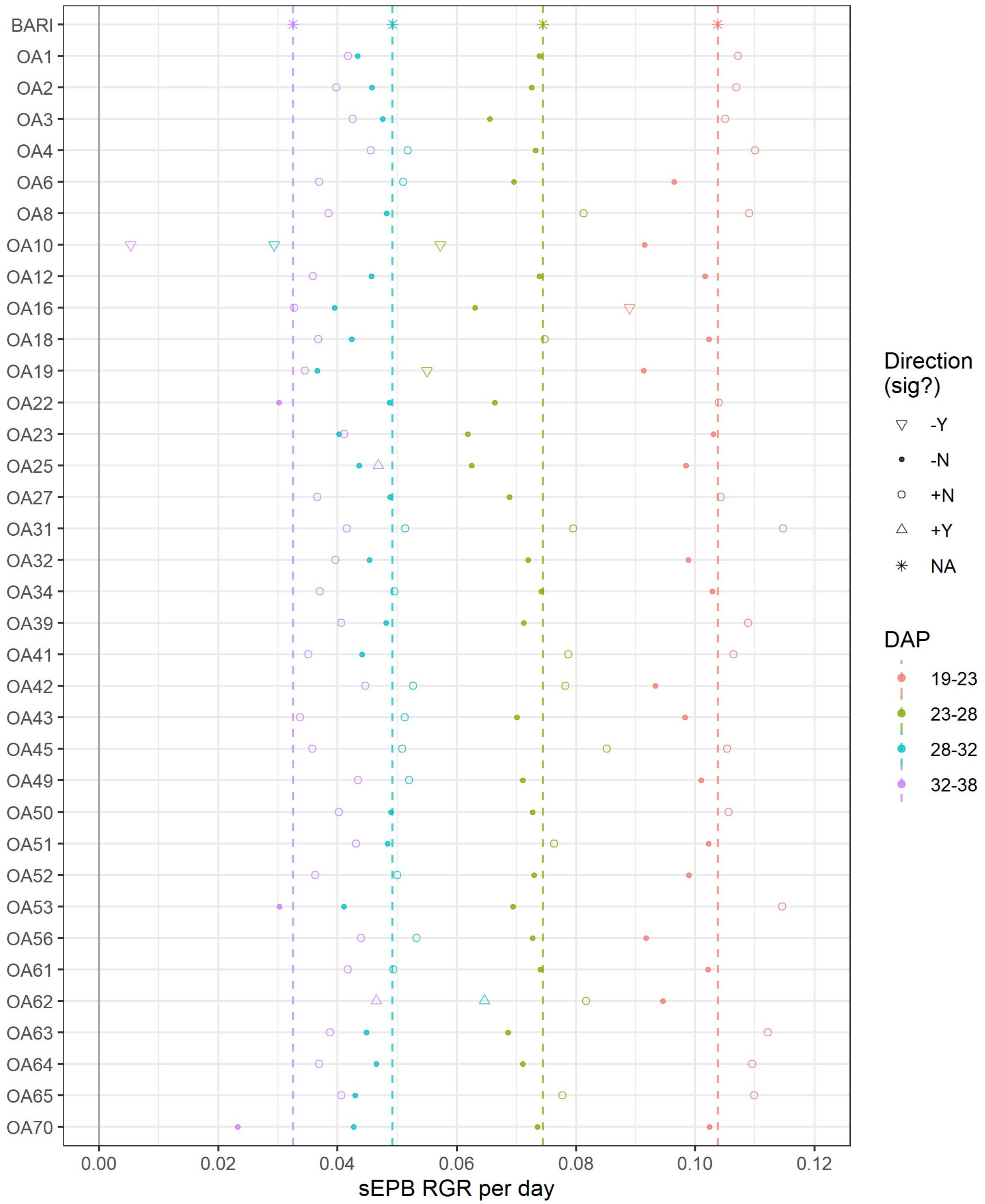

Figure S18.

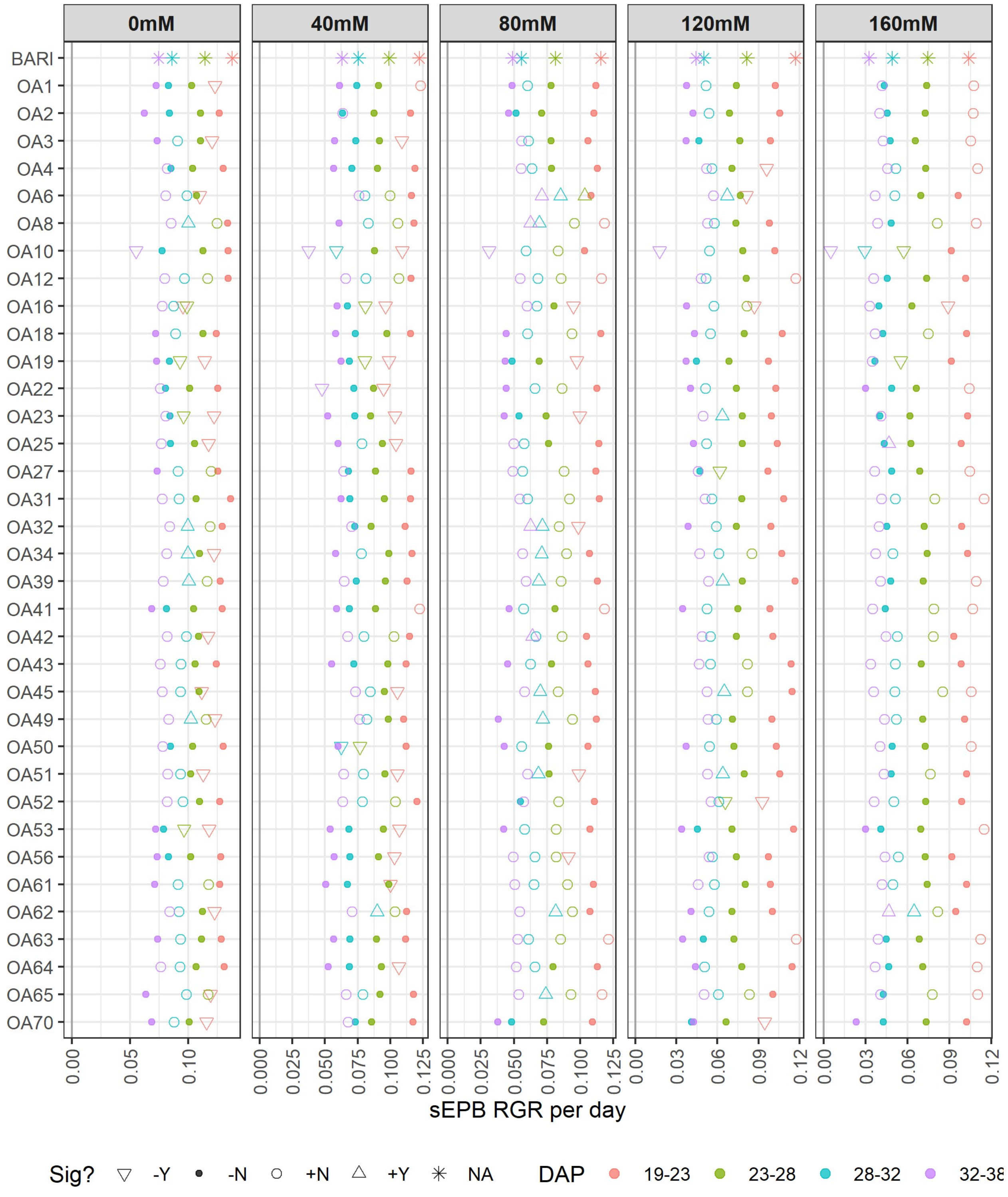
0mM
40mM
80mM
120mM
160mM
BARI
OA1
OA2
OA3
OA4
OA6
OA8
OA10
OA12
OA16
OA18
OA19
OA22
OA23
OA25
OA27
OA31
OA32
OA34
OA39
OA41
OA42
OA43
OA45
OA49
OA50
OA51
OA52
OA53
OA56
OA61
OA62
OA63
OA64
OA65
OA70
0.00 0.05 0.10
0.000 0.025 0.050 0.075 0.100 0.125
0.000 0.025 0.050 0.075 0.100 0.125
0.00 0.03 0.06 0.09 0.12
0.00 0.03 0.06 0.09 0.12
sEPB RGR per day
Sig?
-Y
-N
+N
+Y
NA
DAP
19-23
23-28
28-32
32-38

Figure S19.

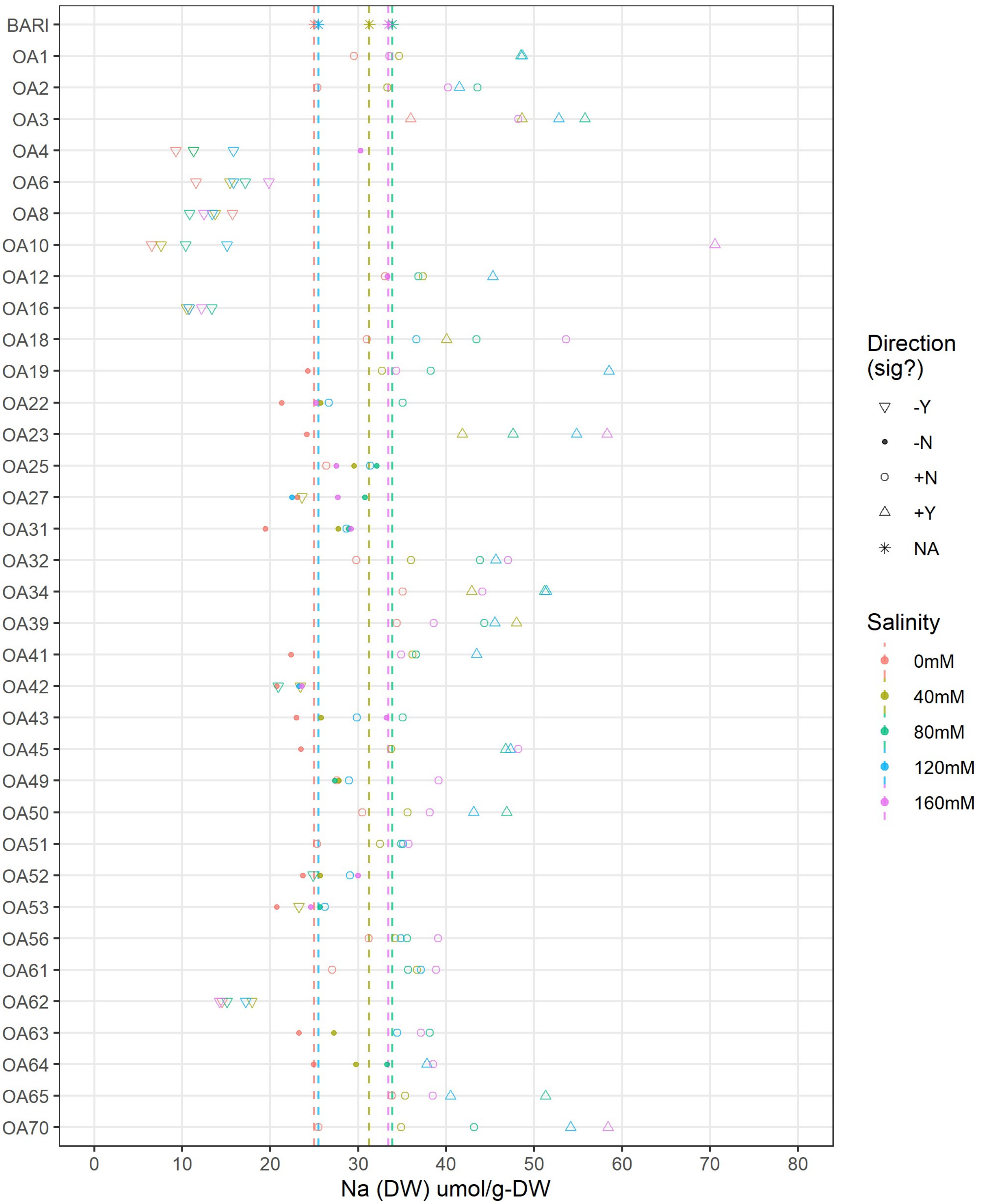
BARI
OA1
OA2
OA3
OA4
OA6
OA8
OA10
OA12
OA16
OA18
OA19
OA22
OA23
OA25
OA27
OA31
OA32
OA34
OA39
OA41
OA42
OA43
OA45
OA49
OA50
OA51
OA52
OA53
OA56
OA61
OA62
OA63
OA64
OA65
OA70
0
10
20
30
40
50
60
70
80
Na (DW) umol/g-DW
Direction (sig?)
-Y
-N
+N
+Y
NA
Salinity
0mM
40mM
80mM
120mM
160mM

Figure S20.

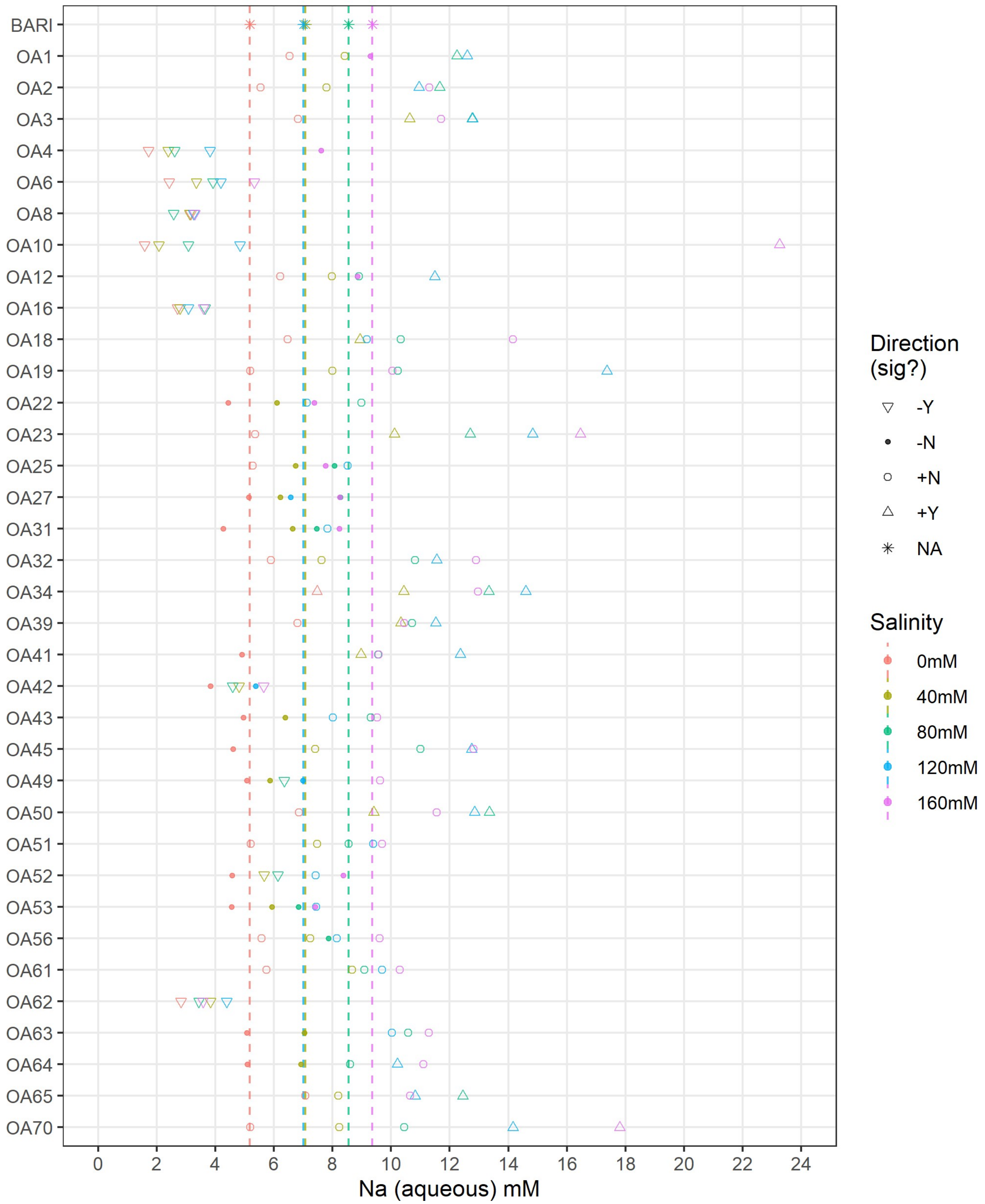

BARI
OA1
OA2
OA3
OA4
OA6
OA8
OA10
OA12
OA16
OA18
OA19
OA22
OA23
OA25
OA27
OA31
OA32
OA34
OA39
OA41
OA42
OA43
OA45
OA49
OA50
OA51
OA52
OA53
OA56
OA61
OA62
OA63
OA64
OA65
OA70
0 2 4 6 8 10 12 14 16 18 20 22 24
Na (aqueous) mM
Direction (sig?)
-Y
-N
+N
+Y
NA
Salinity
0mM
40mM
80mM
120mM
160mM

Figure S21.

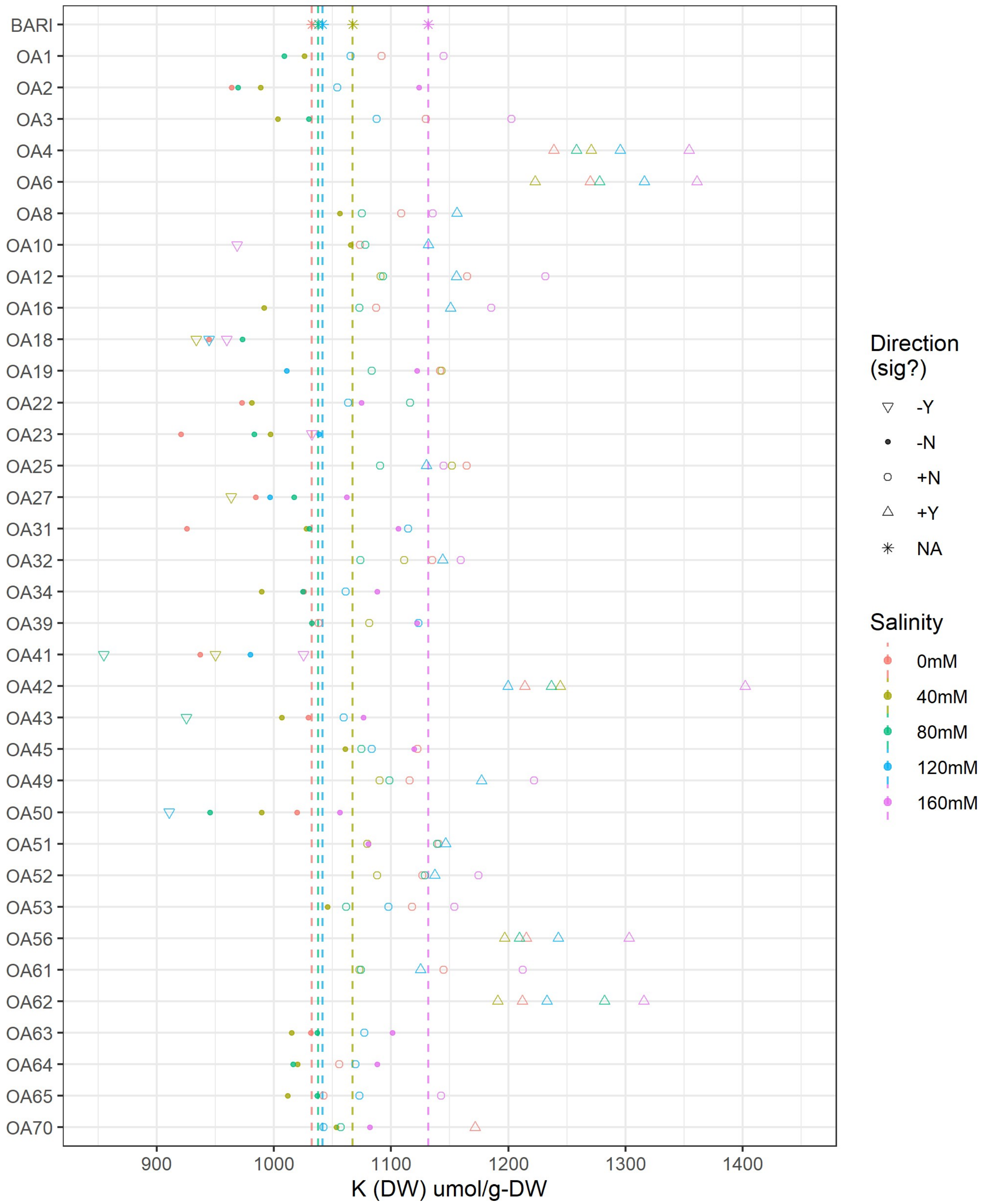
BARI
OA1
OA2
OA3
OA4
OA6
OA8
OA10
OA12
OA16
OA18
OA19
OA22
OA23
OA25
OA27
OA31
OA32
OA34
OA39
OA41
OA42
OA43
OA45
OA49
OA50
OA51
OA52
OA53
OA56
OA61
OA62
OA63
OA64
OA65
OA70
900
1000
1100
1200
1300
1400
K (DW) umol/g-DW
Direction (sig?)
-Y
-N
+N
+Y
NA
Salinity
0mM
40mM
80mM
120mM
160mM

Figure S22.

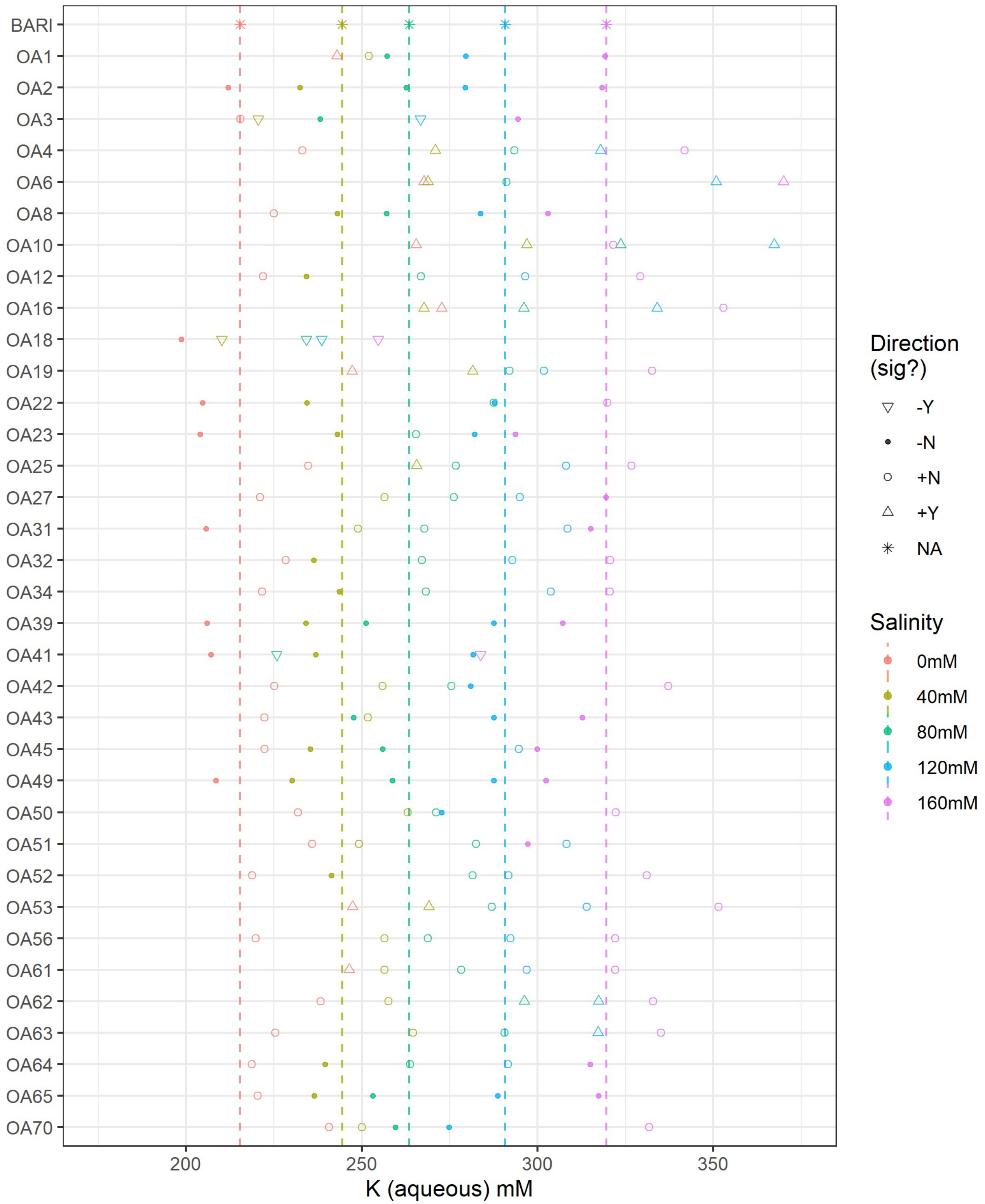

BARI
OA1
OA2
OA3
OA4
OA6
OA8
OA10
OA12
OA16
OA18
OA19
OA22
OA23
OA25
OA27
OA31
OA32
OA34
OA39
OA41
OA42
OA43
OA45
OA49
OA50
OA51
OA52
OA53
OA56
OA61
OA62
OA63
OA64
OA65
OA70
200
250
300
350
K (aqueous) mM
Direction (sig?)
-Y
-N
+N
+Y
NA
Salinity
0mM
40mM
80mM
120mM
160mM

Figure S23.

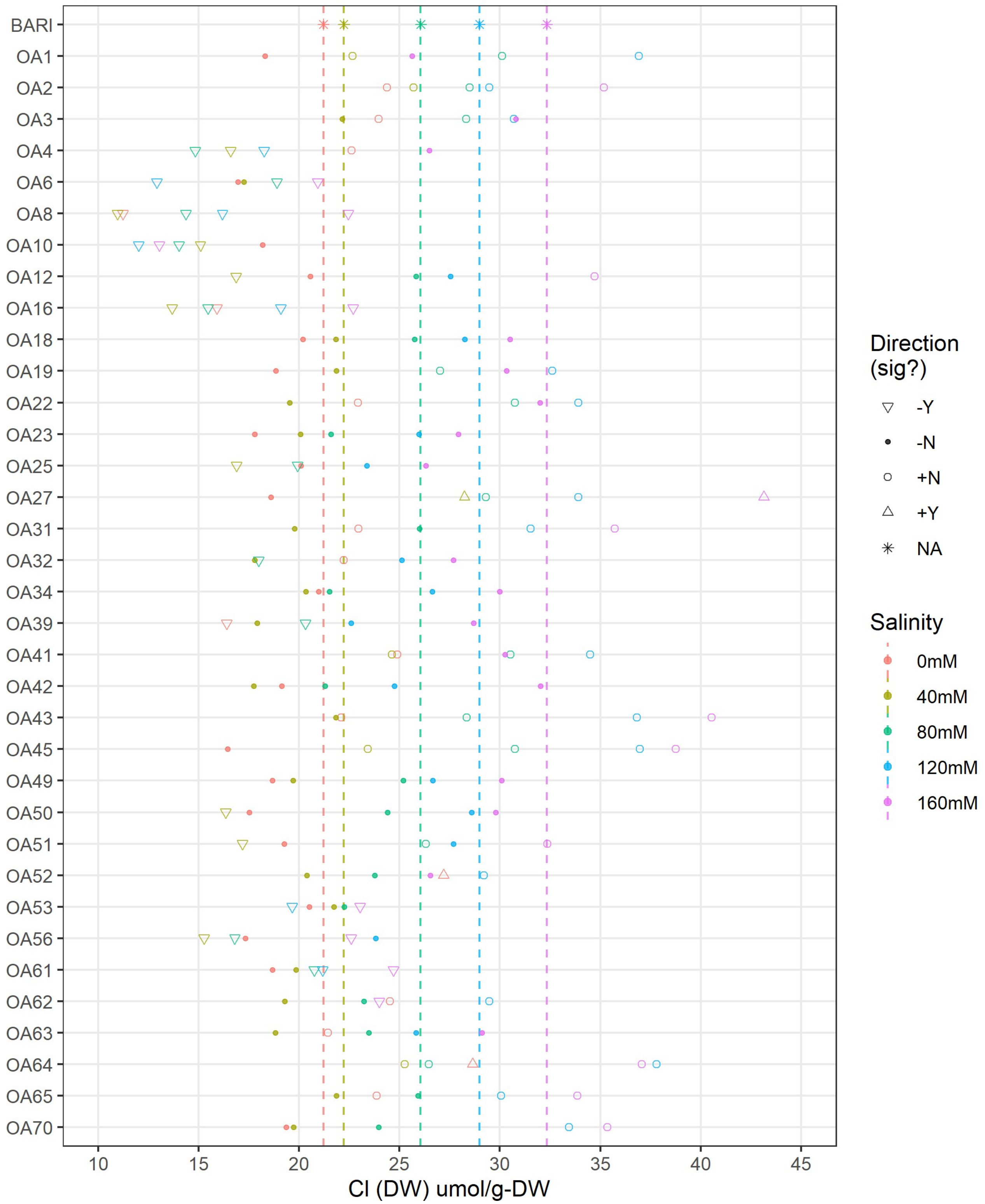

BARI
OA1
OA2
OA3
OA4
OA6
OA8
OA10
OA12
OA16
OA18
OA19
OA22
OA23
OA25
OA27
OA31
OA32
OA34
OA39
OA41
OA42
OA43
OA45
OA49
OA50
OA51
OA52
OA53
OA56
OA61
OA62
OA63
OA64
OA65
OA70
10
15
20
25
30
35
40
45
Cl (DW) umol/g-DW
Direction (sig?)
-Y
-N
+N
+Y
NA
Salinity
0mM
40mM
80mM
120mM
160mM

Figure S24.

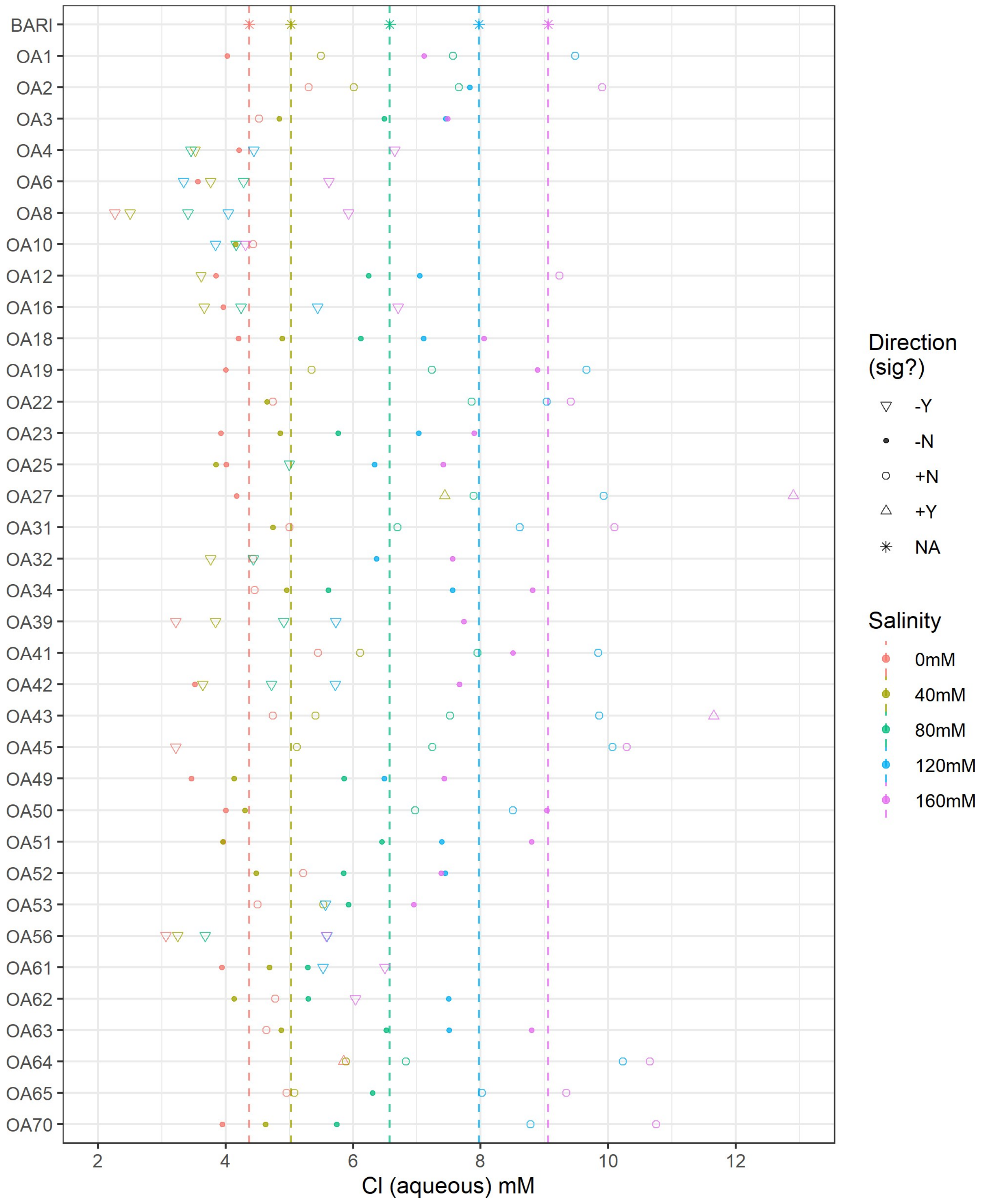
BARI
OA1
OA2
OA3
OA4
OA6
OA8
OA10
OA12
OA16
OA18
OA19
OA22
OA23
OA25
OA27
OA31
OA32
OA34
OA39
OA41
OA42
OA43
OA45
OA49
OA50
OA51
OA52
OA53
OA56
OA61
OA62
OA63
OA64
OA65
OA70
2
4
6
8
10
12
Cl (aqueous) mM
Direction (sig?)
-Y
-N
+N
+Y
NA
Salinity
0mM
40mM
80mM
120mM
160mM

Figure S25.

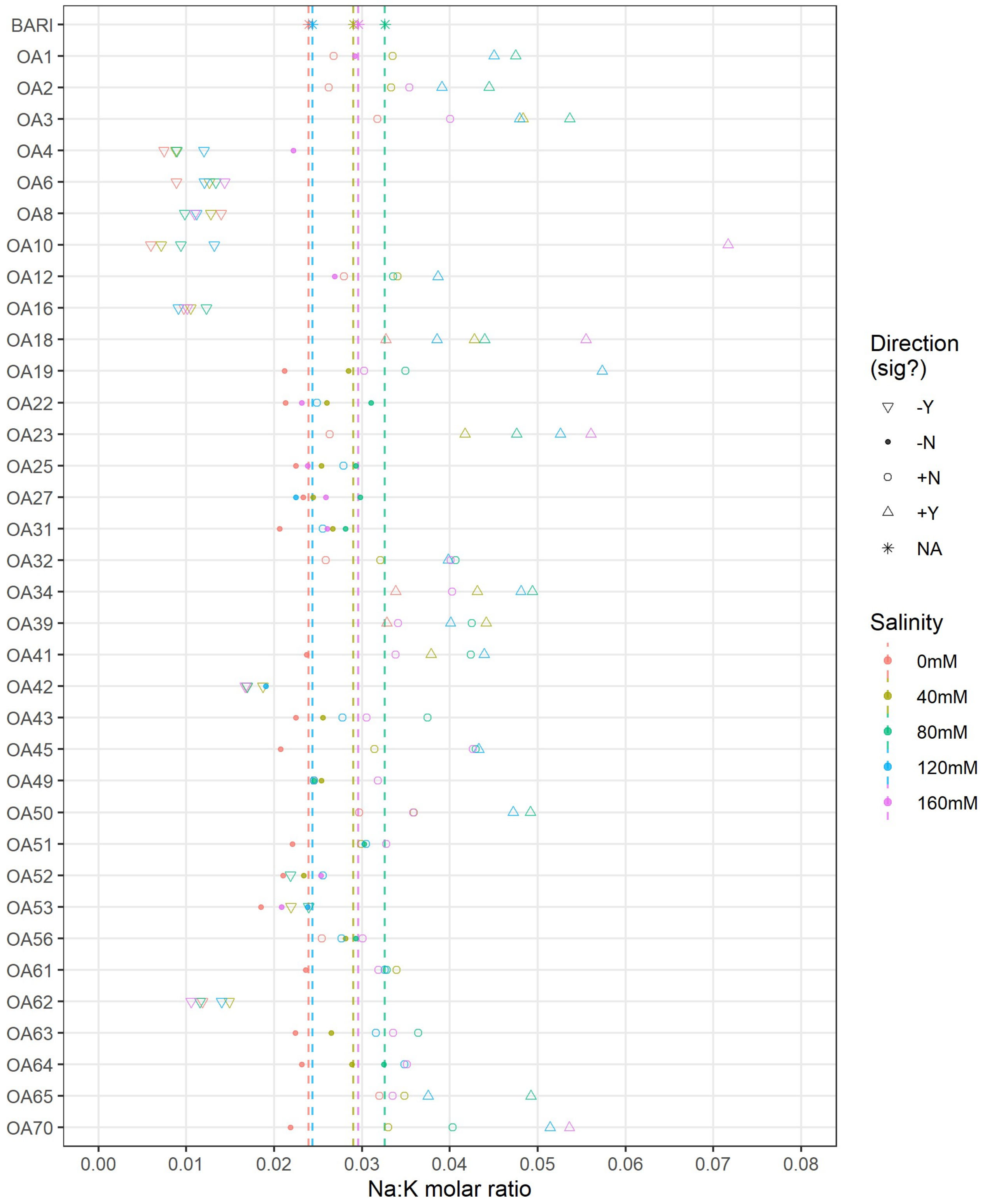

BARI
OA1
OA2
OA3
OA4
OA6
OA8
OA10
OA12
OA16
OA18
OA19
OA22
OA23
OA25
OA27
OA31
OA32
OA34
OA39
OA41
OA42
OA43
OA45
OA49
OA50
OA51
OA52
OA53
OA56
OA61
OA62
OA63
OA64
OA65
OA70
0.00
0.01
0.02
0.03
0.04
0.05
0.06
0.07
0.08
Na:K molar ratio
Direction (sig?)
-Y
-N
+N
+Y
NA
Salinity
0mM
40mM
80mM
120mM
160mM

Figure S26.

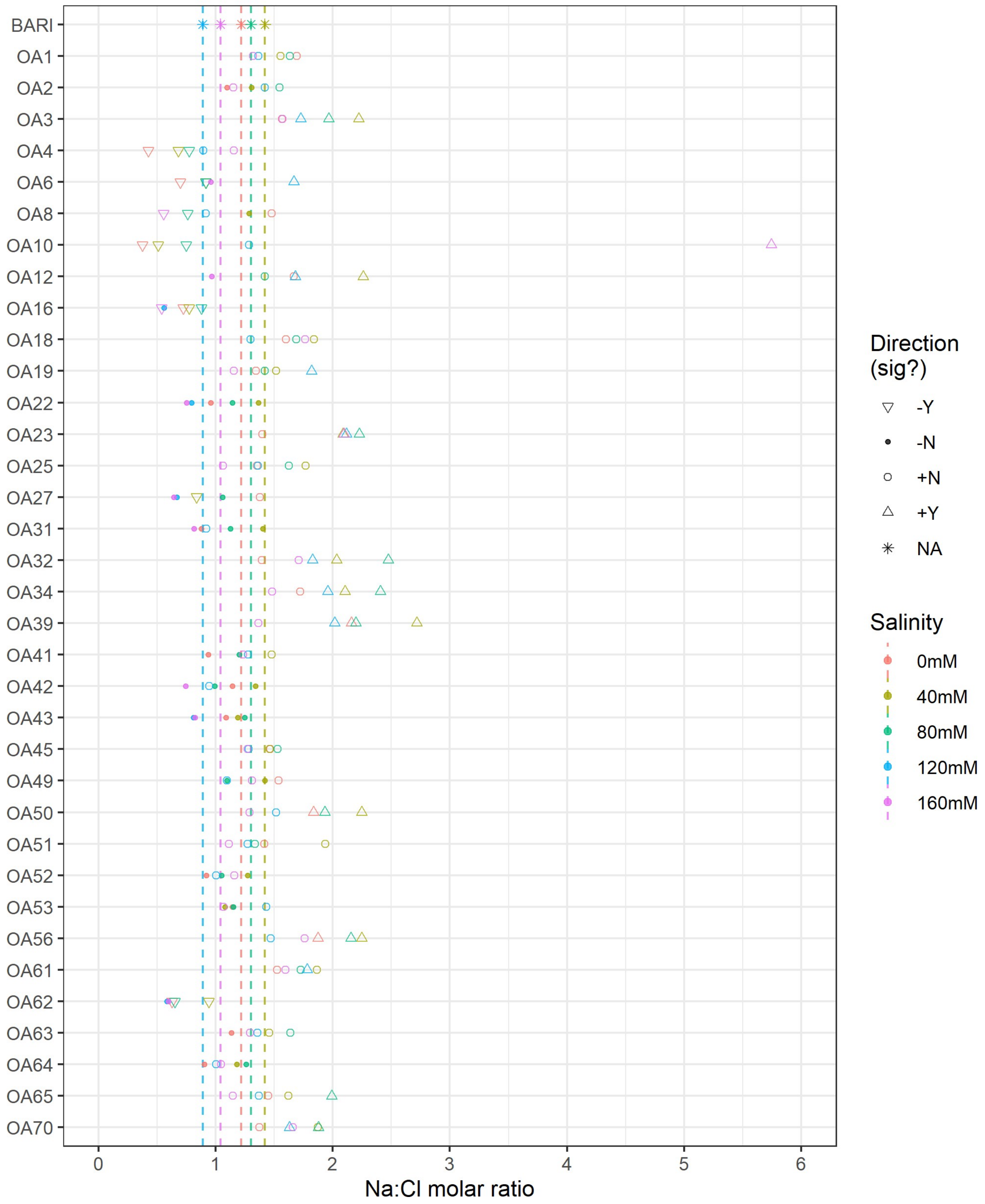
BARI
OA1
OA2
OA3
OA4
OA6
OA8
OA10
OA12
OA16
OA18
OA19
OA22
OA23
OA25
OA27
OA31
OA32
OA34
OA39
OA41
OA42
OA43
OA45
OA49
OA50
OA51
OA52
OA53
OA56
OA61
OA62
OA63
OA64
OA65
OA70
0
1
2
3
4
5
6
Na:Cl molar ratio
Direction (sig?)
-Y
-N
+N
+Y
NA
Salinity
0mM
40mM
80mM
120mM
160mM

Figure S27.

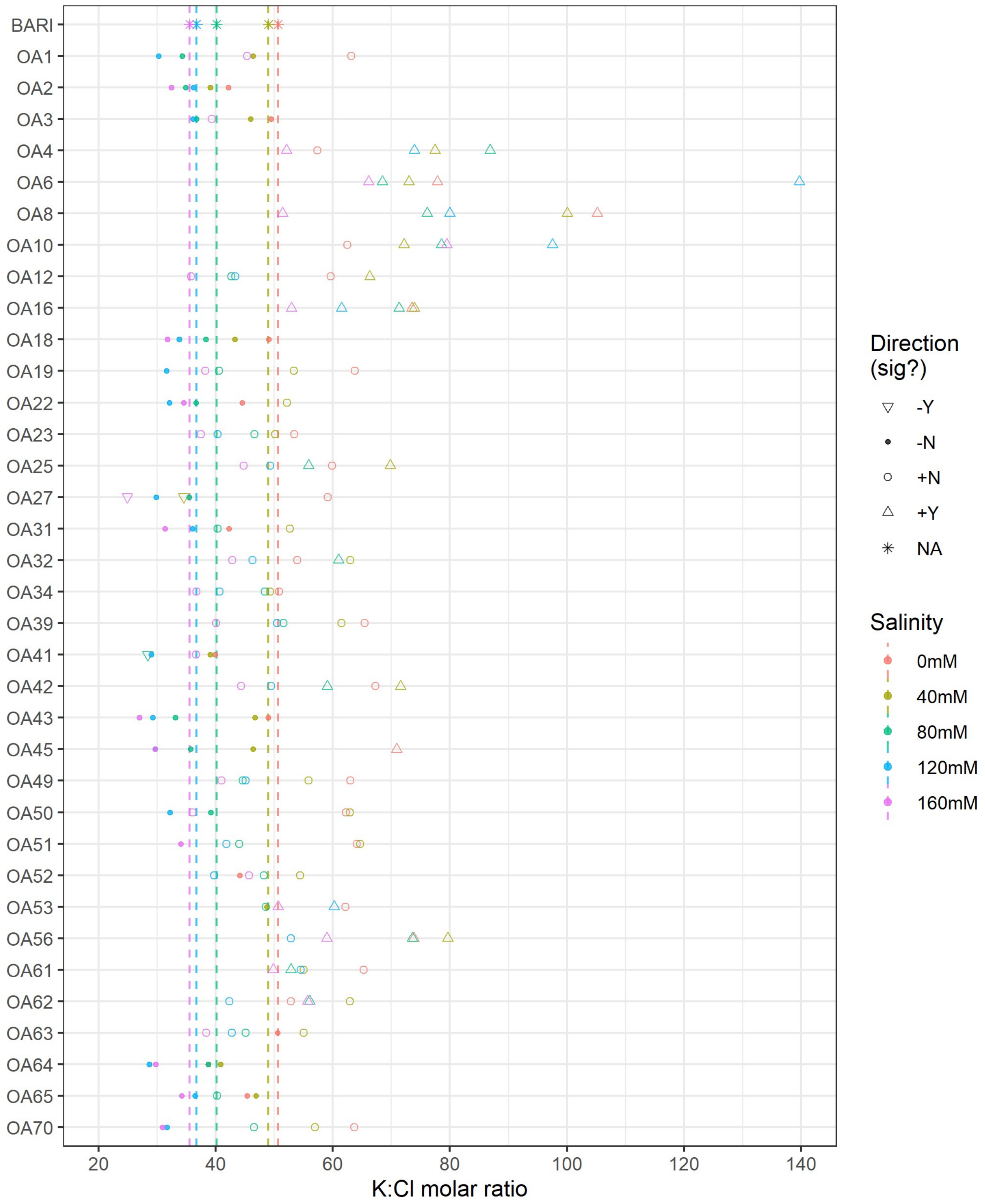
BARI
OA1
OA2
OA3
OA4
OA6
OA8
OA10
OA12
OA16
OA18
OA19
OA22
OA23
OA25
OA27
OA31
OA32
OA34
OA39
OA41
OA42
OA43
OA45
OA49
OA50
OA51
OA52
OA53
OA56
OA61
OA62
OA63
OA64
OA65
OA70
20
40
60
80
100
120
140
K:Cl molar ratio
Direction (sig?)
-Y
-N
+N
+Y
NA
Salinity
0mM
40mM
80mM
120mM
160mM

Figure S28.

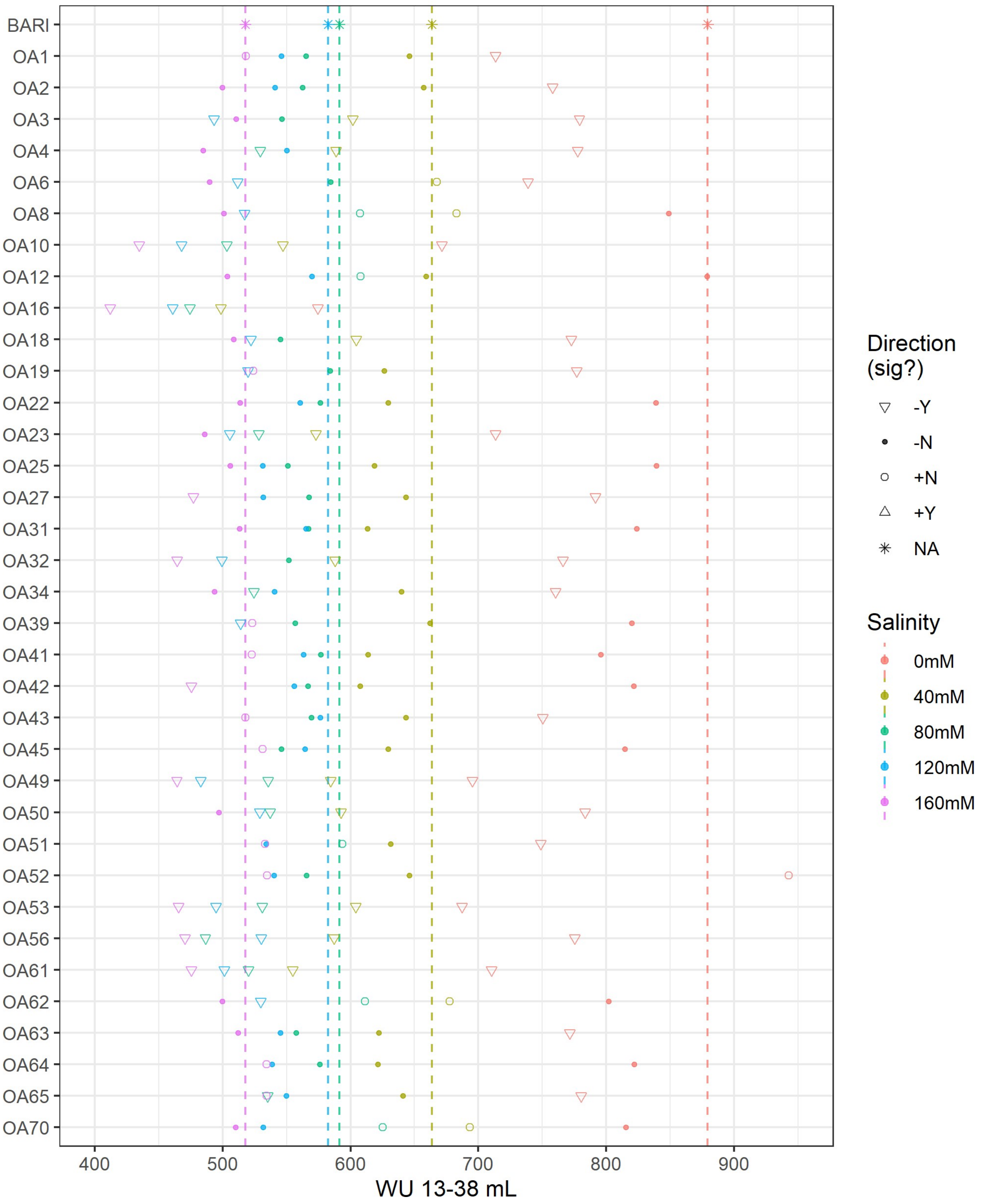

BARI
OA1
OA2
OA3
OA4
OA6
OA8
OA10
OA12
OA16
OA18
OA19
OA22
OA23
OA25
OA27
OA31
OA32
OA34
OA39
OA41
OA42
OA43
OA45
OA49
OA50
OA51
OA52
OA53
OA56
OA61
OA62
OA63
OA64
OA65
OA70
400
500
600
700
800
900
WU 13-38 mL
Direction (sig?)
-Y
-N
+N
+Y
NA
Salinity
0mM
40mM
80mM
120mM
160mM

Figure S29.

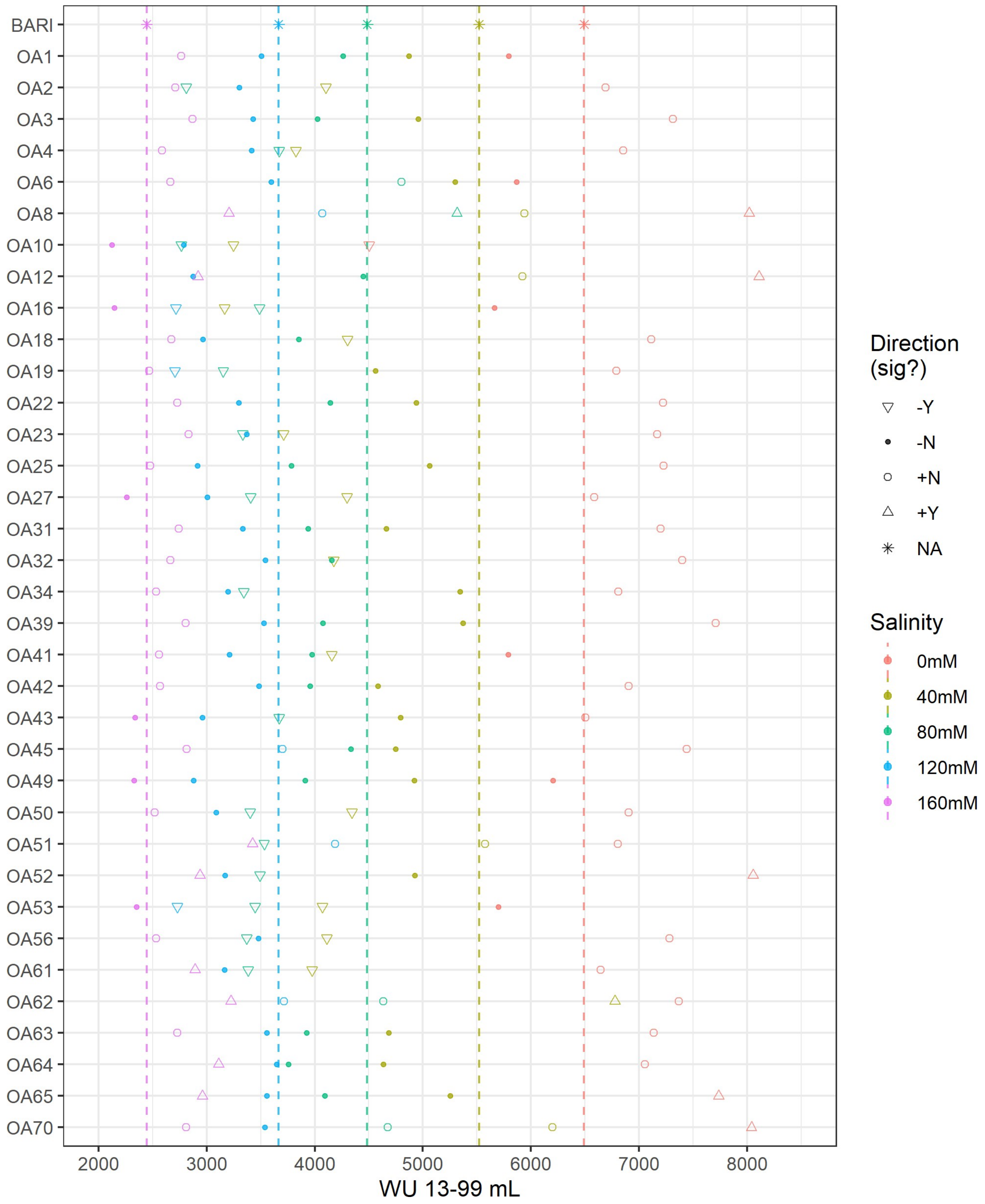

BARI
OA1
OA2
OA3
OA4
OA6
OA8
OA10
OA12
OA16
OA18
OA19
OA22
OA23
OA25
OA27
OA31
OA32
OA34
OA39
OA41
OA42
OA43
OA45
OA49
OA50
OA51
OA52
OA53
OA56
OA61
OA62
OA63
OA64
OA65
OA70
2000
3000
4000
5000
6000
7000
8000
WU 13-99 mL
Direction (sig?)
-Y
-N
+N
+Y
NA
Salinity
0mM
40mM
80mM
120mM
160mM

Figure S30.  NaCl 0mM

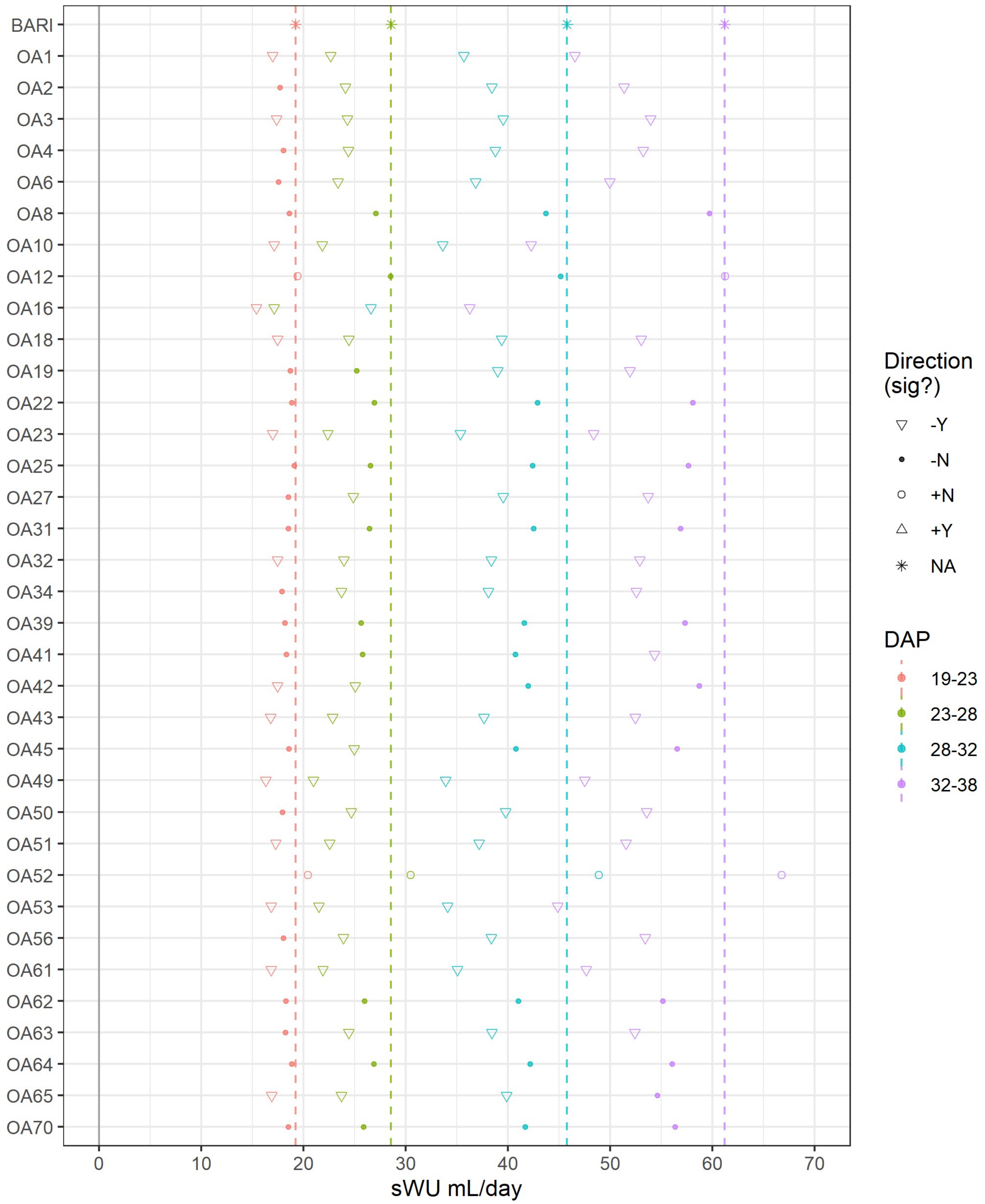

Figure S31. NaCl 40mM

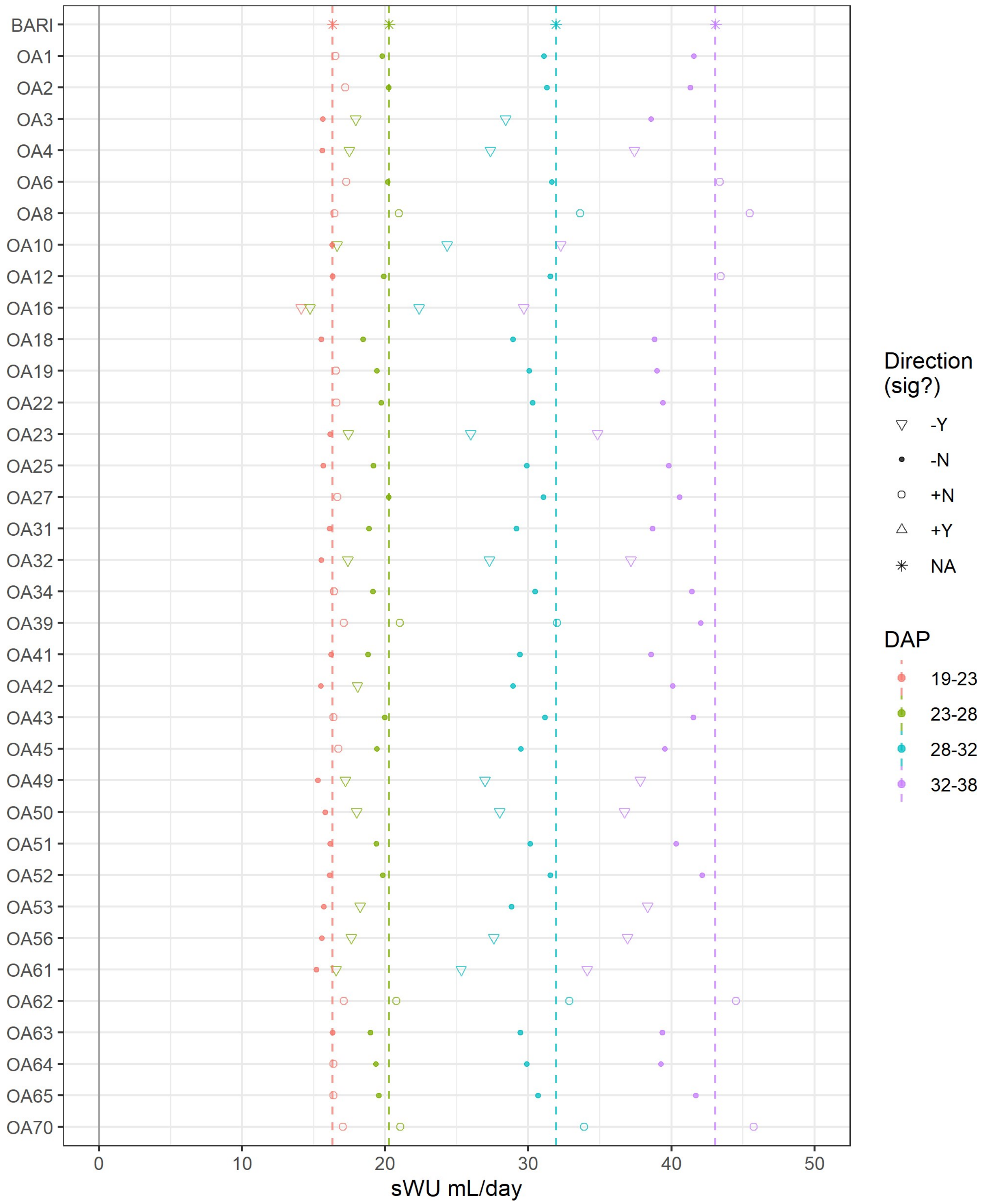

Figure S32.  NaCl 80mM

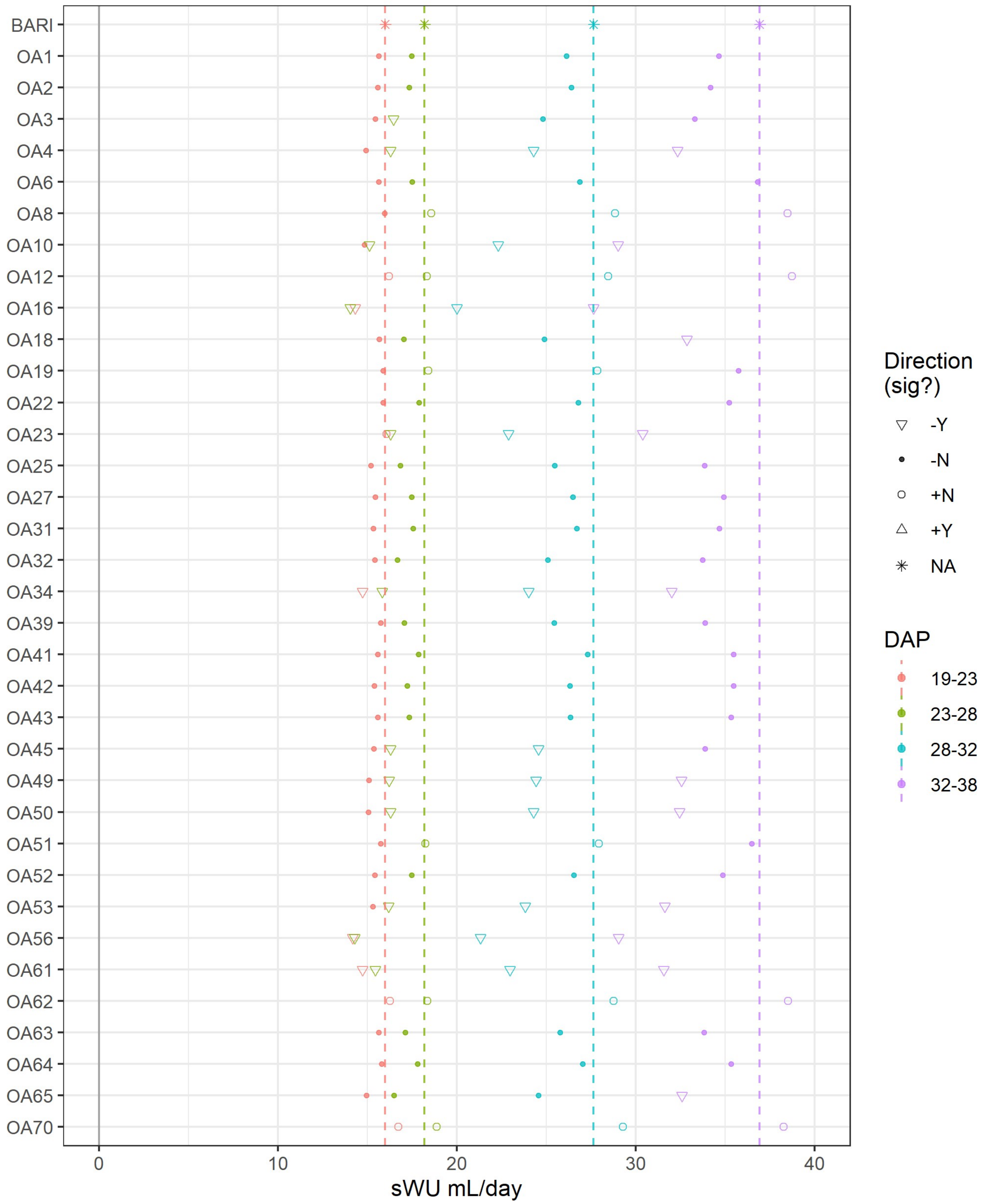

Figure S33. NaCl 120mM

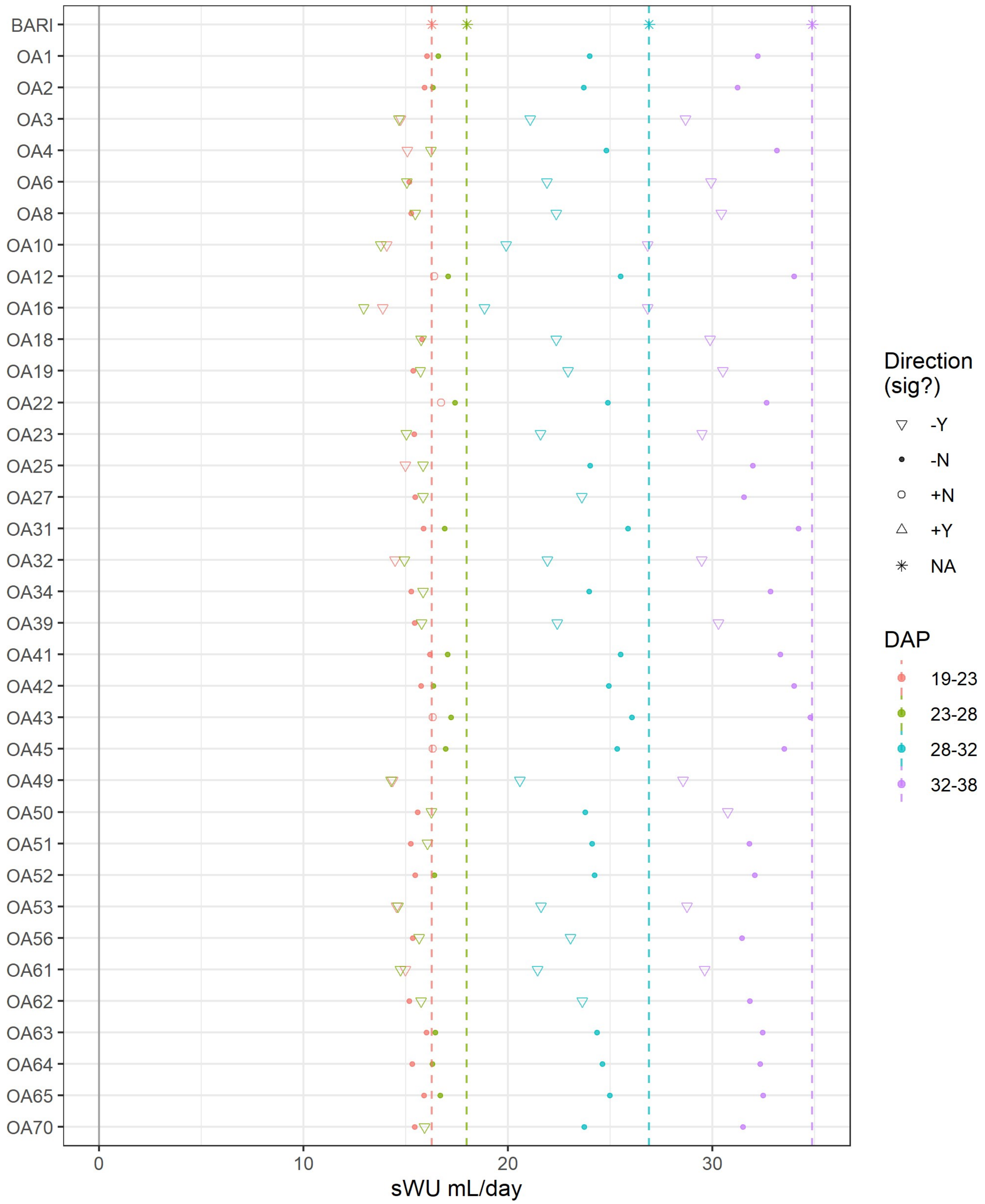

# Figure S34. NaCl 160mM

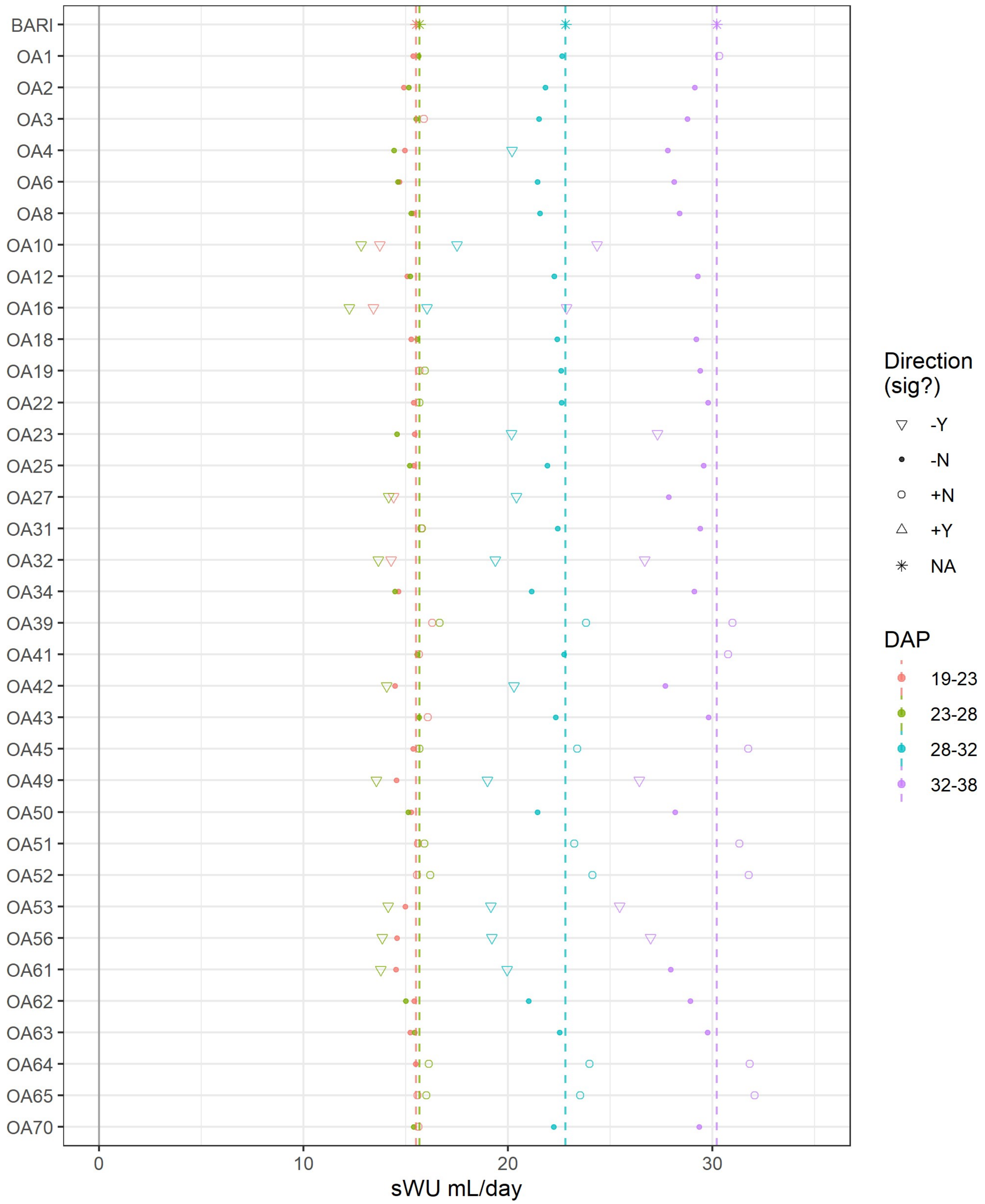

Figure S35.

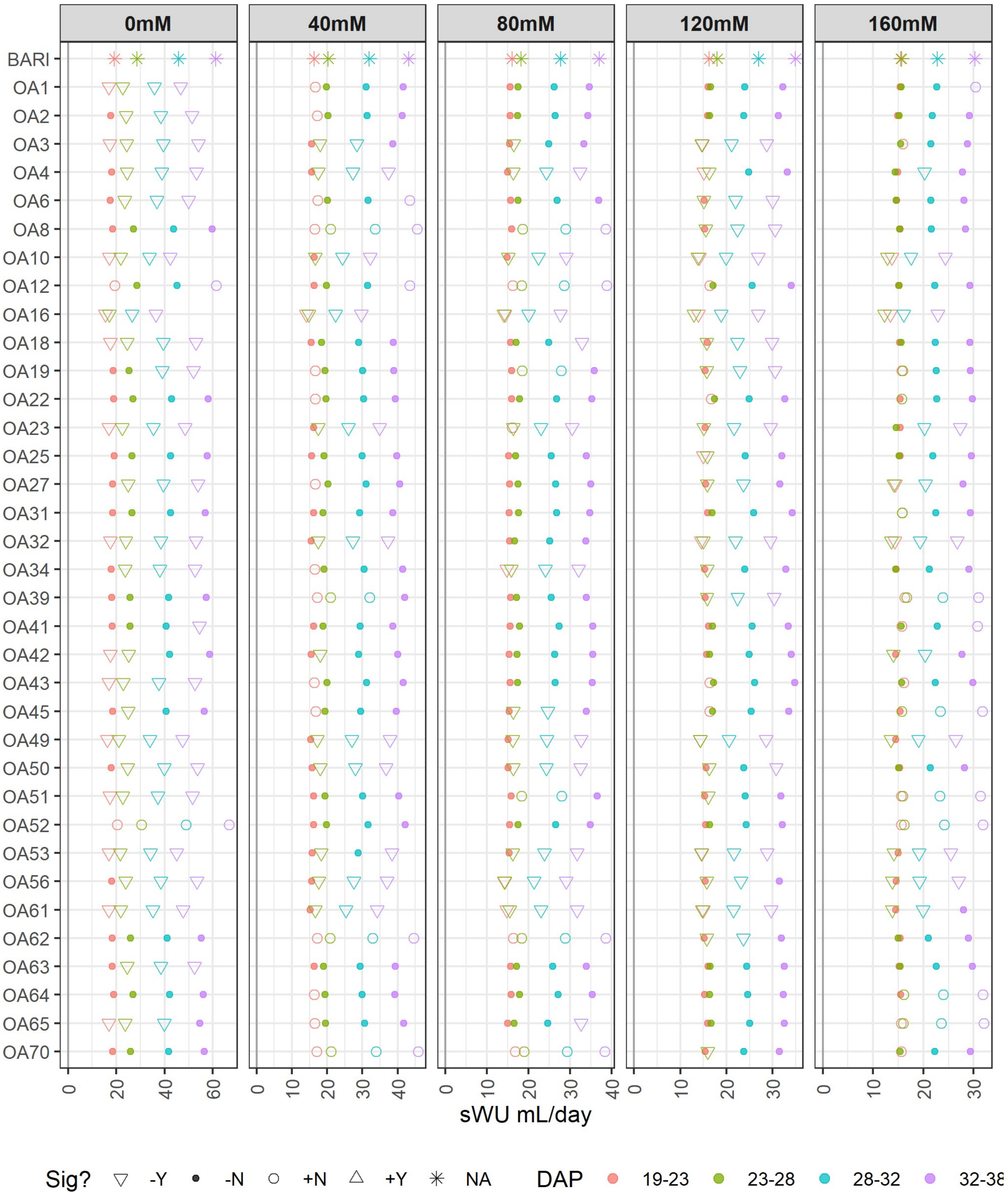
0mM
40mM
80mM
120mM
160mM
BARI
OA1
OA2
OA3
OA4
OA6
OA8
OA10
OA12
OA16
OA18
OA19
OA22
OA23
OA25
OA27
OA31
OA32
OA34
OA39
OA41
OA42
OA43
OA45
OA49
OA50
OA51
OA52
OA53
OA56
OA61
OA62
OA63
OA64
OA65
OA70
0 20 40 60
0 10 20 30 40
0 10 20 30 40
0 10 20 30
0 10 20 30
sWU mL/day
Sig? -Y -N +N +Y NA
DAP 19-23 23-28 28-32 32-38

Figure S36. NaCl 0mM

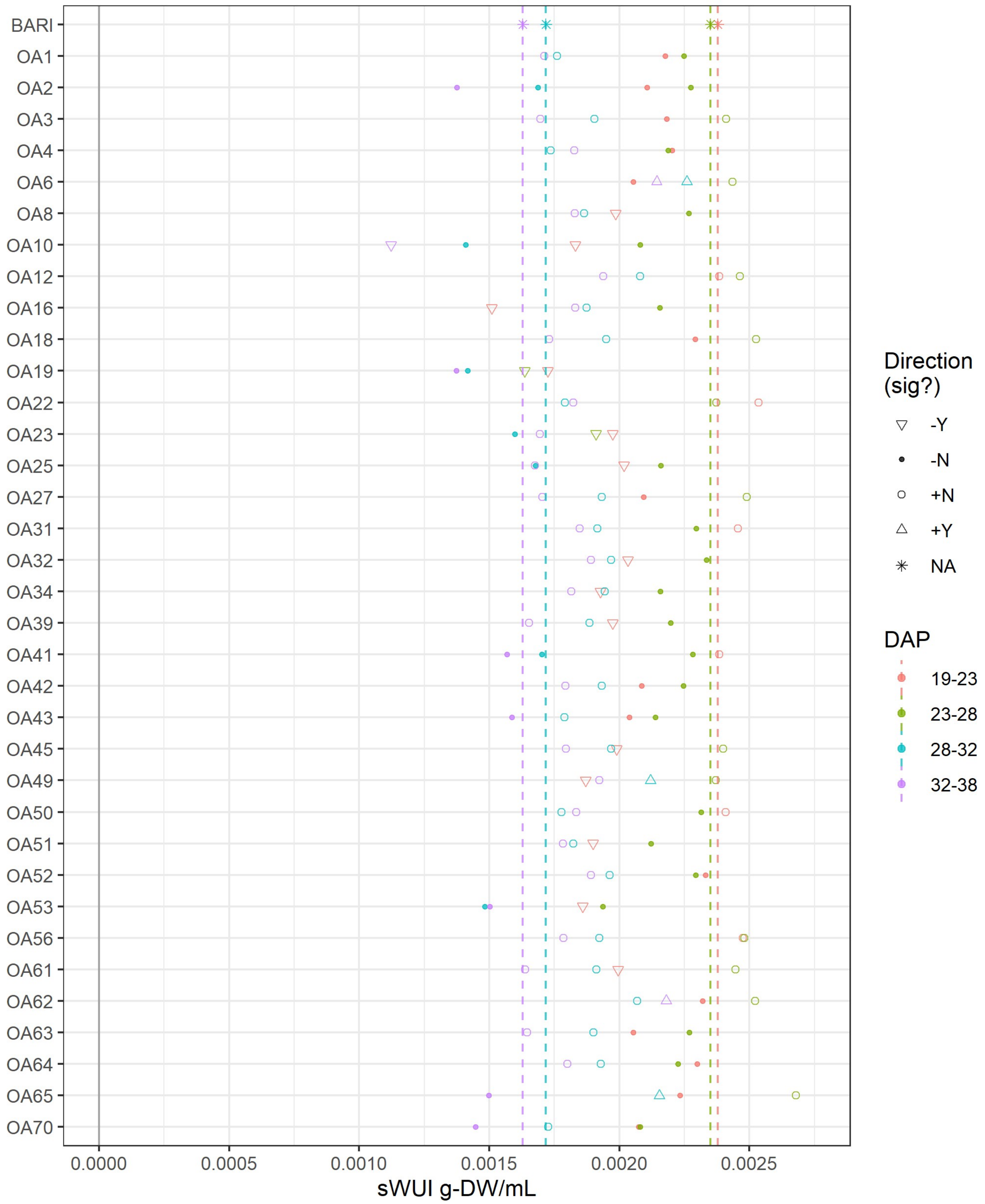

Figure S37.  NaCl 40mM

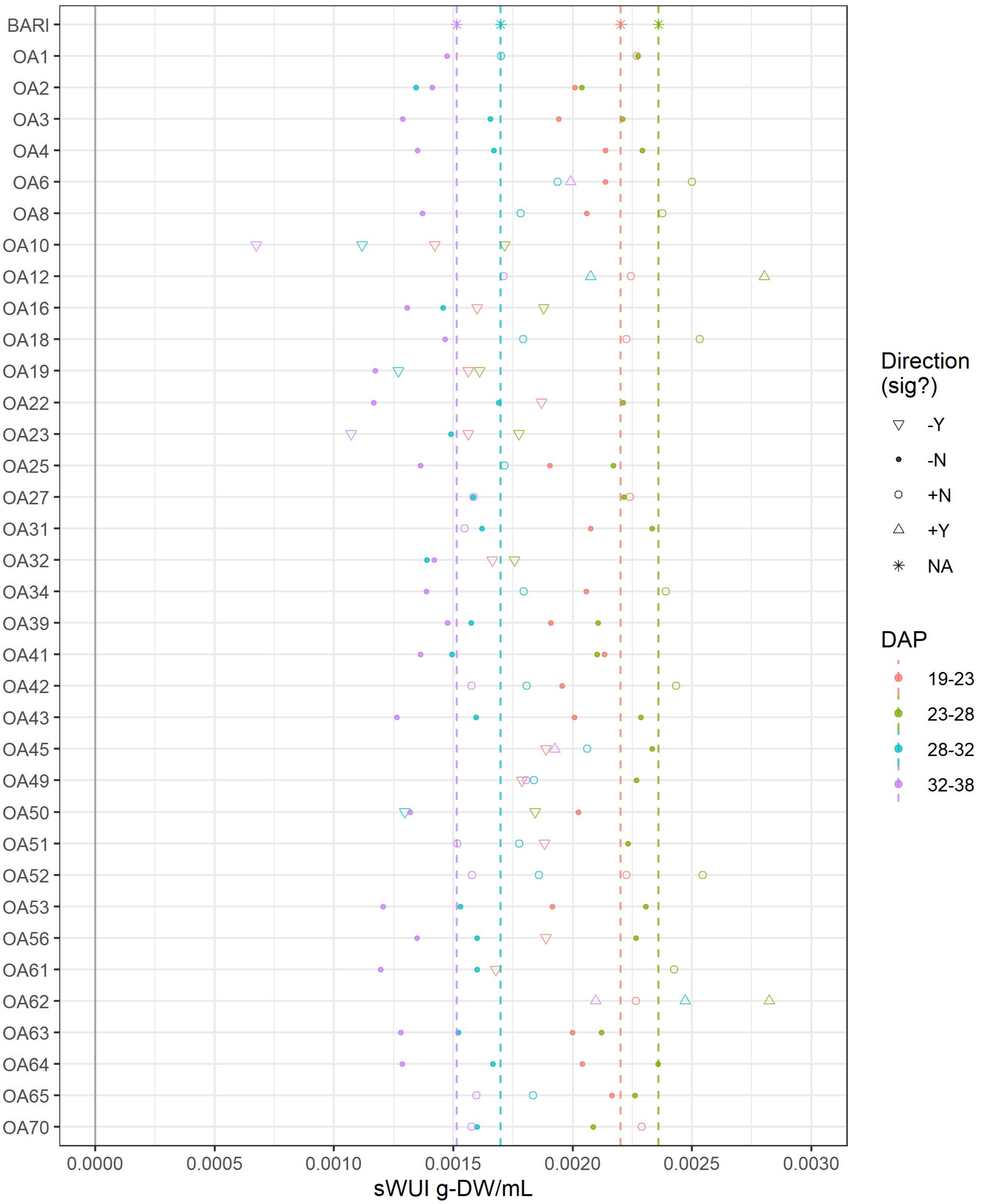

Figure S38.  NaCl 80mM

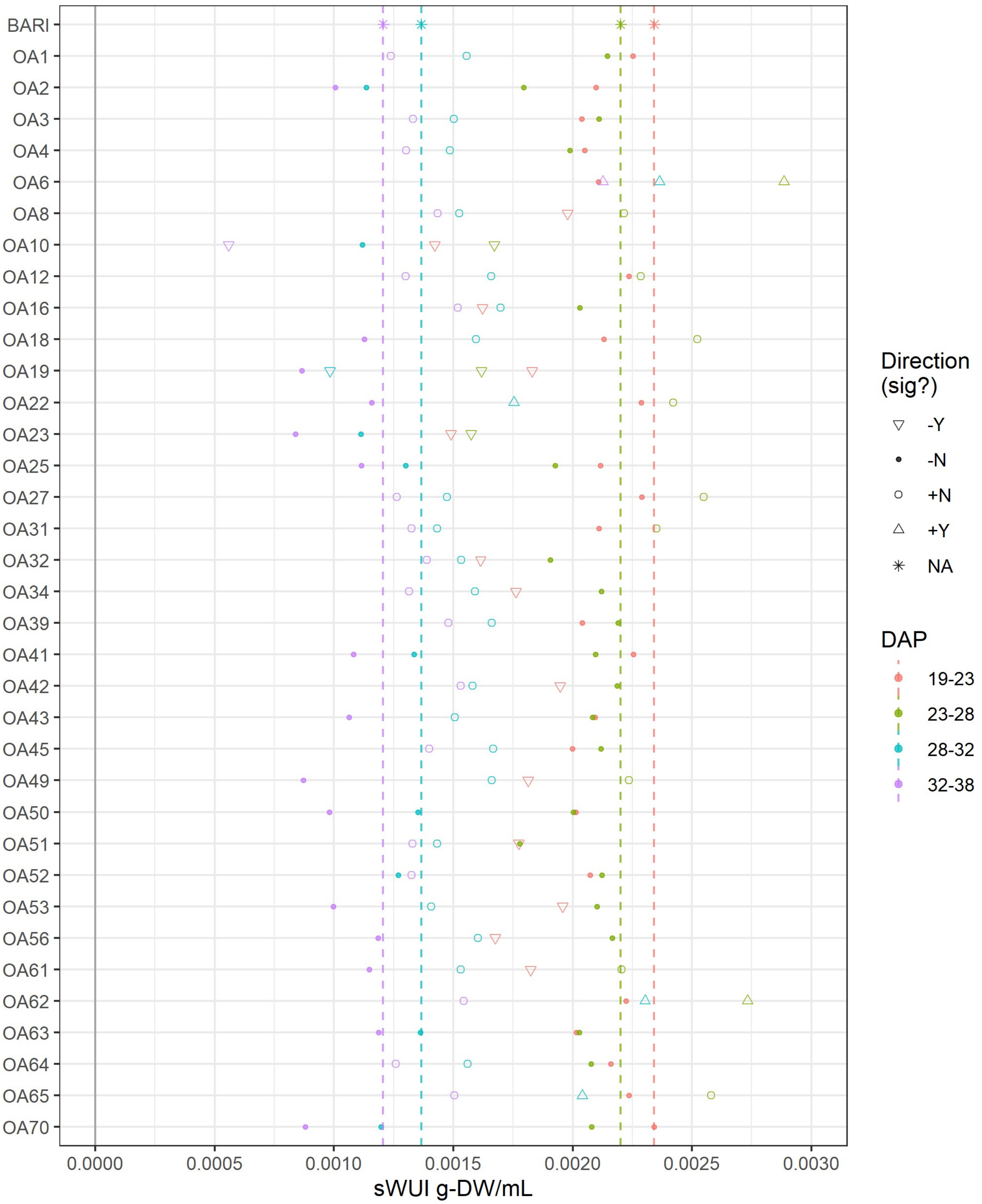

Figure S39. NaCl 120mM

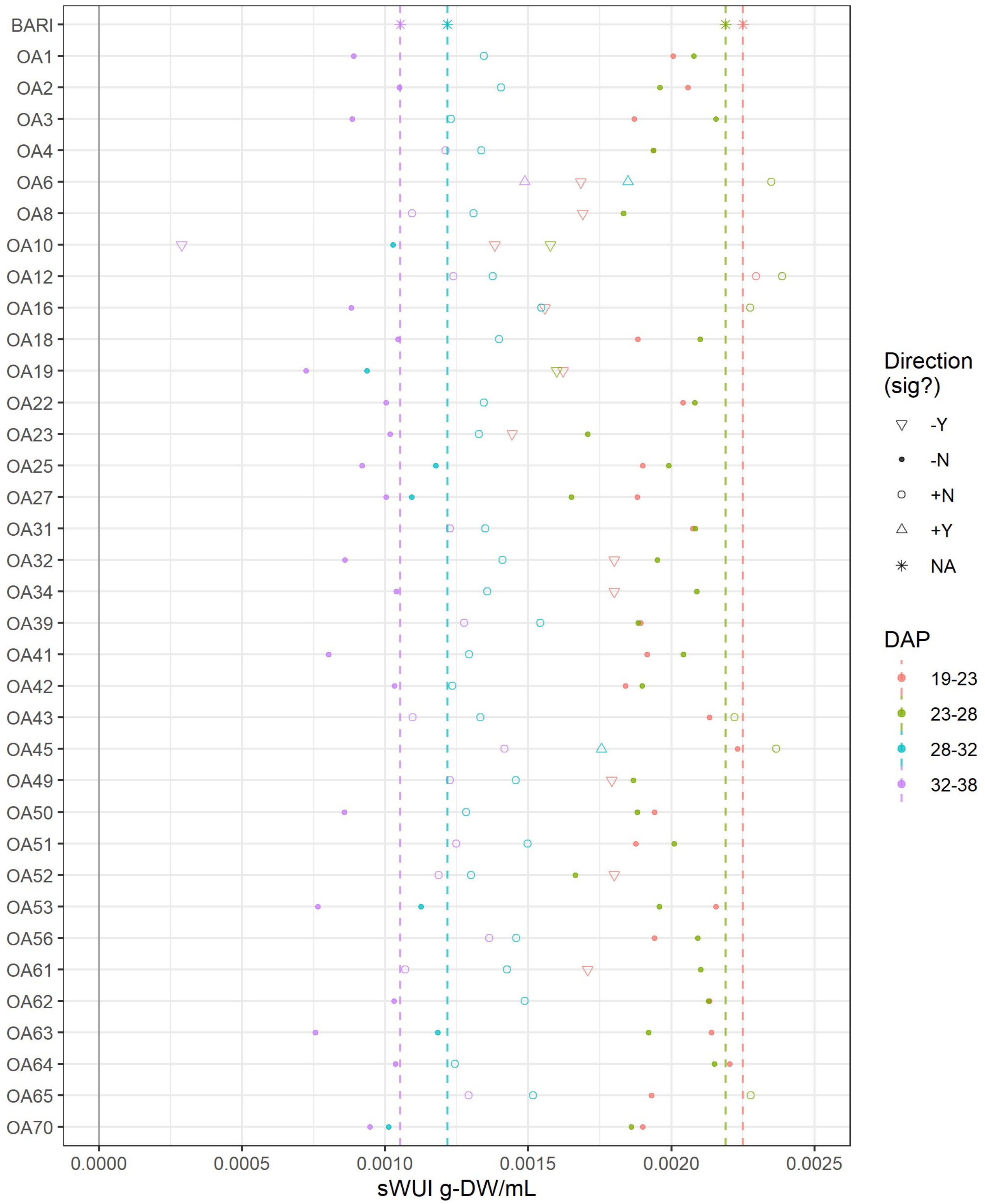

Figure S40.  NaCl 160mM

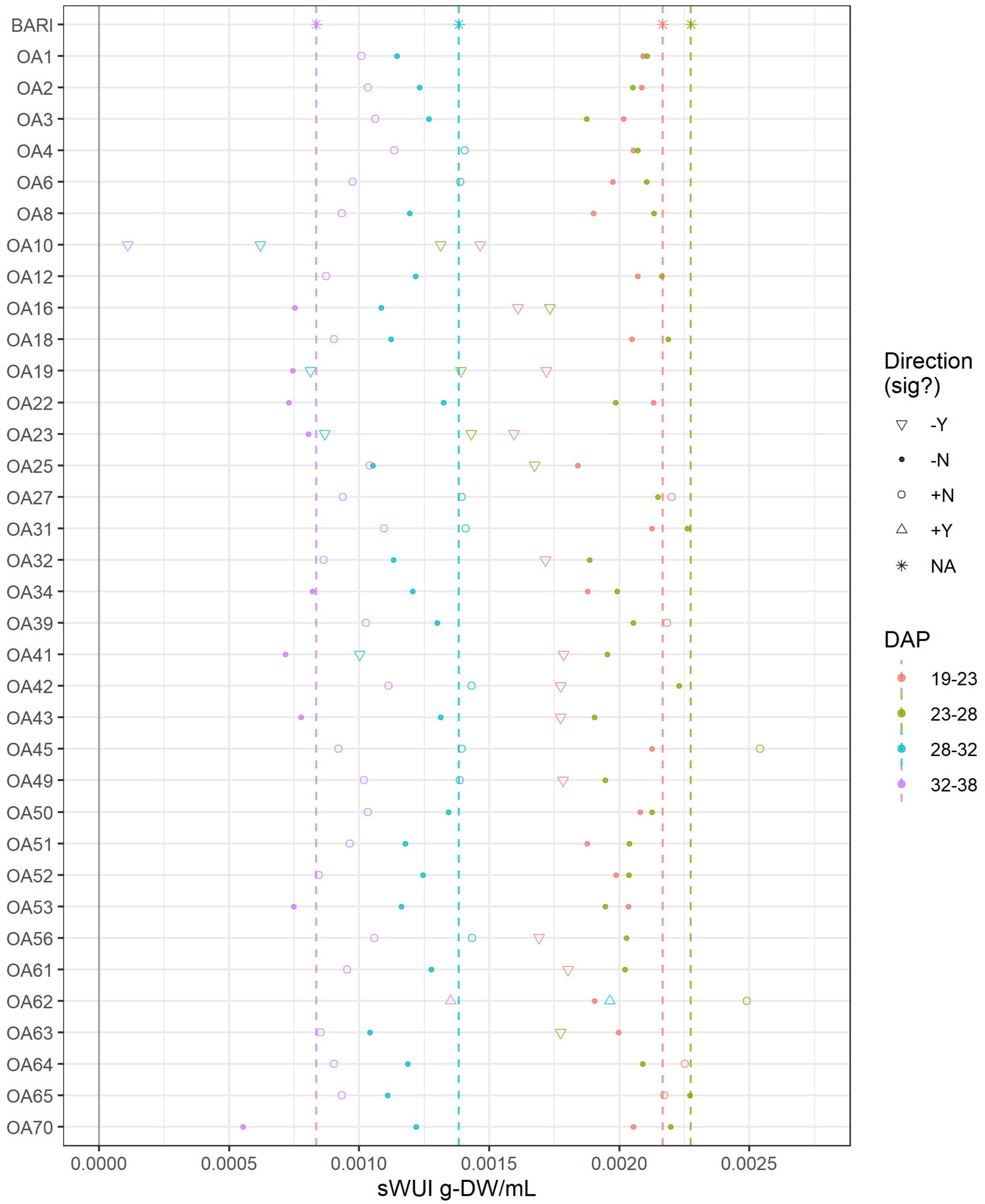

Figure S41.

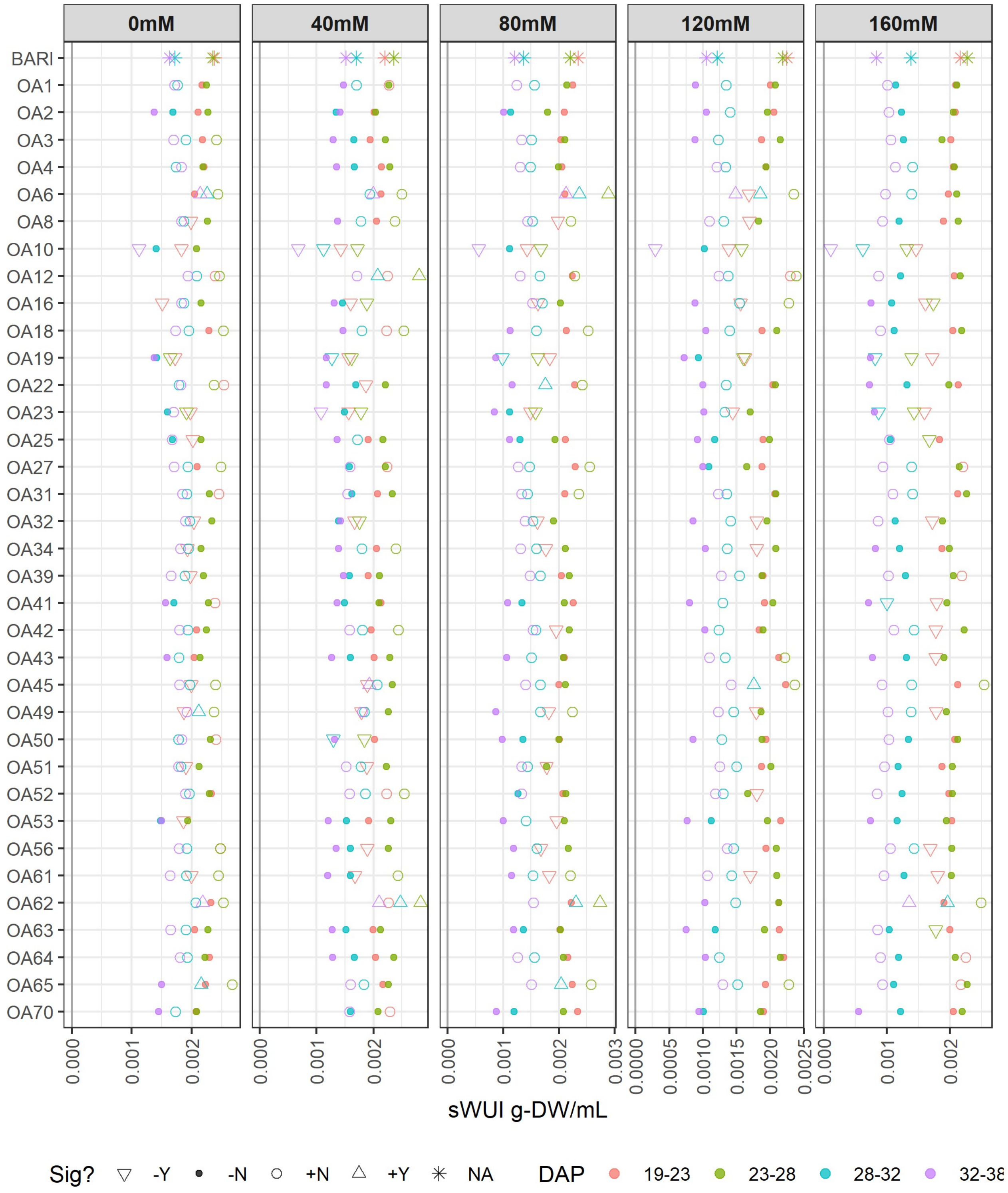

0mM
40mM
80mM
120mM
160mM
BARI
OA1
OA2
OA3
OA4
OA6
OA8
OA10
OA12
OA16
OA18
OA19
OA22
OA23
OA25
OA27
OA31
OA32
OA34
OA39
OA41
OA42
OA43
OA45
OA49
OA50
OA51
OA52
OA53
OA56
OA61
OA62
OA63
OA64
OA65
OA70
0.000
0.001
0.002
0.003
0.0000
0.0005
0.0010
0.0015
0.0020
0.0025
sWUI g-DW/mL
Sig?
-Y
-N
+N
+Y
NA
DAP
19-23
23-28
28-32
32-38

Figure S42.

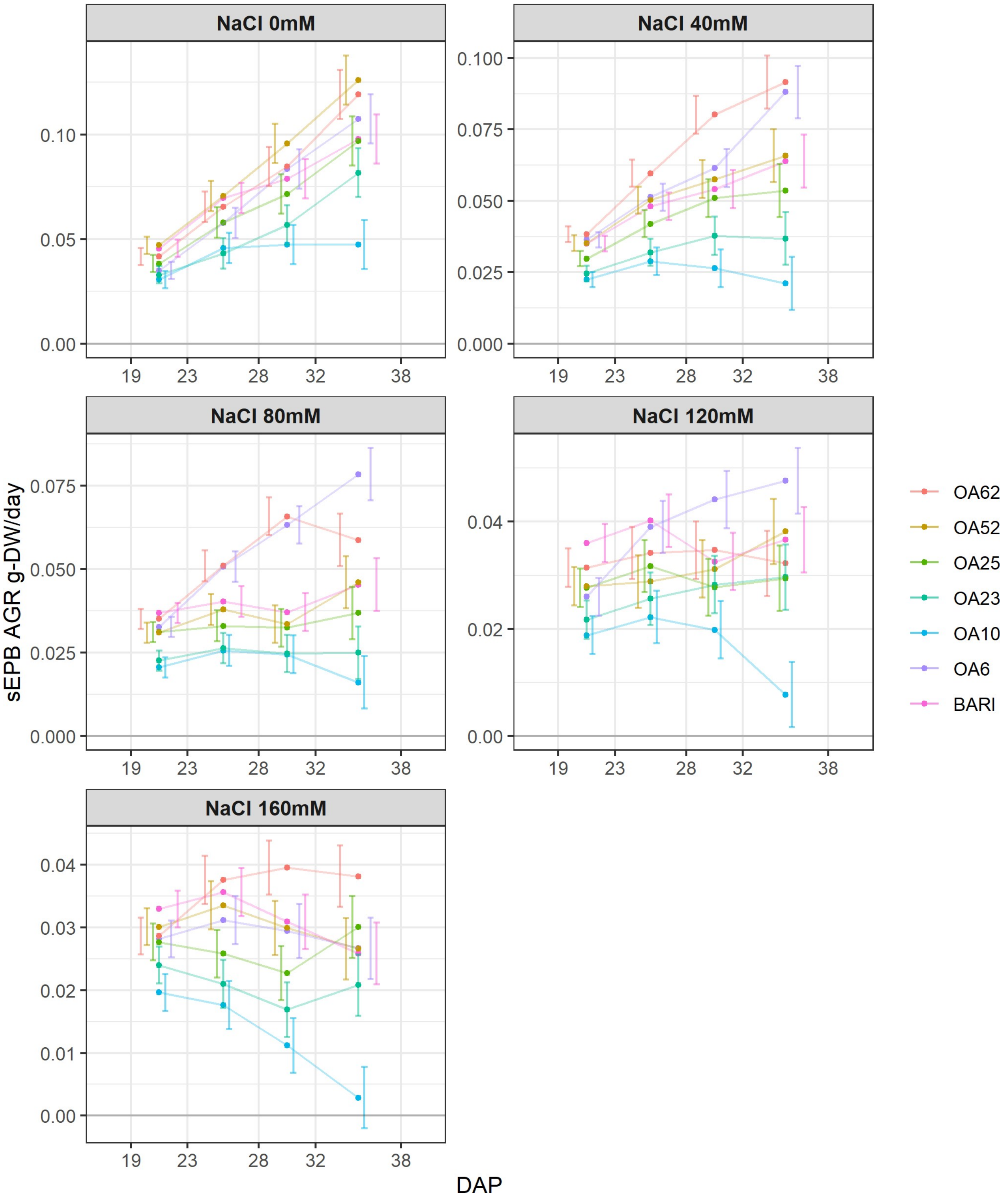


Predicted means for Absolute Growth Rate of smoothed Estimated Plant Biomass (sEPB AGR) plotted against days after planting (DAP) for seven Lines subjected to five salt concentrations. Error bars are predicted means ± half-LSDs (5%). Two predicted means for the same DAP are significantly different ($p \leq 0.05$) if their error bars do not overlap.

Figure S43.

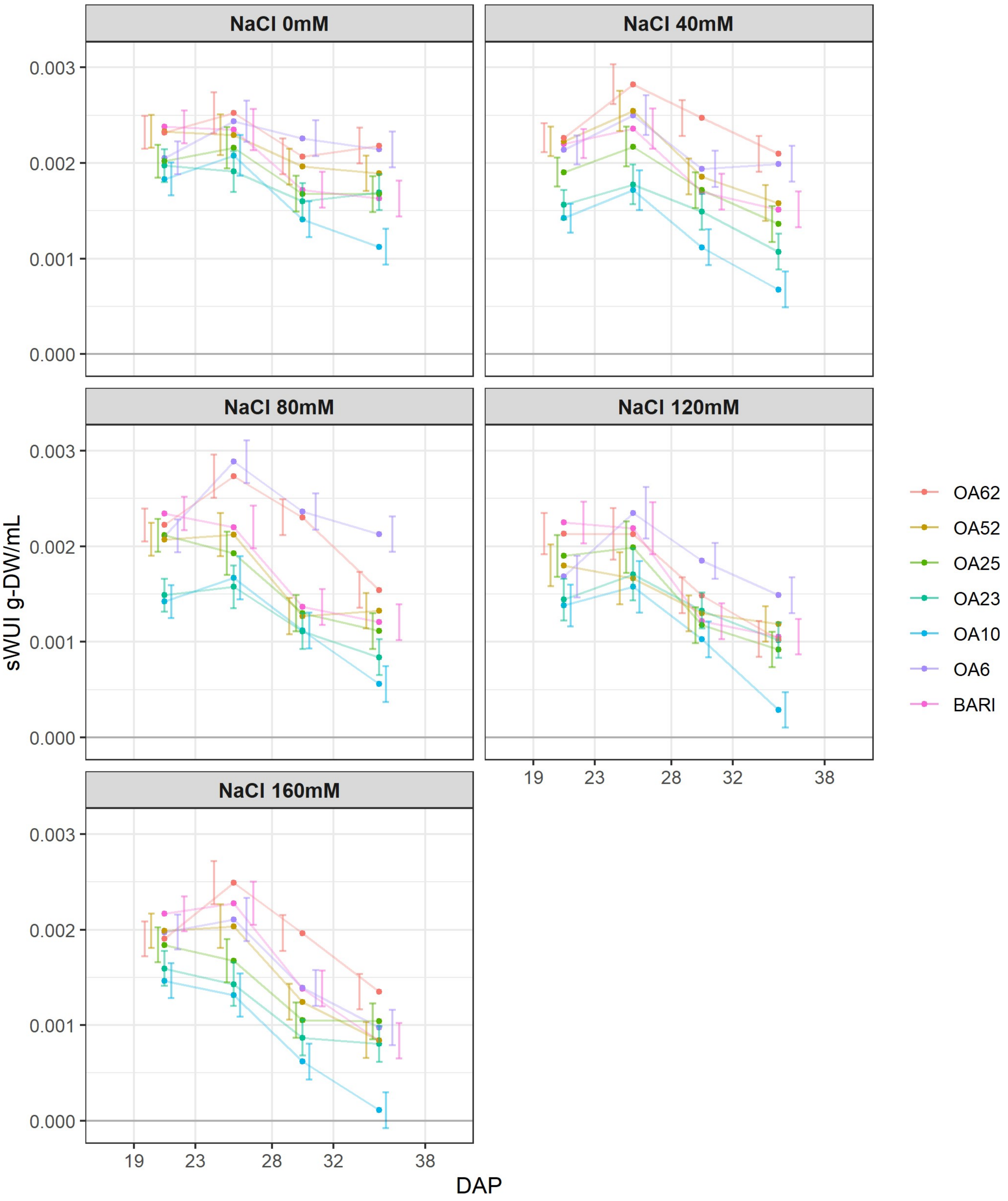


Predicted means for smoothed Water Use Index (sWUI) plotted against days after planting (DAP) for seven Lines subjected to five salt concentrations. Error bars are predicted means ± half-LSDs (5%). Two predicted means for the same DAP are significantly different (*p* ≤ 0.05) if their error bars do not overlap.

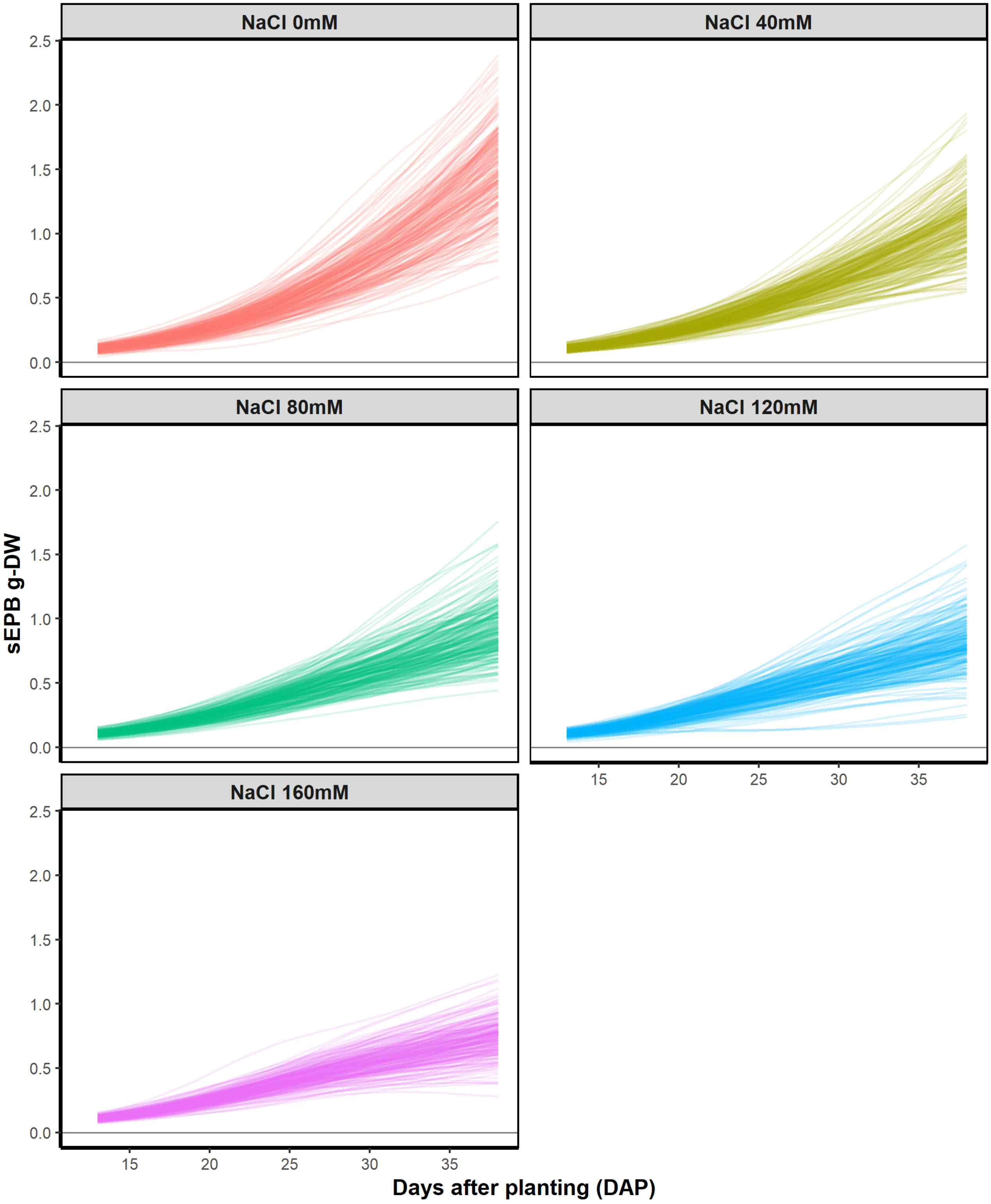


*Figure S44: Descriptive plots for sEPB ($n = 1075$, 1 curve per plant).*

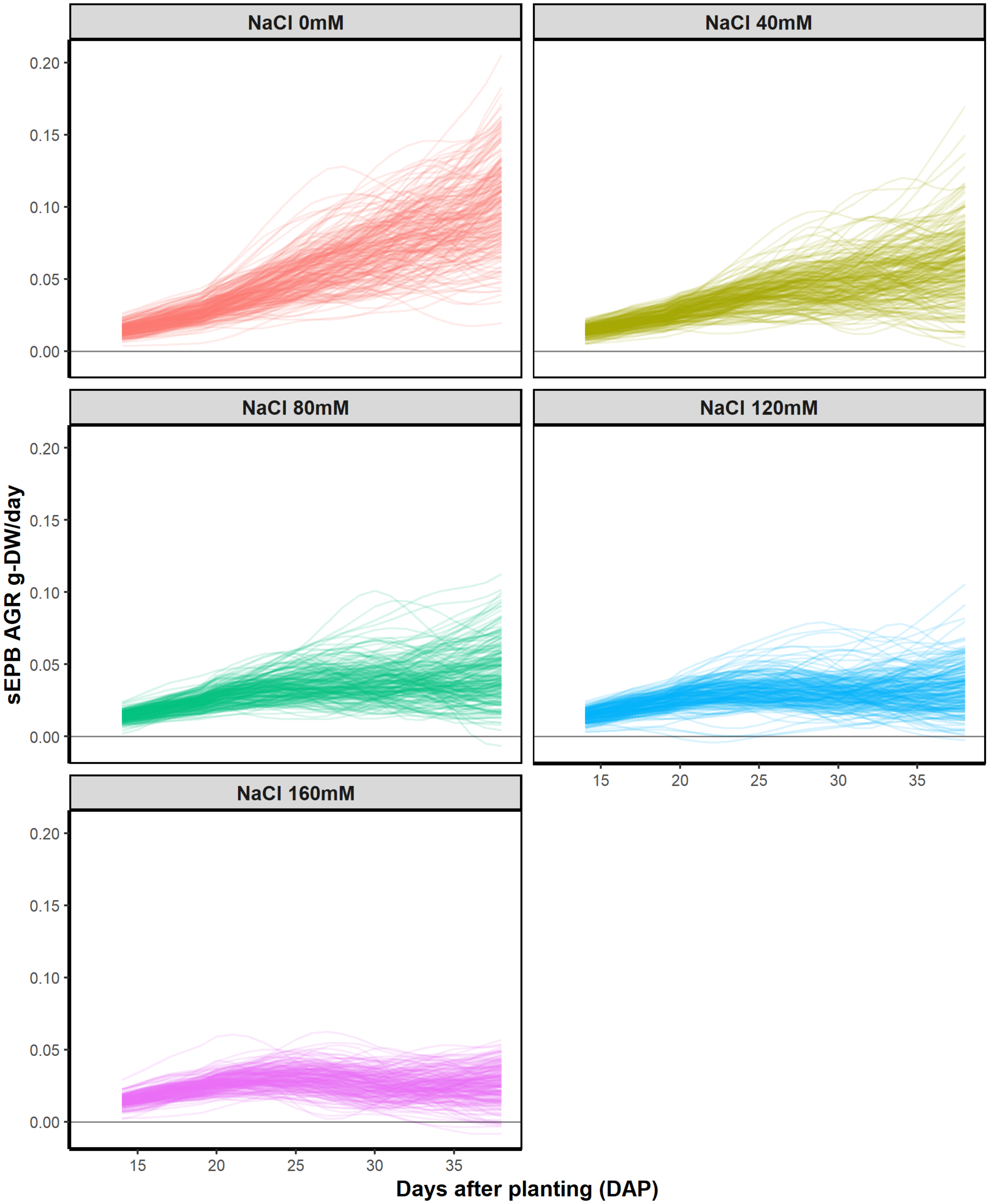


*Figure S45: Descriptive plots for sEPB AGR ($n = 1075$, 1 curve per plant).*

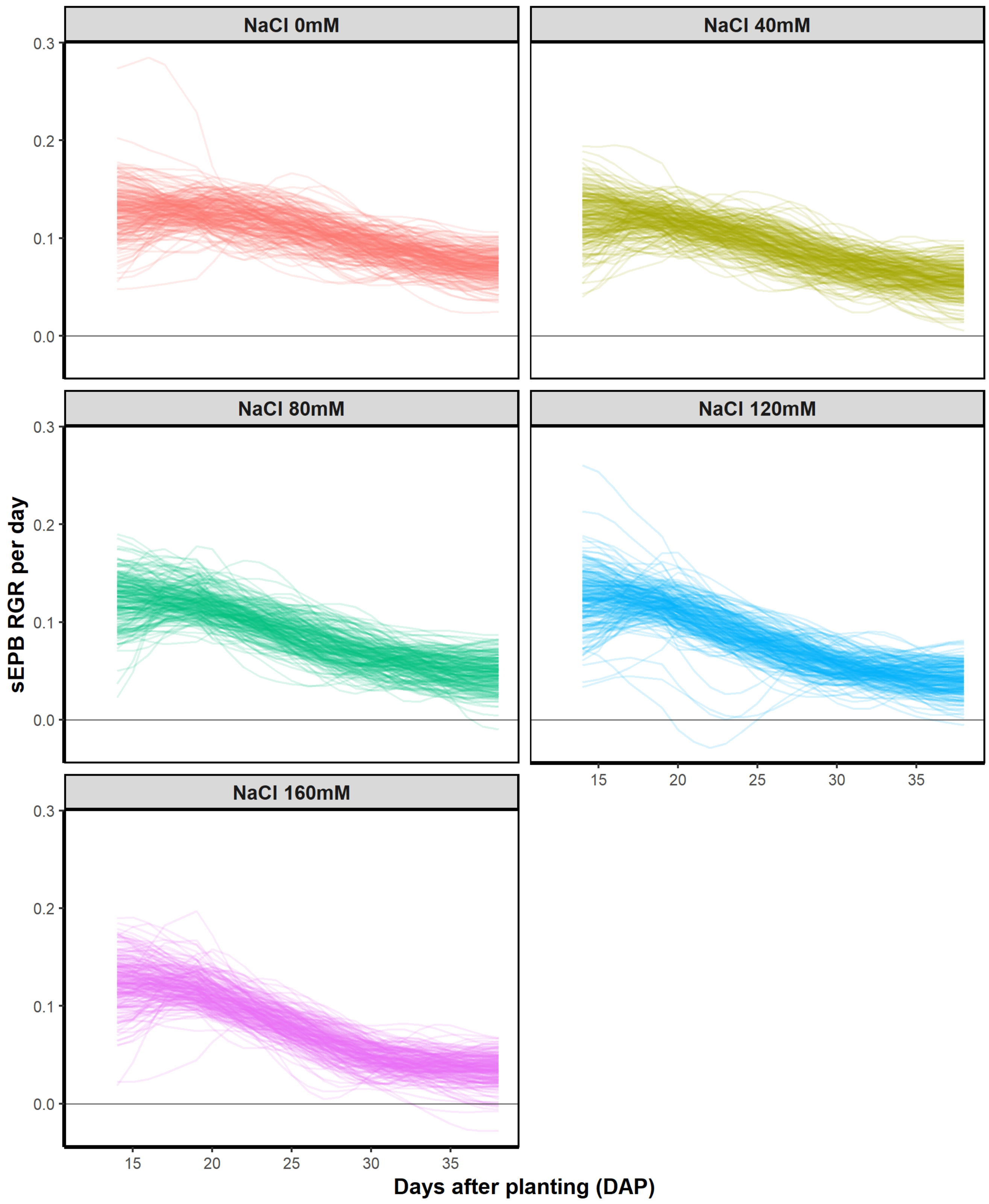


*Figure S46: Descriptive plots for sEPB RGR ($n = 1075$, 1 curve per plant)*
